# Supplementary material for: Mutated axon guidance gene PLXNB2 sustains growth and invasiveness of stem cells isolated from cancers of unknown primary
Source: EMBO Mol Med. 2023 Feb 1;15(3):e16104. doi: 10.15252/emmm.202216104 (PMC9994481; doi:10.15252/emmm.202216104)
Supplement: Supplementary file 1 — Appendix [file EMMM-15-e16104-s004.pdf]

Brundu et al.

Mutation of the axon guidance gene PLXNB2 sustains proliferative autonomy and confers invasive properties to stem cells isolated from Cancers of Unknown Primary

APPENDIX

Table of Contents

- **Appendix Figure S1.** Histological images of CUP samples analyzed in this study *Page 2*

- **Appendix Figure S2.** Supplemental data on cell collapsing assays with PlnxB2 mutants *Page 3*

- **Appendix Figure S3.** Ligand binding assays comparing wild-type and G842C-PlxnB2 *Page 4*

- **Appendix Figure S4.** Supplemental images of cell migration/invasion assays *Page 5*

Appendix Figure S1

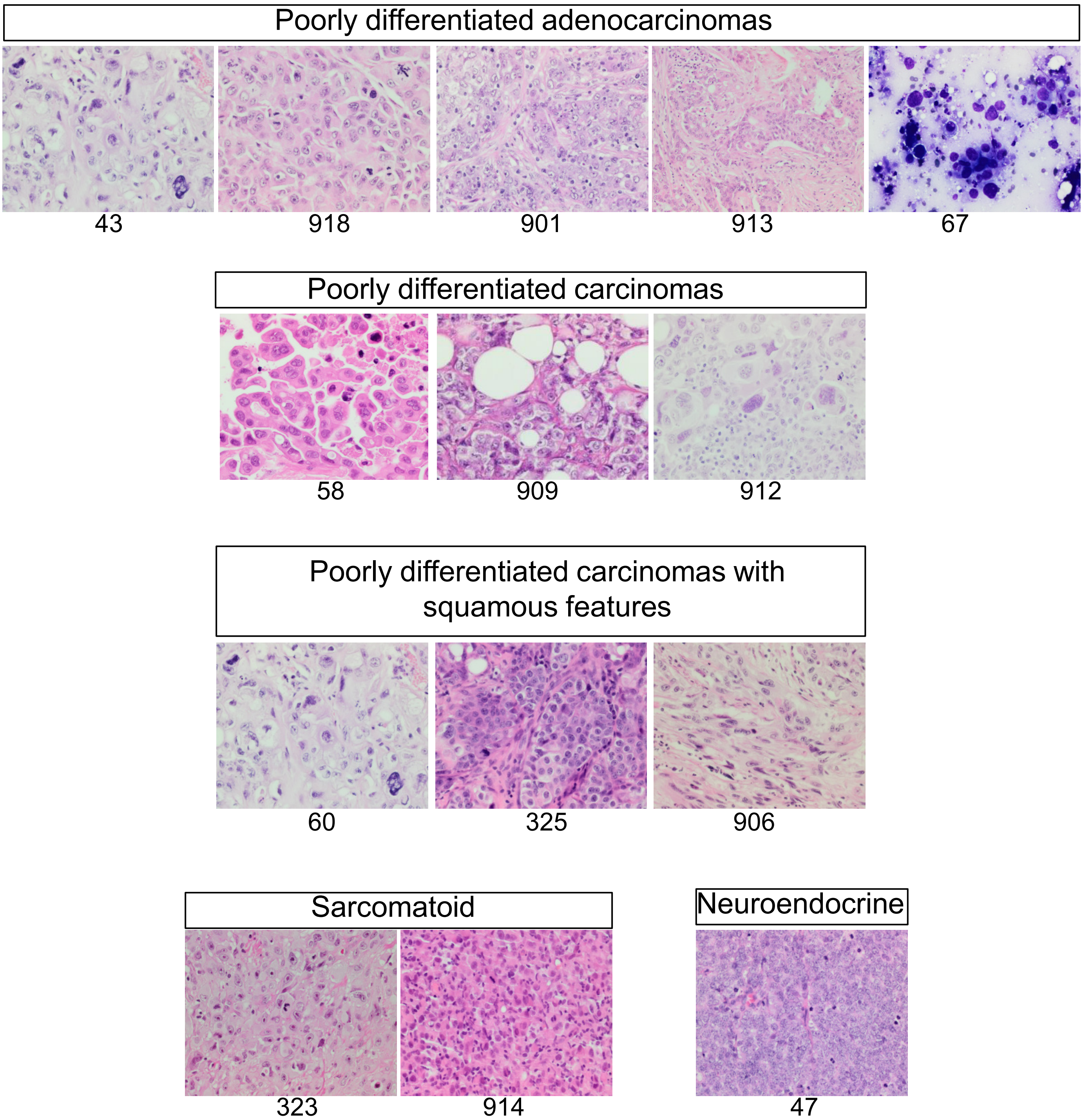

Appendix Fig. S1. Histological images of CUP samples analyzed in this study. Histological images (40x) of sectioned paraffin-embedded CUP samples (e.g. AGN43, AGN918, etc., as indicated in Table EV1), stained by Hematoxylin-Eosin staining, except for panel N (AGN67) which is a cytological specimen stained by Diff-Quick.

Appendix Fig. S2. Collapsing assays with PlnxB2 mutants. Immunofluorescence analysis of transfected COS7 cells expressing wild-type or mutant PlxnB2 variants. Cells expressing the WT receptor were also stimulated with 1 μg/ml Sema4C, in order to provide an internal positive control of the typical collapsing response. The area of around 50-100 representative cells per each condition (from multiple independent microscopic fields and experiments) was measured by ImageJ software, and the individual values were included in a violin plot (shown on the right), where the red line indicates the median value and yellow lines mark the first and third quartiles of the population. The statistical analysis was done by one-way ANOVA, comparing each of the groups with that of unstimulated WT cells: ****p<0.0001; *p<0.05.

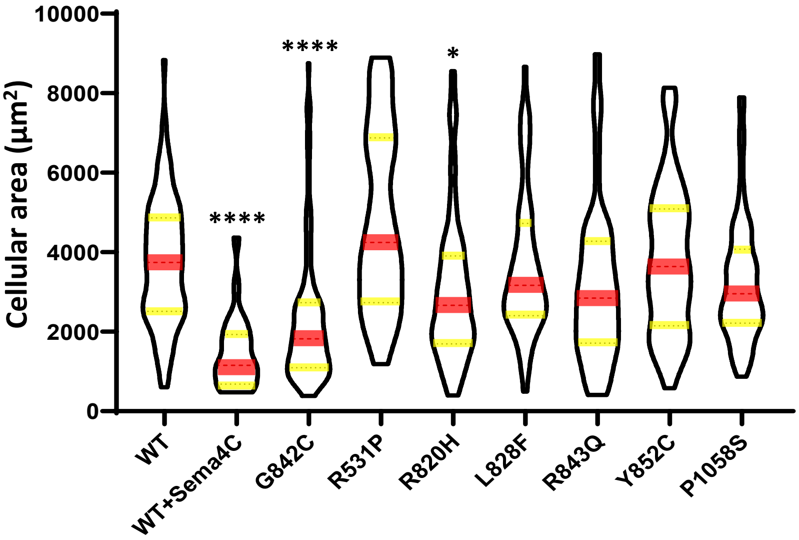

Below is shown a wide series of representative fields for the diverse conditions, presented as pairs of one image displaying anti-PlxnB2 staining in red, and one corresponding merge of fluorescent channels (including green staining of F-actin by phalloidin, and nuclear DAPI staining in blue). Scale bar: 50μm.

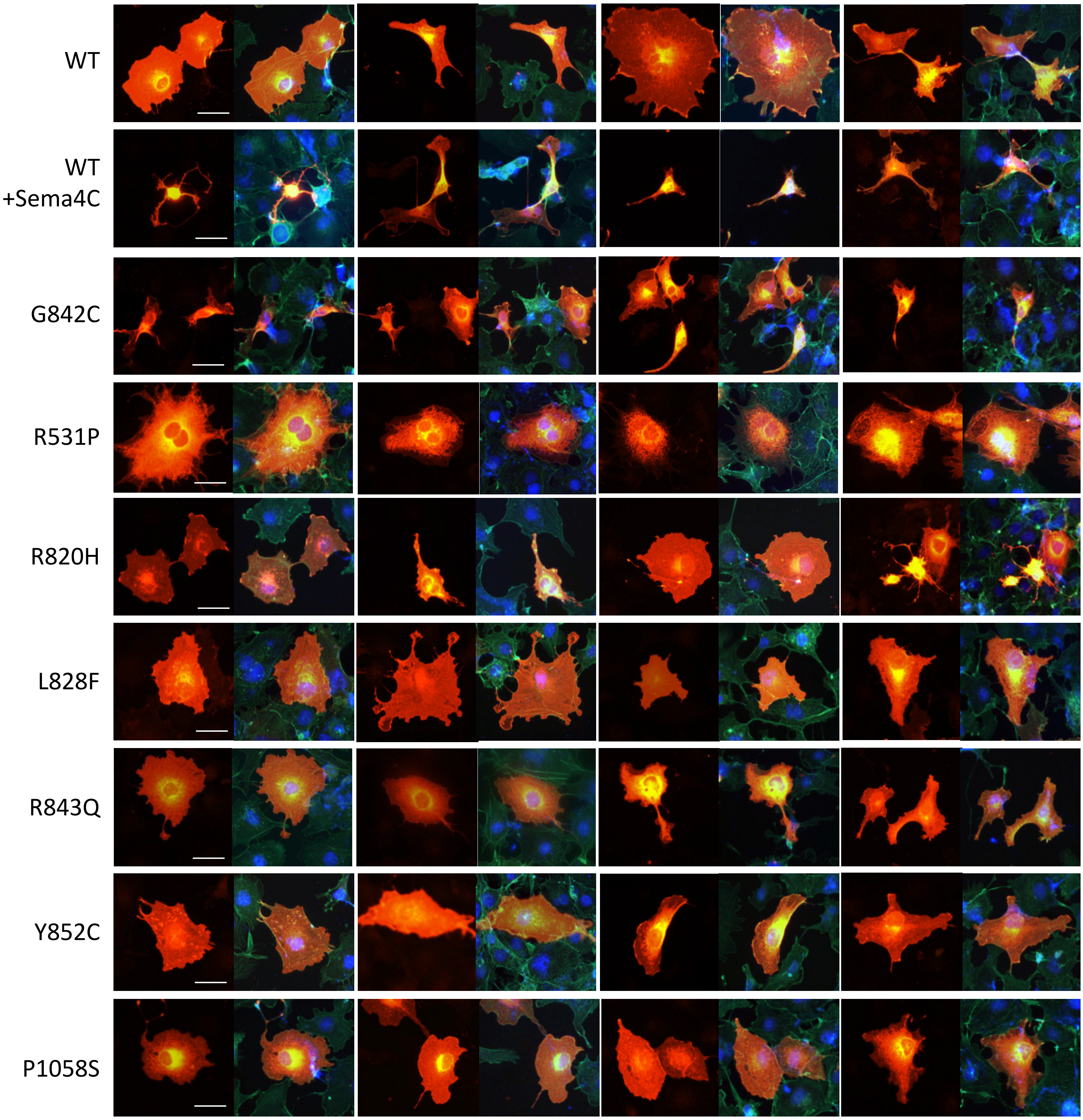

Appendix Figure S3

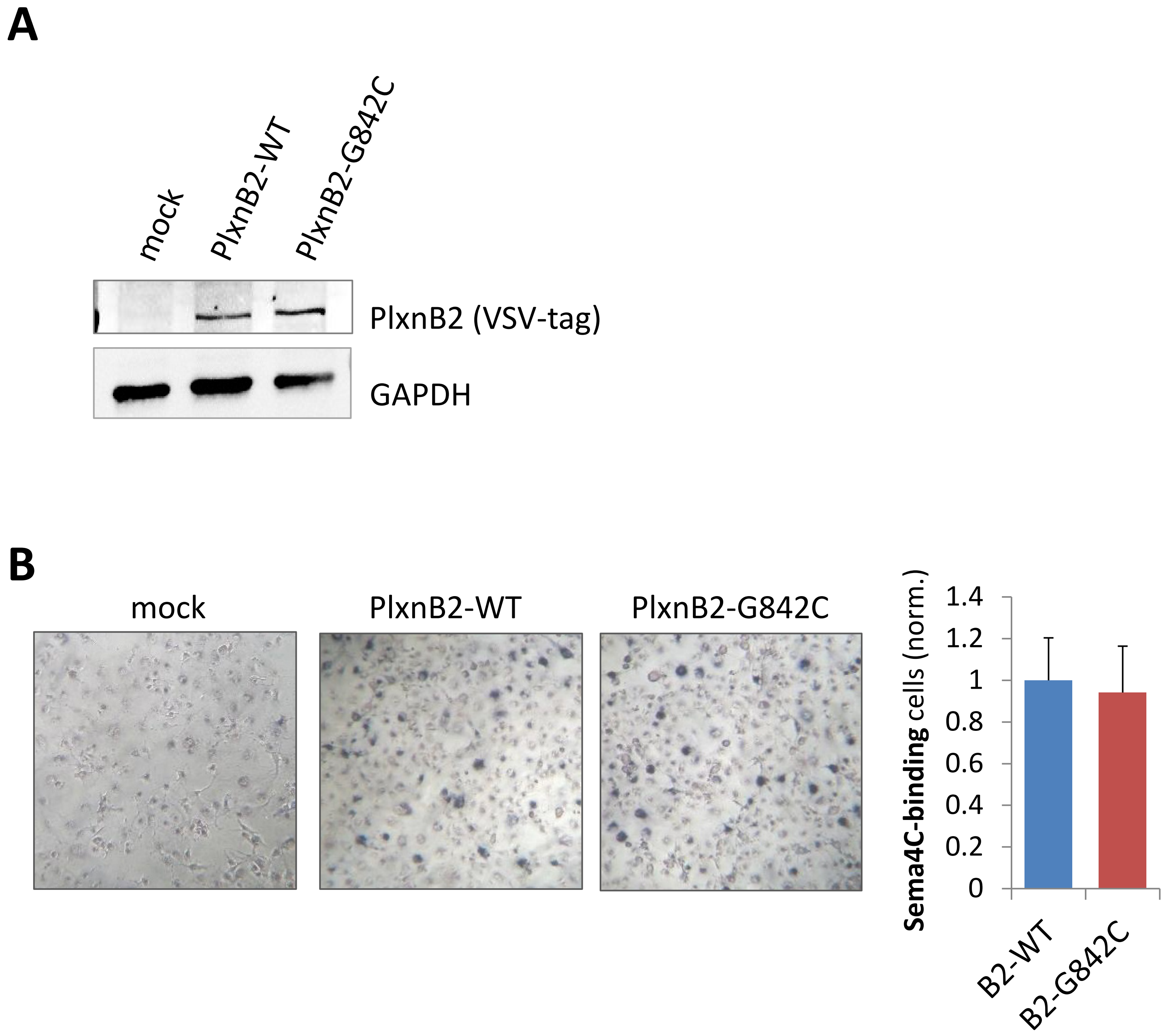

Appendix Fig. S3. Ligand binding assays comparing wild-type and G842C-PlxnB2

(A) The comparable expression of VSV-tagged wild-type (WT) and mutated PlxnB2 in transfected COS7 cells was verified by western blotting.

(B) Representative images of COS7 cells transfected with WT or G842-mutated PlxnB2 (analyzed in the previous panel) and probed with alkaline phosphatase-conjugated Sema4C, its cognate ligand, revealing comparable receptor binding. The graph at the bottom shows normalized mean values ± SD of the AP-labeled cell quantified in three experiments (after subtracting mock background signal).

Appendix Figure S4

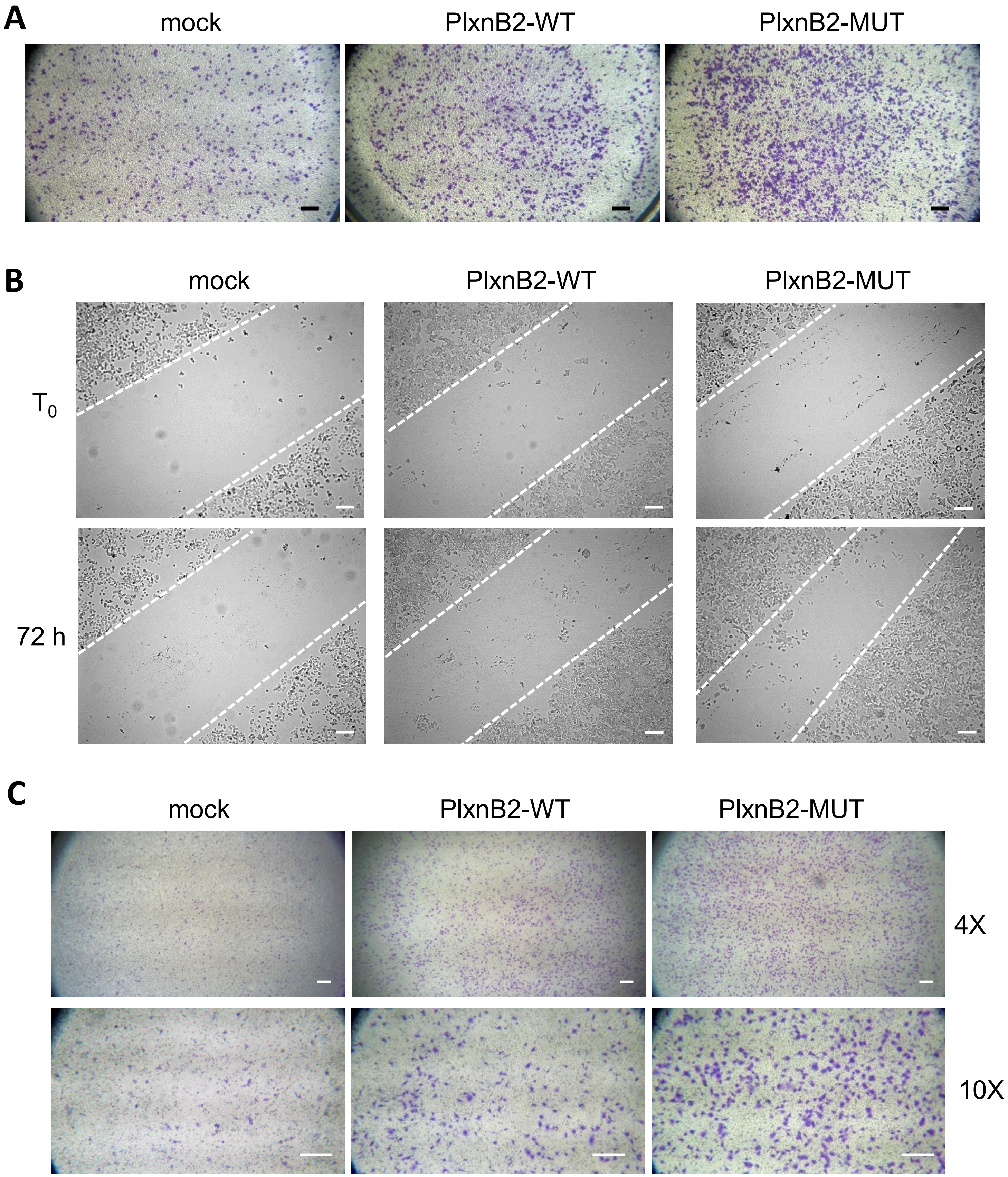

Appendix Fig. S4. Supplemental images of cell migration and cell invasion assays.
(A) Representative microscopic images of MCF-7 cells, either mock transfected, or overexpressing WT or G842C-mutated PlxnB2, migrated through Transwell inserts, then fixed and stained with crystal violet. Scale bar: 200 μm. Data quantification and statistical analysis across multiple replicates is shown in main Fig. 7B. (B) Representative phase contrast microscopic images of monolayers of the same MCF-7 cells as above (either mock, or overexpressing WT or G842C-mutated PlxnB2) immediately after scratching at T0, and then after 72 hours wound closure. Scale bar: 200 μm. Data quantification and statistical analysis across multiple replicates is shown in main Fig. 7C. (C) Representative microscopic images (at 4x or 10x magnification) of AS906 cells (either mock, or overexpressing WT or G842C-mutated PlxnB2), which had invaded across Matrigel-coated Transwell inserts, and were then fixed and stained with crystal violet. Scale bars: 200 μm. Data quantification and statistical analysis across multiple replicates is shown in main Fig. 7E.
