# Supplementary material for: Mutated axon guidance gene PLXNB2 sustains growth and invasiveness of stem cells isolated from cancers of unknown primary
Source: EMBO Mol Med. 2023 Feb 1;15(3):e16104. doi: 10.15252/emmm.202216104 (PMC9994481; doi:10.15252/emmm.202216104)
Supplement: Supplementary file 2 — Expanded View Figures PDF [file EMMM-15-e16104-s008.pdf]

Expanded View Figures

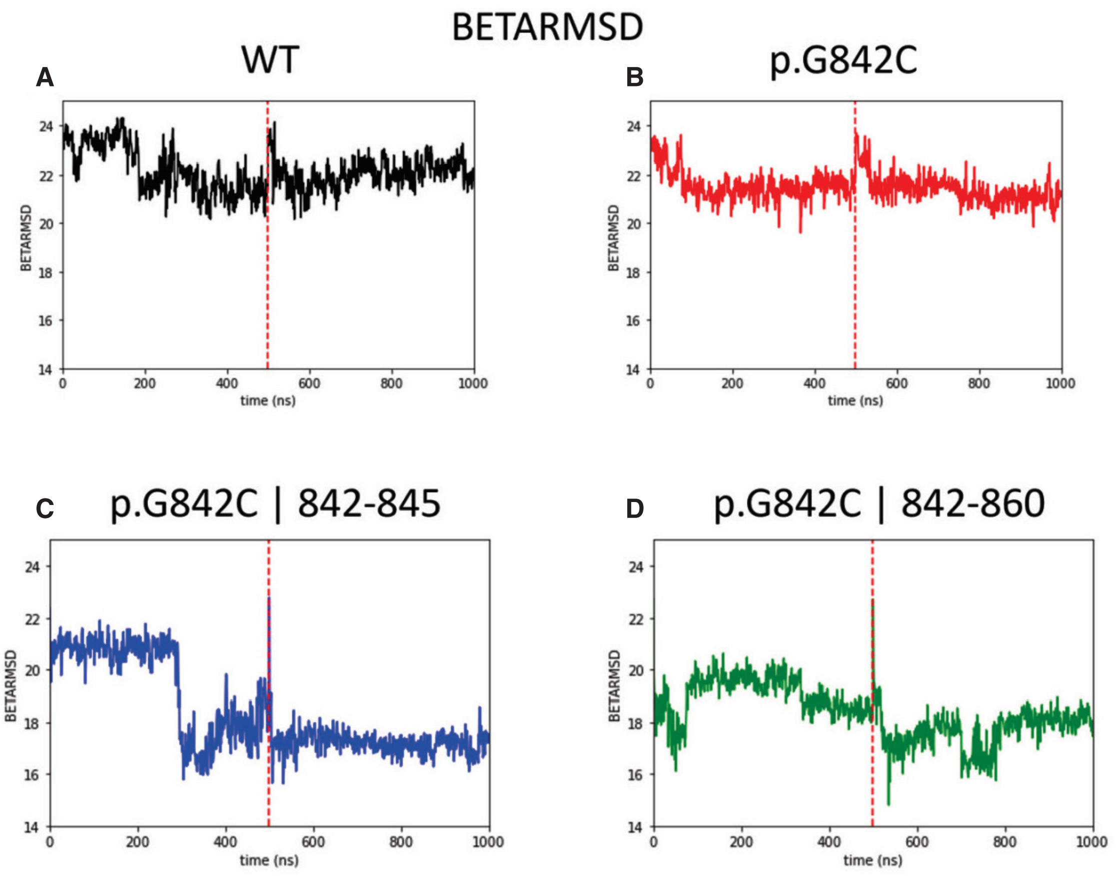

Figure EV1. BETARMSD plots of WT PlxnB2 and G842C-mutated isoforms.

A–D BETARMSD plots of the concatenated replicas of PlxnB2 WT, p.G842C, p.G842C 842–845, and p.G842C 842–860, respectively. The plots report the beta-sheet content evolution among the four studied systems, using the BETARMSD score described in the Materials and Methods section. In panel A, it is reported the time course of the selected parameter for the two concatenated MD simulations of the wild-type protein. It is evident that, starting from the initial state, a small rearrangement of the secondary structure is present even if the parameter remains quite stable along the simulation as identified by the average value obtained that is <BETARMSDWT> = 21.9 ± 0.5. The behavior of the same parameter for the point mutation of G842 in a cysteine residue, still maintaining the native disulfide bond, is reported in panel B, and it shows a slight decrease of beta-sheet content (<BETARMSDp.G842C> = 20.4 ± 0.4), compared with WT due to the loss of a single beta strand (βG, as it is shown in Fig 3A in the main text). The same point mutation coupled with alternative disulfide bonds leads to a more relevant loss of beta-sheet content in both cases (<BETARMSDp.G842C|842–845> = 17.6 ± 1.19, <BETARMSDp.G842C|842–860> = 18.4 ± 0.80) due to the disruption of βG, βC, and βD strands (see Fig 3D and E in the main text).

Figure EV2. RMSD plots of additional PlxnB2 ectodomain mutants found in human tumors.

A–D RMSD plots of two independent repetitions of 250 ns molecular dynamics of diverse PlxnB2 IPT3 domain mutants found in human tumors. (A) Mutant R820H; (B) mutant L828F; (C) mutant R843Q; (D) mutant Y852C.

E, F RMSD plots of two independent repetitions of 250 ns molecular dynamics of additional PlxnB2 mutants (outside IPT3 domain) found in CUP samples: (E) R531P and (F) P1058S.

G, H RMSD plots of two independent repetitions of 500 ns molecular dynamics of wild-type PlxnB2 and G842C mutant, extracted from Fig 3 and reported here for internal reference.

© 2023 The Authors

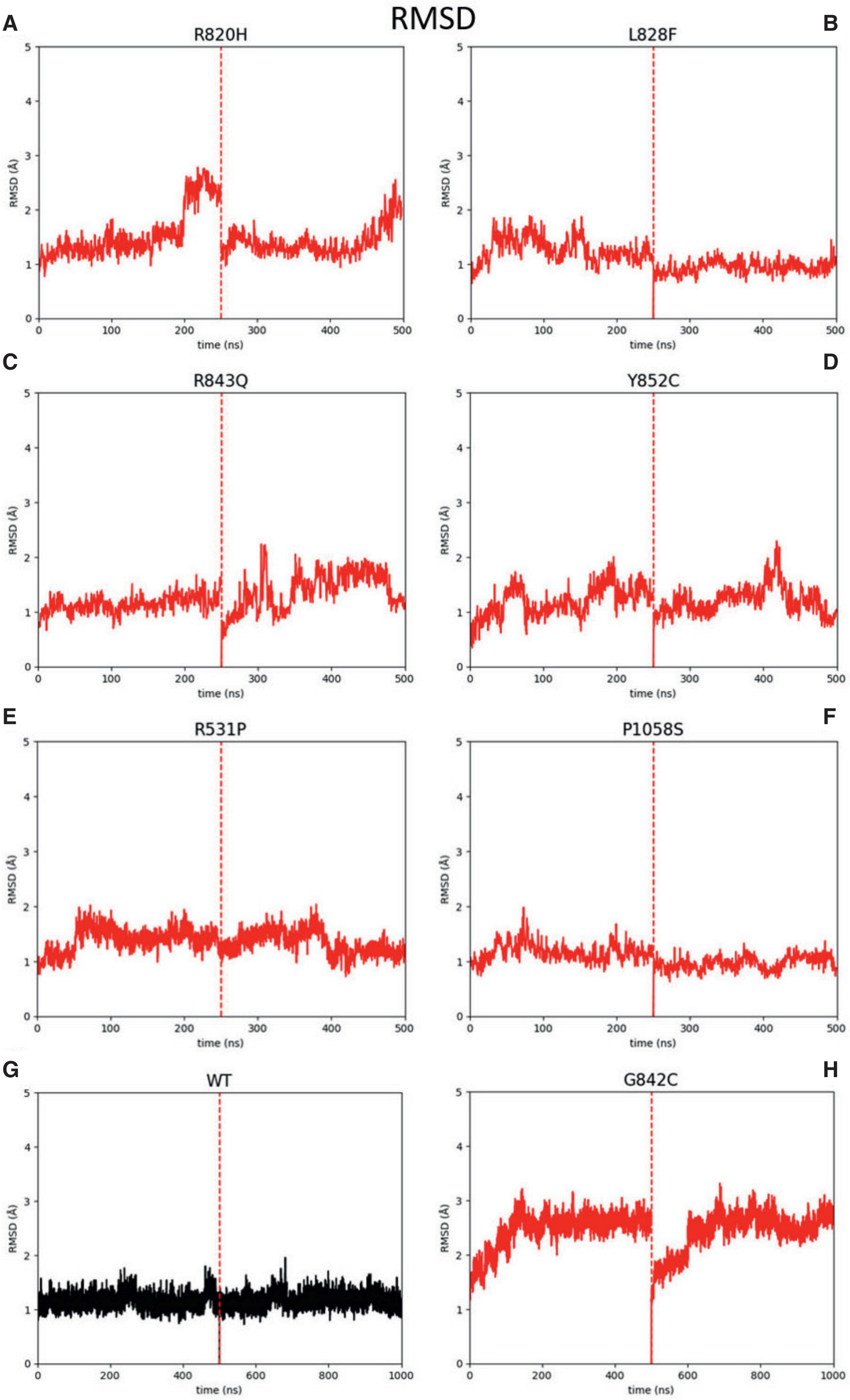

Figure EV2.

 © 2023 The Authors

Figure EV3. Validation of CUP models with modified expression of PlxnB2 WT or G842C mutant.

A Western blotting analysis of PlxnB2 expression in the indicated CUP-derived agnospheres, either subjected to gene knockdown by lentiviral-mediated transfer of targeted shRNAs (shPlxnB2) or transduced with a nontargeting control sequence (shScramble).

B Western blotting analysis of PlxnB2 expression in CUP-derived AS67, engineered by lentiviral-mediated transfer of an empty vector control (mock), nontargeting scrambled shRNAs, PlxnB2-targeted shRNAs, a wild-type PlxnB2 expression construct, or a G842C-mutated PlxnB2 expression construct PlxnB2-G842C, respectively.

C Western blotting analysis of PlxnB2 expression in AS901 and AS906 (as indicated), engineered to overexpress either wild-type PlxnB2 or PlxnB2-G842C, or transduced with an empty vector (mock).

D Western blotting analysis of PlxnB2 expression in AS43 subjected to gene knockdown by lentiviral-mediated transfer of an alternative independent shRNA sequence (shPlxnB2 #2, see Materials and Methods), or transduced with a corresponding empty vector (shCtrl).

E Time course analysis of cellular viability in shPlxnB2-tranduced and control agnospheres described in the previous panel, over 7 days of growth in culture. Values are mean ± SD of $n = 3$ independent experiments (with quadruplicate technical replicates). The statistical significance was assessed by 2-way ANOVA multiple comparisons for each time point, with Bonferroni correction. shCtrl vs. shPlxnB2#2(1): at day 3 **$P = 0.0086$, day 4 **$P = 0.0047$; ****$P < 0.0001$. shCtrl vs. shPlxnB2#2(2): $^{\$}P = 0.0161$; $P < 0.0001$.

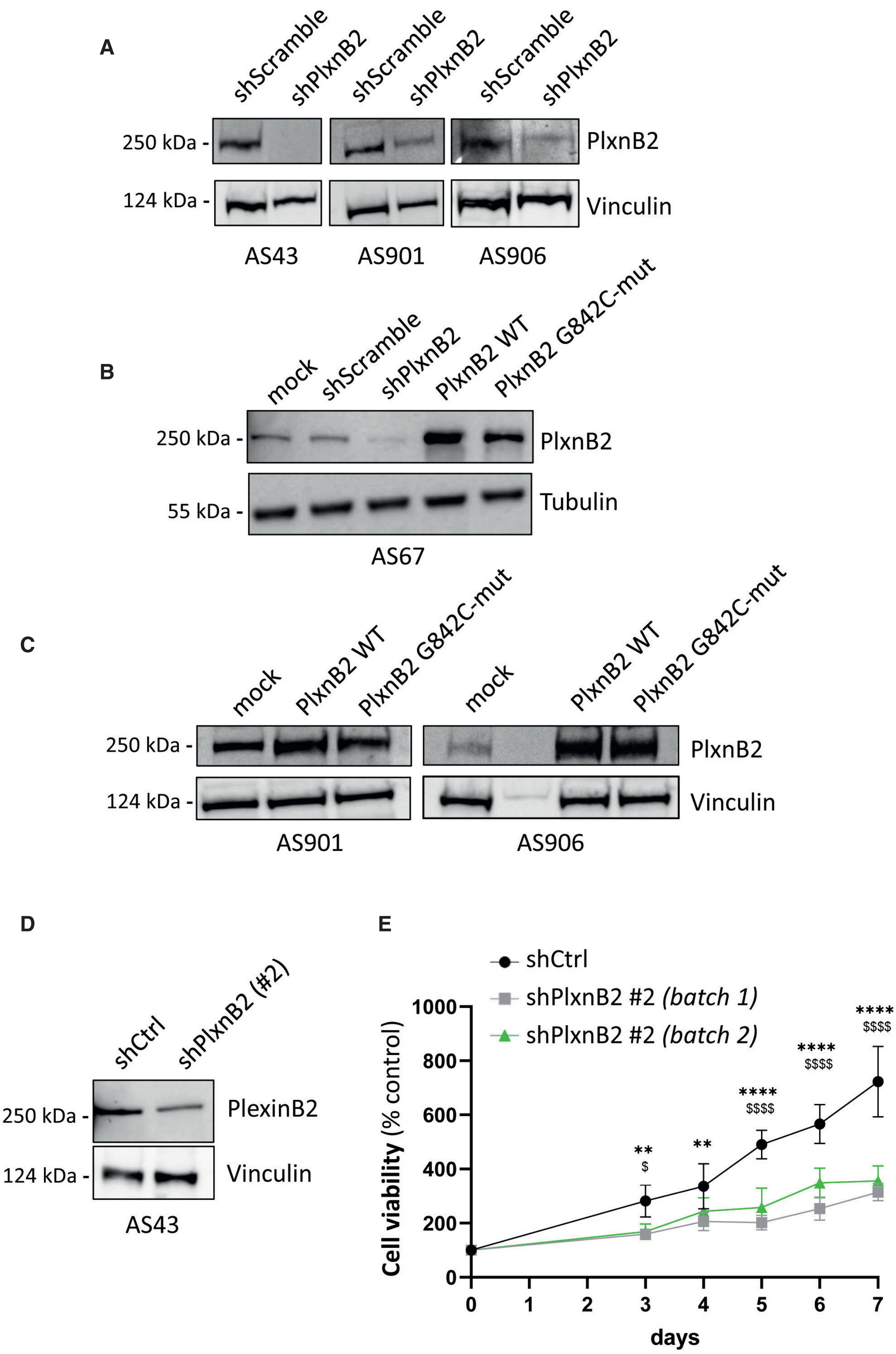

Figure EV3.

© 2023 The Authors

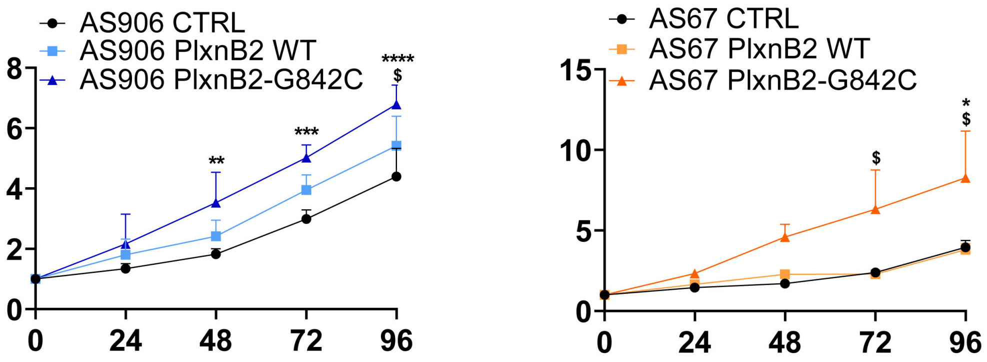

Figure EV4. Differential regulation of CUP cell growth by WT PlxnB2 or G842C mutant.

Time course cell viability analysis of AS906 (on the left) and AS67 (on the right) overexpressing either wild-type or PlxnB2-G842C, or mock controls transduced with an empty vector. Plotted values are the mean ± SD of $n = 3$ independent experiments (six technical replicates for each). The statistical significance was assessed by two-way ANOVA test. AS906 PlxnB2-G842C vs. controls (CTRL): at 48 h $^{**}P = 0.0042$, 72 h $^{***}P = 0.0007$, 96 h $^{****}P < 0.0001$. AS906 PlxnB2-G842C vs. PlxnB2-WT: at 96 h $^{\$}P = 0.0224$. AS67 PlxnB2-G842C vs. controls (CTRL): at 96 h $^{*}P = 0.0337$. AS67 PlxnB2-G842C vs. PlxnB2-WT: at 72 h $^{\$}P = 0.0494$, 96 h $^{\$}P = 0.0274$.

© 2023 The Authors

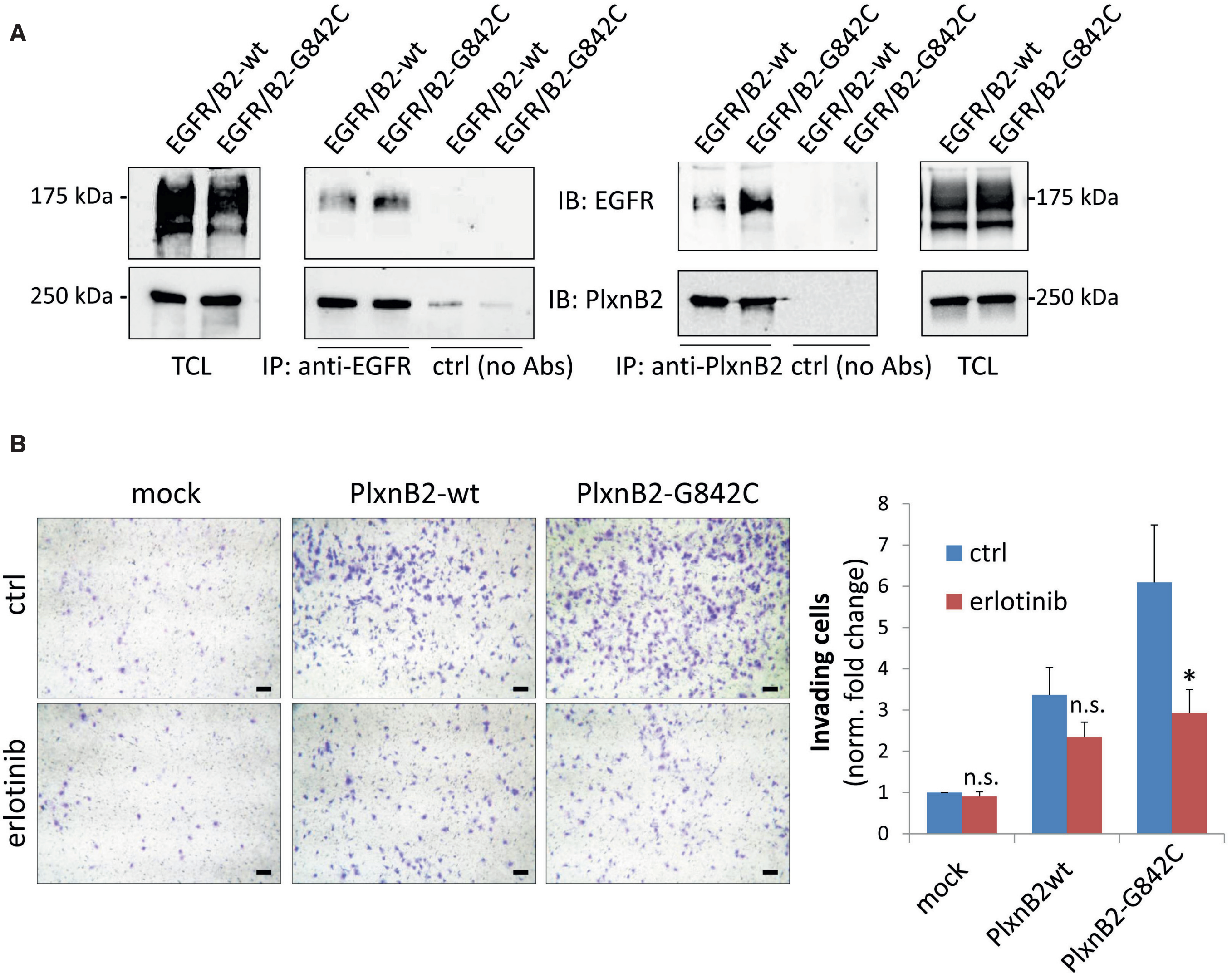

Figure EV5. PlxnB2-EGFR complex and EGFR-dependent regulation of CUP cell invasiveness.

A HEK293T cells were co-transfected with EGFR and either wild-type or PlxnB2-G842C. On the left, total cell lysates (TCL) were subjected (or not) to immunoprecipitation with anti-EGFR, and immunoblotted with the indicated antibodies. Data shown are representative of $n = 3$ experiments. On the right, EGFR-PlxnB2 co-immunoprecipitation was reciprocally assessed in HEK293T cells co-transfected as above, by subjecting total cell lysates to immunoprecipitation with anti-PlxnB2 antibodies. Data shown are representative of $n = 2$ experiments.

B Similar to the experiment shown in main Fig 9D, the invasiveness of AS906 cells either mock, or overexpressing WT or PlxnB2-G842C, was assessed in matrigel-coated Transwell inserts, in the presence or absence of the EGFR inhibitor erlotinib 1 μM. Invading cells were stained with crystal violet, photographed (see representative low magnification images on the left; scale bar: 100 μm), and quantified by ImageJ. Plotted values are the mean ± SD of $n = 3$ independent experiments. The statistical significance across replicates was verified by unpaired t-test multiple comparisons between erlotinib-treated and untreated conditions, per each group: $^*P = 0.021$.

© 2023 The Authors
