# Supplementary figures and images for: Mutated axon guidance gene PLXNB2 sustains growth and invasiveness of stem cells isolated from cancers of unknown primary
Source: EMBO Mol Med. 2023 Feb 1;15(3):e16104. doi: 10.15252/emmm.202216104 (PMC9994481; doi:10.15252/emmm.202216104)

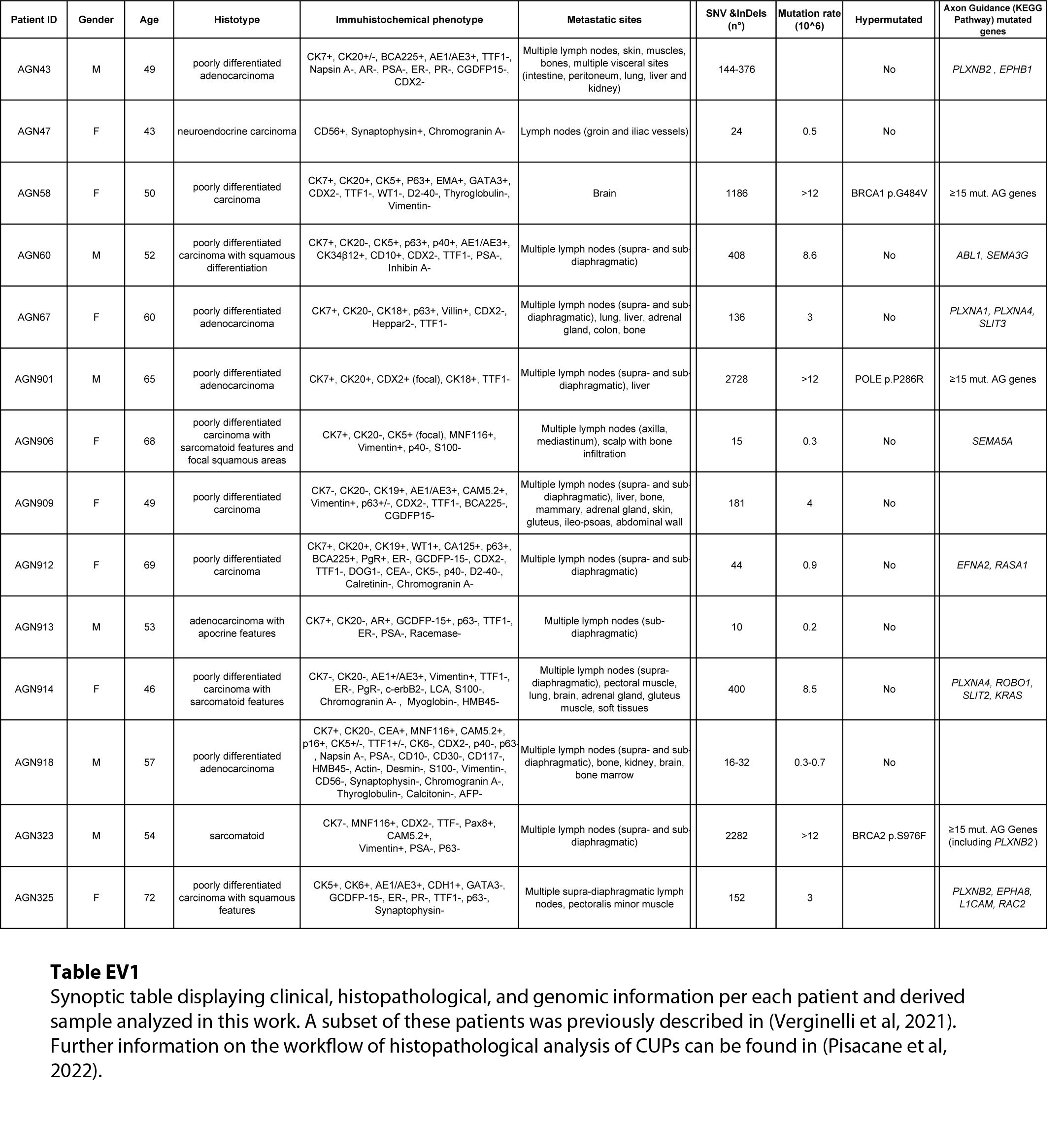

Supplement: Supplementary file 3 — Table EV1 [file EMMM-15-e16104-s007.jpg]

**Fig.7A**

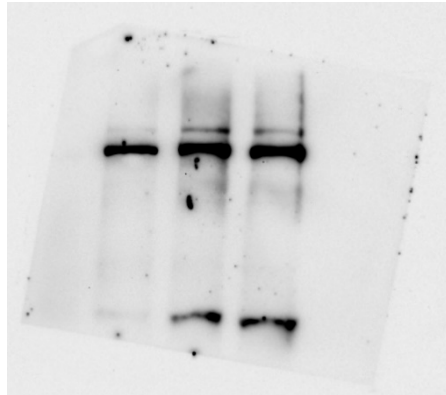

Plexin B2

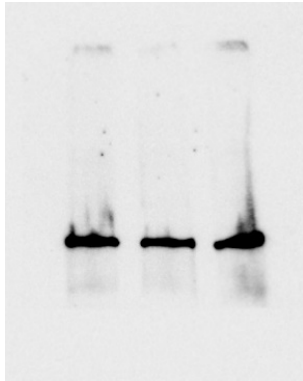

Vinculin

**Fig.7D**

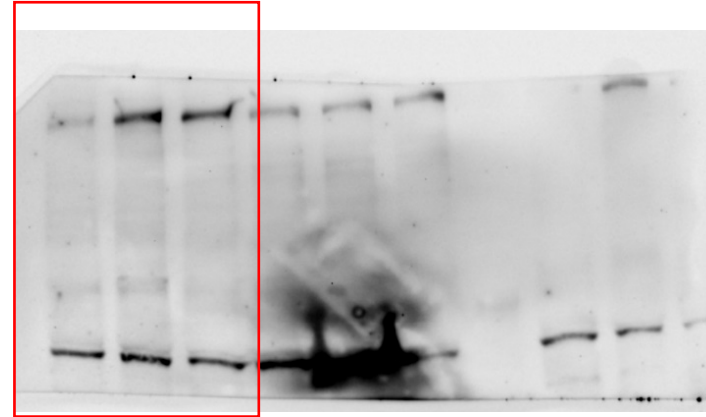

Plexin B2

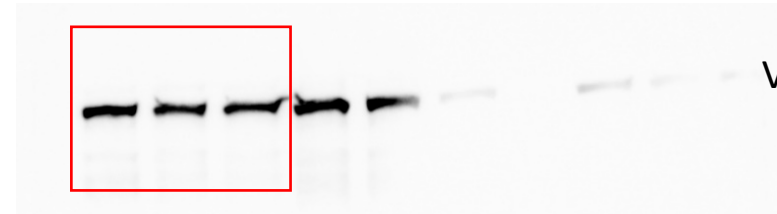

Vinculin

Supplement: Supplementary file 9 — Source Data for Figure 7 [file EMMM-15-e16104-s006.zip › Fig._7_raw_data_WB.pdf]
