# Supplementary material for: Mutated axon guidance gene PLXNB2 sustains growth and invasiveness of stem cells isolated from cancers of unknown primary
Source: EMBO Mol Med. 2023 Feb 1;15(3):e16104. doi: 10.15252/emmm.202216104 (PMC9994481; doi:10.15252/emmm.202216104)
Supplement: Supplementary file 5 — PDF+ [file EMMM-15-e16104-s003.pdf]

Article

SOURCE DATA TRANSPARENT PROCESS OPEN ACCESS

EMBO Molecular Medicine

Mutated axon guidance gene *PLXNB2* sustains growth and invasiveness of stem cells isolated from cancers of unknown primary

Serena Brundu[1,†], Virginia Napolitano[2,†], Giulia Franzolin[1,†], Ettore Lo Cascio[3], Roberta Mastrantonio[2], Gabriele Sardo[1], Eliano Cascardi[1,4], Federica Verginelli[1], Sergio Sarnataro[5], Gennaro Gambardella[5,6], Alberto Pisacane[1], Alessandro Arcovito[3,7], Carla Boccaccio[1,8], Paolo M Comoglio[9], Enrico Giraudo[1,10,*,‡] & Luca Tamagnone[2,7,*,‡]

Abstract

The genetic changes sustaining the development of cancers of unknown primary (CUP) remain elusive. The whole-exome genomic profiling of 14 rigorously selected CUP samples did not reveal specific recurring mutation in known driver genes. However, by comparing the mutational landscape of CUPs with that of most other human tumor types, it emerged a consistent enrichment of changes in genes belonging to the axon guidance KEGG pathway. In particular, G842C mutation of PlexinB2 (PlxnB2) was predicted to be activating. Indeed, knocking down the mutated, but not the wild-type, PlxnB2 in CUP stem cells resulted in the impairment of self-renewal and proliferation in culture, as well as tumorigenic capacity in mice. Conversely, the genetic transfer of G842C-PlxnB2 was sufficient to promote CUP stem cell proliferation and tumorigenesis in mice. Notably, G842C-PlxnB2 expression in CUP cells was associated with basal EGFR phosphorylation, and EGFR blockade impaired the viability of CUP cells reliant on the mutated receptor. Moreover, the mutated PlxnB2 elicited CUP cell invasiveness, blocked by EGFR inhibitor treatment. In sum, we found that a novel activating mutation of the axon guidance gene *PLXNB2* sustains proliferative autonomy and confers invasive properties to stem cells isolated from cancers of unknown primary, in EGFR-dependent manner.

Keywords CUP; EGFR; exome; mutation; Plexin

Subject Categories Cancer; Chromatin, Transcription & Genomics; Molecular Biology of Disease

DOI 10.15252/emmm.202216104 | Received 31 March 2022 | Revised 28 December 2022 | Accepted 11 January 2023 | Published online 1 February 2023

EMBO Mol Med (2023) 15: e16104

Introduction

Cancer of unknown primary (CUP) is a heterogeneous clinical syndrome and pathological entity represented by metastatic tumors that are first time diagnosed in the absence of a clinically detectable primary lesion (Fizazi *et al*, 2015; Rassy & Pavlidis, 2020). CUPs account for about 2–5% of all cancer diagnoses and are characterized by a rapidly progressing clinical course and short median survival (< 1 year) (Pavlidis & Pentheroudakis, 2012; Olivier *et al*, 2021). Thus far, although high throughput sequencing has been applied to CUP samples, their genetic landscape remains poorly understood, and the molecular mechanisms underpinning the early metastatic dissemination still await elucidation. In most recent years, CUPs were subjected to genomic surveys focused on panels of genes considered oncogenic drivers and selected according to their relevance as potential therapeutic targets (Ross *et al*, 2015, 2021; Löffler *et al*, 2016; Subbiah *et al*, 2017; Varghese *et al*, 2017; Zehir *et al*, 2017; Benvenuti *et al*, 2020).

1 Candiolo Cancer Institute, FPO-IRCCS, Turin, Italy
2 Department of Life Sciences and Public Health, Università Cattolica del Sacro Cuore, Rome, Italy
3 Department of Biotechnological Sciences and Intensive Care, Università Cattolica del Sacro Cuore, Rome, Italy
4 Department of Medical Sciences, University of Turin, Turin, Italy
5 Telethon Institute of Genetic and Medicine, Pozzuoli, Italy
6 Department of Electrical Engineering and Information Technology, University of Naples Federico II, Naples, Italy
7 Fondazione Policlinico Gemelli (FPG) – IRCCS, Rome, Italy
8 Department of Oncology, University of Turin, Turin, Italy
9 IFOM, FIRC Institute of Molecular Oncology, Milan, Italy
10 Department of Science and Drug Technology, University of Turin, Turin, Italy
*Corresponding author. Tel: +39 011 9933279; E-mail: enrico.giraudo@ircc.it
**Corresponding author. Tel: +39 063 0154463; E-mail: luca.tamagnone@unicatt.it
[†]These authors contributed equally to this work
[‡]These authors contributed equally to this work as co-senior authors

© 2023 The Authors. Published under the terms of the CC BY 4.0 license.

In all studies, the most recurrent and biologically relevant alterations observed were those affecting TP53 (about 50% of the samples), while CUP-specific genetic changes did not emerge.

Notably, beyond the impact of individual mutations, the genetic landscape of other cancer types revealed the enrichment of diverse alterations in components of the same functional pathway. For example, genomic analyses revealed changes in axon guidance pathway genes in highly metastatic tumors such as pancreatic adenocarcinoma (Biankin *et al*, 2012) and neuroblastoma (Li *et al*, 2017). Indeed, axon guidance cues, beyond their role in the wiring of the neural network, are known to control a wide range of cell migration processes and are increasingly implicated in human cancer (Chédotal *et al*, 2005). For example, neural crest cells disseminate throughout the organism in response to specific attractive and repelling cues encoded by genes in this family (e.g., SDF1/CXCL12, Neuregulin, GDNF/Artemin, and Sema3A/3F). Other signals controlling both axonal navigation and cell migration include hepatocyte growth factor, and large families of guidance cues conserved in evolution: Semaphorins, Ephrins, Netrins, and Slits (Hinck, 2004). In fact, the specific receptors for these signals are expressed on the surface of migrating cells and extending axons. In particular, semaphorins form the largest family of guidance cues, either secreted or membrane bound, and their specific transmembrane receptors are represented by the Plexins, counting nine members in humans (Yazdani & Terman, 2006; Neufeld *et al*, 2016). This complex and versatile vocabulary of signaling molecules is widely used in development, while most guidance cues are downregulated in the adult stage; yet, their functional relevance resumes in case of tissue remodeling and regeneration, as well as in cancer (Chédotal *et al*, 2005; Fard & Tamagnone, 2021).

In the present work, we aimed at characterizing the genetic landscape of the CUP syndrome, undertaking for the first time a whole-exome analysis on a cohort of unambiguously selected CUP patients, in order to discover any genetic dependencies and functional vulnerabilities. In addition, we explored the potential enrichment, in CUPs, of mutations affecting specific signaling pathways; this revealed a putative functional role of axon guidance genes, and of the mutated semaphorin receptor *PLXNB2* in particular. Notably, previous studies have implicated this axon guidance receptor in the regulation of cancer cell proliferation, invasiveness, and metastatic spreading (Le *et al*, 2015; Yu *et al*, 2017; Gurrapu *et al*, 2018; Huang *et al*, 2021); however, nothing was known about *PLXNB2* mutations in cancer, or in other settings.

In order to study the functional role of mutated *PLXNB2* in CUP development, we exploited a validated experimental model in culture, that is, cancer stem cell-enriched spheroids derived from CUP biopsies (Verginelli *et al*, 2021), which have been previously demonstrated to faithfully recapitulate the original CUP phenotype and genetic landscape, including *PLXNB2* mutation. Our data indicate that G842C-*PLXNB2* is a novel genetic change enhancing CUP stem cell proliferation, tumorigenic capacity, and EGFR kinase-dependent invasiveness, providing a bona fide proof-of-principle of the functional involvement of mutated axon guidance genes in CUP development.

Results

Whole-exome sequencing of metastasis in a cohort of CUP patients

We undertook a comprehensive genetic analysis of metastases biopsied from 14 CUP patients, unambiguously selected adopting the 2015 ESMO guidelines (Fizazi *et al*, 2015), as described in the Materials and Methods section. Patients included seven women and seven men, with a median age at presentation of 57 (range 42–71) years; one patient had a single metastasis, while the other 13 presented with multiple metastases at diagnosis. The clinicopathological details of these cases are listed in Table EV1 (while Appendix Fig S1 shows representative histological images).

We performed whole-exome sequencing (WES) on DNA extracted from this collection of CUP samples; gDNA from respective patients' PBMCs was used as normal matched control. Genetic alterations have been detected in all samples. However, it was possible to identify three subgroups in the cohort: five tumors may be defined as hypomutated (displaying < 50 genetic alterations per exome), six were normomutated (displaying from 50–250 mutations), and three were hypermutated (with more than 10^6 mutations per sequenced megabase; Campbell *et al*, 2017); the hypermutated status was associated with mutations in either *BRCA1*, *BRCA2*, or *POLE* (see Table EV1).

When ranking genetic changes, based on the frequency in the whole panel of CUPs, the top mutated gene *TP53* was altered in 43% of the samples, consistent with previous evidence in the literature (Tothill *et al*, 2013; Ross *et al*, 2015). The transmembrane transporter ABCA13, the Sushi Domain-Containing Protein 3 precursor CSMD3, the two proteins DNAH2 and DNAH9 involved in ATP hydrolysis and flagella movement, the axon guidance cue PLXNA4, the extracellular matrix protein USH2A, and the two transcription factors ZFHX3 and ZFHX4 were mutated in 36% of the analyzed CUP samples. We furthermore expectedly found frequent mutations in giant genes *TTN* (57% of samples) and *OBSCN* (mutated in 36%), which are at high risk of genetic changes due to random DNA repair errors (Tan *et al*, 2015).

We wondered whether any functional pathways were more frequently hit by genetic changes in CUPs. To answer this question, we applied MEGA-V algorithm (Gambardella *et al*, 2017) to comparatively analyze the status of genes grouped in 186 KEGG pathways (Kanehisa & Goto, 2000), within our panel of CUP samples versus in datasets derived from 33 different cohorts of patients affected by other tumor types (TCGA mutation database; https://gdc.cancer.gov/). Notably, in comparison with nine tumor types with higher mutational load, CUPs did not show a significantly greater number of genetic changes in any of the KEGG functional pathways. However, based on 24 informative tumor comparisons, certain pathways arose as more frequently mutated in CUPs in statistically significant manner (FDR < 0.1; see Dataset EV1). Among top hits, the Axon Guidance pathway (hsa04360; https://www.genome.jp/entry/pathway+hsa04360) caught our attention, due to its emerging role in cancer progression and metastasis (Chédotal *et al*, 2005), and because it included one of the top frequently mutated genes in our CUP cohort, *PLXNA4*. In addition, the axon guidance gene *MET* had been found mutated in a panel of stringently selected CUP cases (Stella *et al*, 2011). Importantly, MEGA-V analysis showed that

 © 2023 The Authors

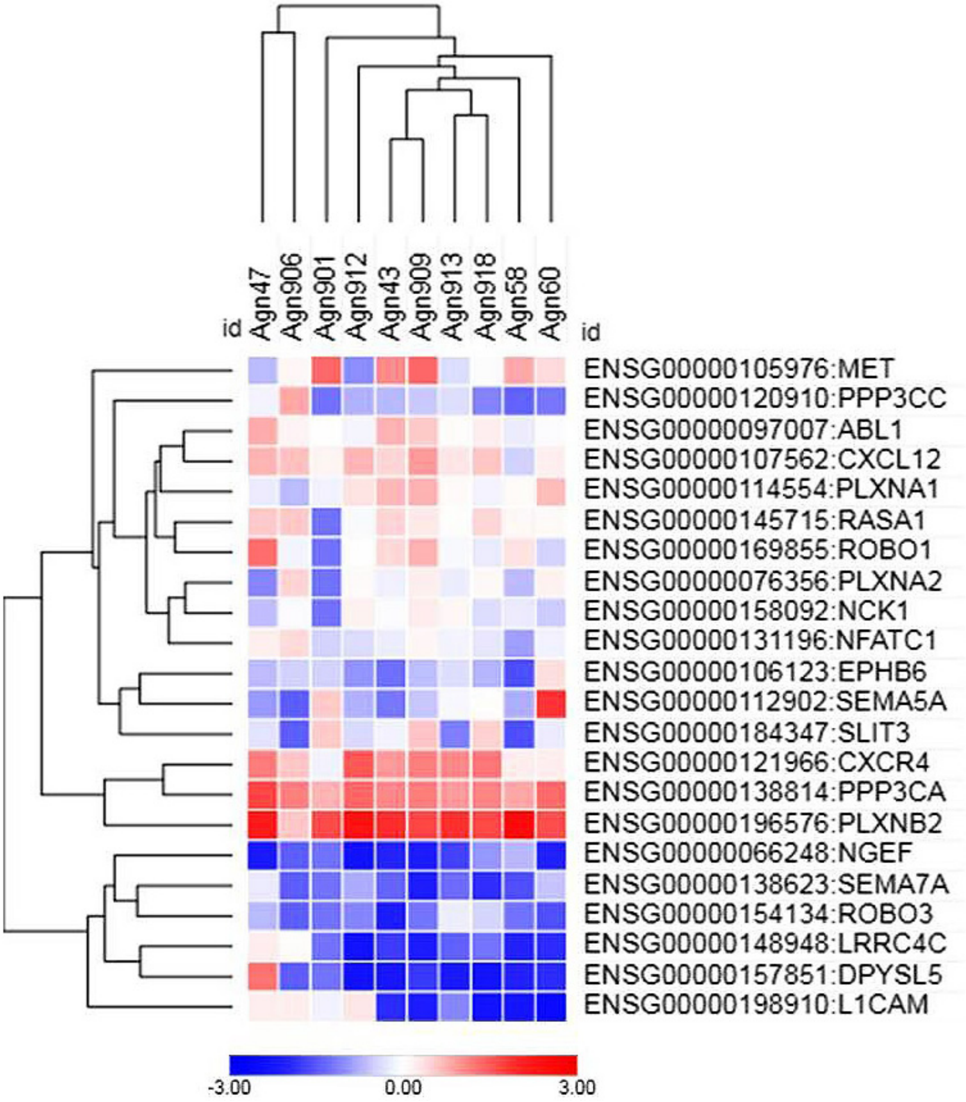

Figure 1. Heatmap representing a hierarchical clustering analysis of the expression profiles of axon guidance genes mutated in the CUP cohort.

By analyzing the transcriptomic profile of each tumor sample, z-score values were calculated, as log2(cpm + 1). Mutated genes belonging to the Axon Guidance pathway (as annotated in KEGG pathway database) were then extracted and hierarchical clustering analysis was performed using Morpheus tool (https://software.broadinstitute.org/morpheus/), with the following setting parameters: Metric: Euclidean distance; Linkage method: Average.

mutations in axon guidance pathway genes were enriched in CUPs versus each of the 24 comparable tumor types and never underrepresented; and this difference was highly statistically significant (with FDR < 0.1 in all cases, but one with FDR = 0.103). Moreover, over 70% of the samples in the CUP cohort, independent of histopathological features, actually carried mutations in genes of the axon guidance pathway, with one or often multiple hits, and up to 21 genetic changes in some cases (Table EV1; sequencing data deposited in the European Genome-phenome Archive, under the accession code EGAS00001004868).

In order to tackle the potential functional involvement of these mutated genes in the CUP phenotype, we complemented WES analysis with gene expression profiling. The analysis was performed on 10 out of 14 samples for which RNA was available, and we focused our attention on axon guidance genes found to be mutated in CUPs (Fig 1). Based on Z-score determination in each sample, a small cluster of three genes resulted prominently expressed in all CUPs: *CXCR4*, *PPP3CA* and especially *PLXNB2*, suggesting that their mutated transcripts are likely to be expressed and potentially impact on CUP cell behavior.

A *PLXNB2* mutation found in our CUP cohort, and not reported previously in COSMIC, encoding p.G842C amino acid change, was particularly intriguing based on our structural *in silico* analysis (detailed below). Moreover, this change was found to be conserved in 14 independent metastatic lesions biopsied in the CUP patient AGN43, as well as in the matched patient-derived *ex-vivo* model, called agnospheres (Verginelli *et al*, 2021). Two other PLXNB2 mutations (encoding R531P and L1058S amino acid changes) were

3° IPT domain

**B2MUT**	803	PVITRIQPETGPLGGGIRITILGSNLGVQAGDI-QRISVACRNCSFQPERYSVSTRIVC	860
**B2**	803	PVITRIQPETGPLGGGIRITILGSNLGVQAGDI-QRISVAGRNCSFQPERYSVSTRIVC	860
**B1**	1070	PLIHSVEPLTGPVDGGTRVTIRGSNLGQHVQDVLGMVTVAGVPCAVDAQEYEVSSSLVC	1128
**B3**	856	PSIDAVEPLTGPPEGGLALTILGSNLGRAFADVQYAVSVASRPCNPEPSLYRTSARIVC	916
**A1**	864	PKILKLSPETGPRQGGTRLTITGENLGLRFEDVRLGVRVGKVLCSPVESEYISAEQIVC	922
**A2**	858	PQITEILTVSGPPEGGTRVTIHGVNLGLDFSEIAHHVQVAGVPCTPLPGEYIIAEQIVC	916
**A3**	810	PRITQIHPLVGPKEGGTRVTIVGENLGLLSREVG--LRVAGVRCNSIPAEYISAERIVC	866
**A4**	858	PRITEIIPVTGPREGGTKVTIRGENLGLEFRDIASHVKVAGVECSPLVDGYIPAEQIVC	916
**D1**	891	PEIHAIEPLSGPLDGGTLLTIRGRNLGRRLSDVAHGVWIGGVACEPLPDRYTVSEEIVC	949
**Met**	657	PVITSISPKYGPMAGGTLLTLTG-NYL--NSGNSRHISIGGKTCTLK---SVSNSILEC	709

Figure 2. Multiple alignment of the primary sequences of IPT domains found in Plexin family members and in the homologous Met receptor.

In the first line is reported the G842C-mutated sequence of IPT3 domain of PlexinB2, found in CUP samples. The primary sequences of corresponding IPT3 domains in plexin family members, and in Met receptor tyrosine kinase, are aligned. Nucleotide positions at start/end of the nucleotide stretches are indicated. Highlighted in yellow are highly conserved amino acid residues. Cysteines engaged in disulfide bonds are underlined. The conserved glycine mutated in PlxnB2 is indicated in bold, as well as neighboring cysteines potentially engaged in covalent links.

found in independent CUP samples, from which it was not possible to derive *ex vivo* primary cancer cell models. We therefore decided to focus our study on the potential pathogenic role of this mutant axon guidance receptor in CUP cells.

G842C mutation of PlxnB2 replaces a conserved residue in the extracellular domain, with predicted impact on receptor conformation

PlxnB2 belongs to the plexin family of semaphorin receptors, which comprises nine members in humans. The extracellular moiety of these single-pass transmembrane proteins includes a conserved sema domain, followed by series of cysteine-rich PSI motifs and IPT domains; the latter are characterized by an immunoglobulin-like fold and considered a protein–protein interaction interface. *In silico* analysis of PlxnB2 primary amino acid sequence revealed that Gly842 residue is located in the frame of the conserved third immunoglobulin-like IPT domain. This amino acid site is topologically conserved in six out of nine members of the human plexin family; moreover, it is located upstream of two cysteines (in +3 and +18 position), which are conserved in the corresponding IPT domain of all plexins, and known to be linked by a disulfide intramolecular bond (Fig 2). A similar structure is found in the homologous first IPT domain of Met and Ron tyrosine kinase receptors, which were previously found to be affected by activating mutations in human cancers (Ma *et al*, 2010; Navis *et al*, 2015).

In order to predict the potential damaging impact of G842C amino acid change in PlxnB2, the structure of this protein was retrieved from Alphafold database (Jumper *et al*, 2021; Varadi *et al*, 2022) and three-dimensional model structures of human wild-type plexin and of G842C variant have been generated. Molecular dynamics (MD) simulations were then run to challenge the system. As shown in Fig 3A (focusing on the IPT3 domain), the superimposition of the wild-type protein so obtained (gray structure) with the selected variant (red structure) demonstrates that this mutation does not alter significantly the global fold of PlxnB2 quaternary structure. At the same time, the results of two consecutive MD runs, of 500 ns each, clearly revealed that the mutation caused structural instability of the IPT3 protein domain (Fig 3B and C), as revealed by an increase in root-mean-square deviation of atomic position (RMSD) values with respect to the wild type, along both measured trajectories. Such tertiary structure variation occurs as the substitution of the small glycine residue in this position slackens the loop between βC and βD strands, entailing a greater flexibility on the entire IPT3 domain, and the eventual unfolding of βG strand in the G842C mutant (red structure) with respect to the wild-type protein (gray structure).

Notably, as discussed previously, the variant Cys842 residue is located at a binding distance with both Cys845 and Cys860 residues; therefore, an equilibrium among alternative intrachain disulfide bonds is conceivable. Accordingly, two alternative structures have been analyzed, challenging the formation of disulfide bridges between residues 842–845 (G842C ¦ 842–845) or 842–860 (G842C ¦ 842–860), and two MD simulations of 500 ns each were run for any structure determined. In Fig 3D and E are shown the superimpositions between the wild-type protein structure (gray) and the predicted structure of MD simulations for G842C ¦ 842–845 (blue) or G842C ¦ 842–860 (green), respectively. It is noteworthy that these potential changes in the native fold are not subverting the global structure of PlxnB2, but lead to further destabilization of the tertiary structure of the IPT3 domain, as demonstrated by increased RMSD values compared with both the wild-type and the mutant p.G842C structure shown above (Fig 3F and G). Again, the main culprit is the disruption of the βG strand; but here, due to alternative disulfide bond formation, this is also accompanied by repositioning and partial unfolding of the βD strand, thus enhancing the divergence in BETARMSD score with respect to the wild-type protein (Fig EV1).

In order to validate the relevance of our *in silico* predictions about the impact of G842C mutation, we analogously assessed the consequence of introducing the other PlxnB2 mutations found in

 © 2023 The Authors

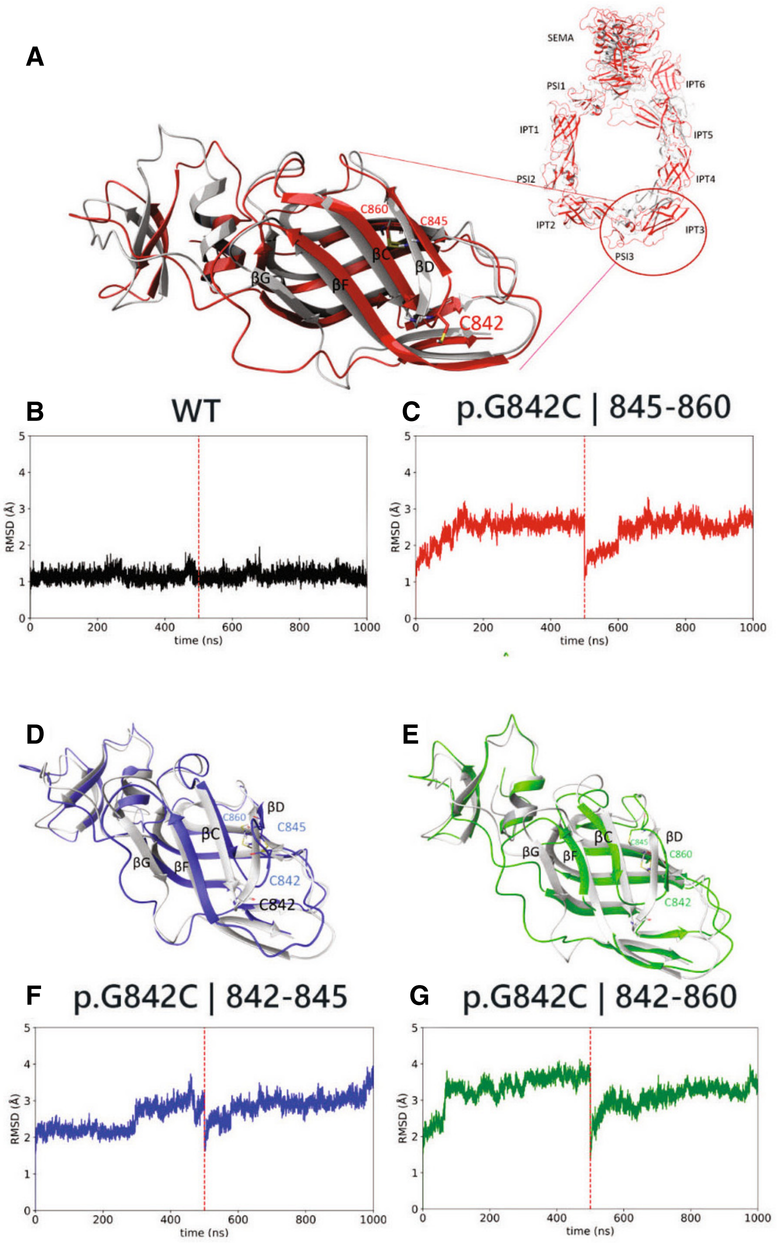

Figure 3. Structural molecular modeling of PlxnB2 wild-type and G842C-mutated form.

A, D, E Most representative conformations of IPT3 domains of PlxnB2 p.G842C (A, in red), PlxnB2 p.G842C 842–845 (D, in blue) and PlxnB2 p.G842C 842–860 (E, in green) compared with WT most representative conformation (in gray).

B, C, F, G RMSD plots of the concatenated replicas of the 4 mentioned IPT3 domains [PlxnB2 WT, p.G482C, p.G842C/842–845, and p.G842C/842–860, respectively].

CUP samples, R531P and P1058S, as well as mutations in the IPT3 domain of PlxnB2 reported in COSMIC and TCGA databases: R820H, L828F, R843Q, and Y852C (see Materials and Methods for data source). This analysis revealed that none of the other mutations had a comparable impact as G842C on the tridimensional conformation of the protein. Notably, simulations for few other mutations falling in the IPT3 domain of PlxnB2 (i.e., R820H, R843Q, and Y852) revealed modest but appreciable alterations of the RMSD plots (Fig EV2).

G842C is a putative gain-of-function mutation leading to PlxnB2 activation in ligand-independent manner

Plexins are well-known receptors for semaphorins, and the prime ligand for PlxnB2 is Sema4C (Deng *et al*, 2007). Notably, semaphorin-induced plexin signaling is often associated with the induction of the so-called "collapsed" phenotype, characterized by retraction of cellular processes and cell rounding; this is a typical *in vitro* response, which is considered a surrogate indicator of plexin

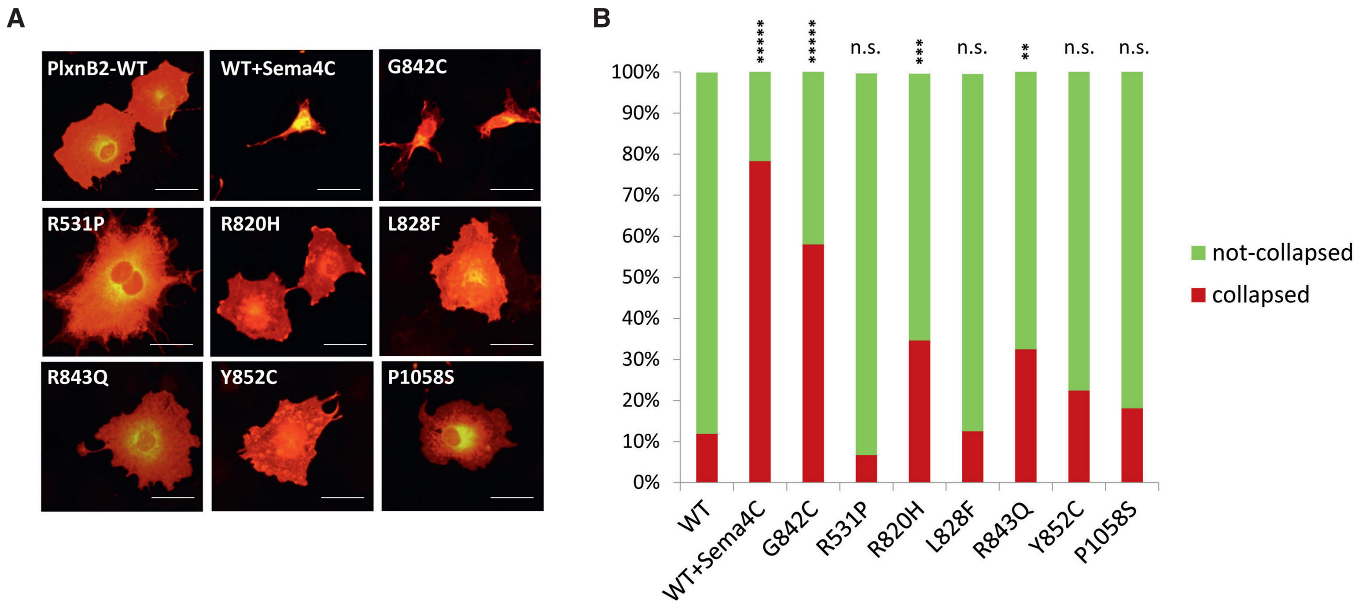

Figure 4. G842C mutation leads to spontaneous activation of PlxnB2 signaling.

A Representative images of immunofluorescence analysis (with anti-PlxnB2 antibodies) of the phenotype of COS7 cells transfected with wild-type or mutated PlxnB2, compared with the collapsed phenotype induced by the cognate ligand Sema4C (1 μg/ml) in cells expressing the WT receptor. Scale bar: 50 μm. A wide series of representative microscopic images is further shown in Appendix Fig S2.

B The stacked bar graph shows the fraction of collapsed PlxnB2-expressing cells, identified as having a surface minor of 2000 μm^2 (measured by ImageJ software), out of 50–100 cells counted per each condition, from different fields and $n > 3$ experiments). The statistical analysis was performed comparing WT untreated cells with all other groups, by Fisher's exact test, which indicated a *****P-value < 0.00001 for Sema4C-treated and for G842C-PlxnB2-expressing cells (confirmed by ANOVA analysis on individual cell values, shown in Appendix Fig S2). By Fisher's test, the only other mutants significantly different from WT were R820H ($P < 0.001$) and R843Q ($P < 0.01$), although ANOVA analysis on individual cell values shown in Appendix Fig S2 did not confirm such significance for other PlxnB2 variants.

Source data are available online for this figure.

activation. Intriguingly, COS7 cells expressing G842C-mutated PlxnB2 displayed a prevalent collapsed phenotype in the absence of the ligand, comparable to that induced by Sema4C in cells bearing the wild-type receptor, which are otherwise mainly spread in basal conditions (Fig 4A and B and Appendix Fig S2). Notably, cells overexpressing the other PlxnB2-IPT3 mutants described above did not show significant changes in the basal phenotype or displayed a modest increase in the number of collapsed cells, significantly inferior to that elicited by G842C mutation discovered in CUPs (Fig 4A and B and Appendix Fig S2). We concluded that G842C-PlxnB2 acts as a putative gain-of-function mutation, leading to ligand-independent receptor activation in overexpressing cells. Noteworthy, although the IPT3 domain of plexins is presumptively not implicated in ligand binding, we also verified whether G842C-PlxnB2 mutation may affect the interaction with Sema4C. Indeed, in a classical *in situ* binding assay with alkaline-conjugated Sema4C, the interaction with the mutated receptor expressed in COS cells appeared unchanged, compared with the wild-type counterpart (Appendix Fig S3).

Knocking down G842C-mutated, but not wild-type PlxnB2, hampers CUP cell viability, clonogenic capacity, and tumorigenic growth in mice

In order to assess the functional relevance of endogenous G842C-mutated PlxnB2 protein, we knocked down its expression in agnospheres AS43, derived from CUP patient AGN43 (validation data in Fig EV3), and evaluated cell proliferation and stem cell frequency. As a comparison, we performed similar experiments in agnospheres AS901, AS906, or AS67 (derived from patients AGN901, AGN906, and AGN67, respectively), which express wild-type PlxnB2. As shown in Fig 5A, PlxnB2 knockdown had no significant impact on the growth of agnospheres carrying wild-type PlxnB2 (compared with controls expressing scrambled shRNAs); in sharp contrast, the depletion of G842C-mutated PlxnB2 in AS43 resulted in a striking inhibition of cell viability and growth versus the respective controls. Similar results were obtained upon PlxnB2 depletion by means of another independent shRNA sequence (Fig EV3).

To assess whether G842C-PlxnB2 could have an impact on cancer cell self-renewal, we performed limiting dilution assays (LDA). By ELDA software analysis, the stem cell frequency (featuring the fraction of clonogenic cells) was estimated in each experimental condition. Consistent with previous observations (Verginelli *et al*, 2021), LDA demonstrated a high clonogenic capacity of AS43 control cells (Fig 5B). Interestingly, the stem cell frequency was strongly reduced in PlxnB2-depleted AS43. By contrast, no significant differences were observed in AS901, AS906, or AS67, upon silencing the wild-type PlxnB2 receptor. Altogether, these data suggest that AS43 cancer cells are specifically dependent on G842C-mutated PlxnB2 for stem properties and proliferation.

Stemming from the results above, to better investigate the role of mutated-PlxnB2 in tumor progression, we generated agnosphere-

 © 2023 The Authors

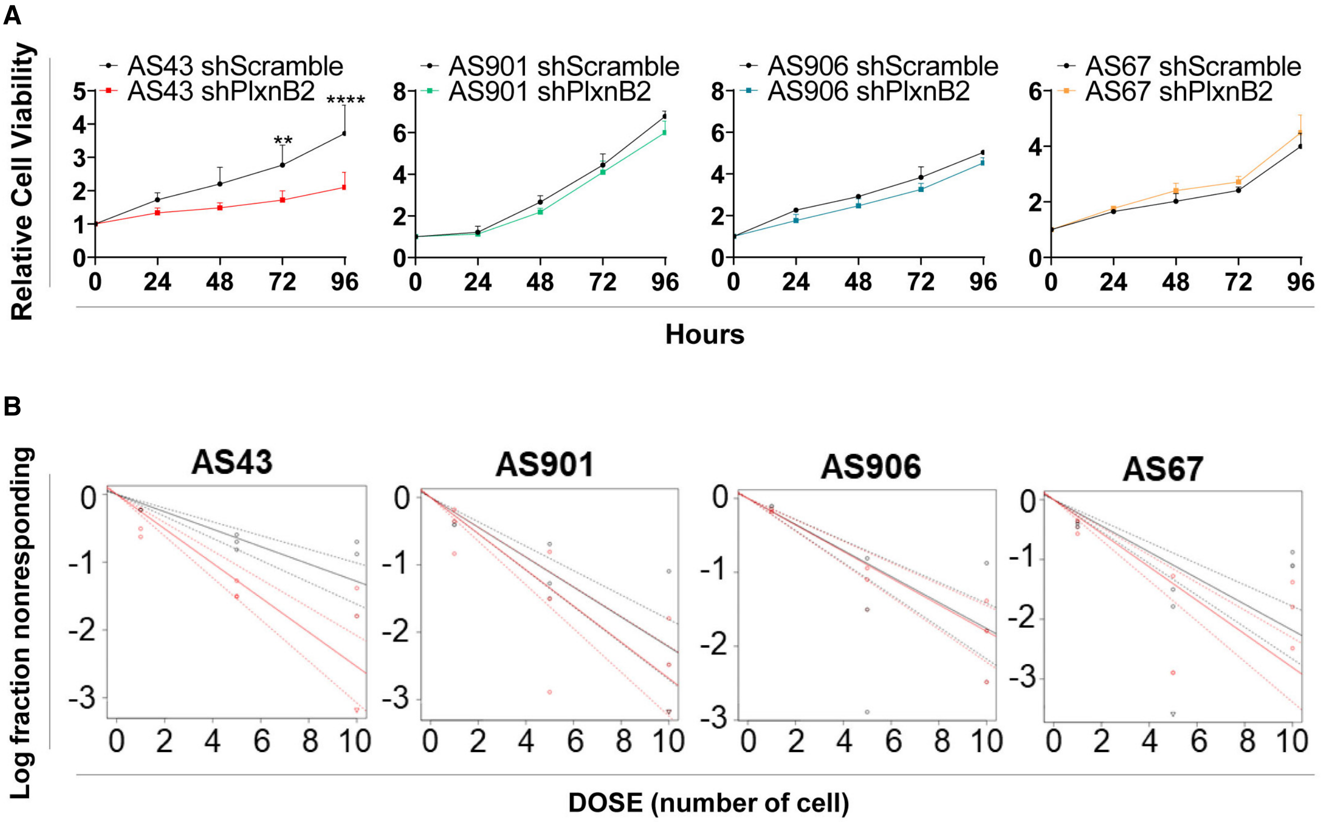

	AS43		AS901		AS906		AS67	
	Stem Cell Frequency	C.I.	Stem Cell Frequency	C.I.	Stem Cell Frequency	C.I.	Stem Cell Frequency	C.I.
**shScramble**	25%	(21%-31%)	27%	(22%-32%)	18%	(15%-22%)	28%	(23%-34%)
**shPlxnB2**	13%	(10%-16%)	22%	(18%-27%)	18%	(14%-22%)	22%	(18%-27%)
	p-value = 0.0000043		p-value = 0.165		p-value = 0.893		p-value = 0.597	

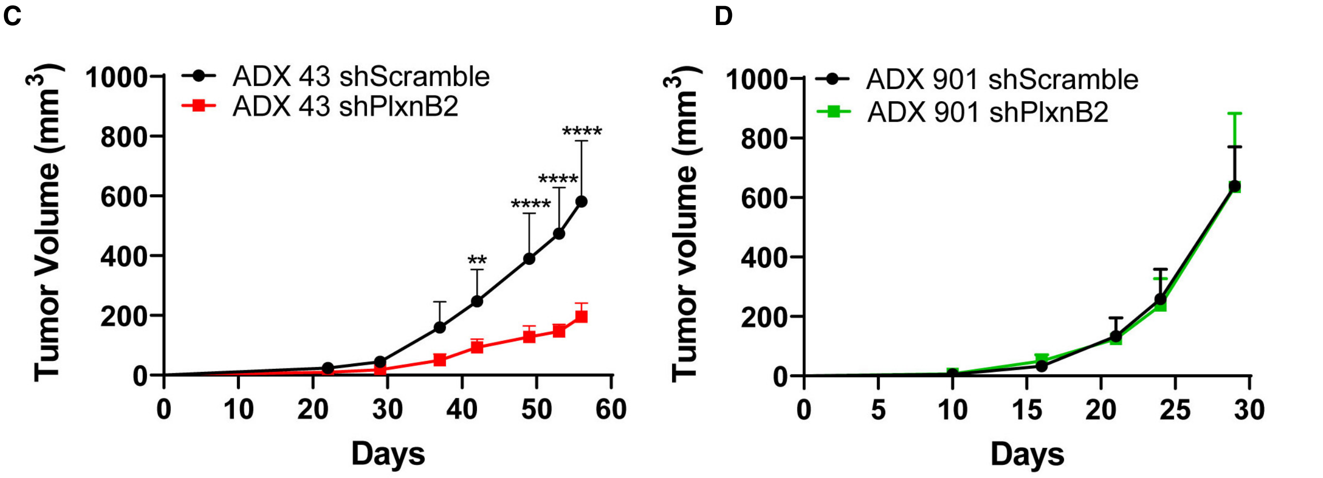

Figure 5.

Figure 5. Knocking down G842C-mutated, but not wild-type PlxnB2, hampers CUP cell viability, clonogenic capacity, and tumorigenic growth in mice.

A The graphs show the analysis of cellular viability in shPlxnB2-tranduced agnospheres, compared with their respective scramble-transfected controls, after 24, 48, 72, and 96 h of growth in culture. Values are mean ± SD of $n \geq 3$ independent experiments (6 technical replicates for each). The statistical significance was assessed by two-way ANOVA with Sidak's correction for multiple comparisons: $^{**}P = 0.0053$; $^{****}P < 0.0001$.

B Limiting dilution assays (LDA, see details in Materials and Methods) were used to assess the clonogenic capacity of dissociated CUP cells derived from the same shPlxnB2-tranduced agnospheres analyzed above, compared with their respective controls. Analyses generated by the ELDA software are shown below, reporting the estimated stem cell frequency (percentage of clonogenic cells) with confidence intervals (C.I.) and statistical analysis by chi-square test.

C, D (C) After transplantation in immunodeficient mice of CUP cells described above, the volume of ADX43- and (D) ADX901-shPlxnB2 tumors was assessed, in comparison with the respective controls. Plotted values indicate the mean ± SD ($n = 8$ each group). The statistical significance was assessed by two-way ANOVA with Sidak's correction for multiple comparisons: $^{**}P = 0.0060$; $^{****}P < 0.0001$.

Source data are available online for this figure.

derived xenograft (ADX) preclinical models, as described previously (Verginelli *et al*, 2021); thus, we injected subcutaneously in NOD-SCID mice either control or PlxnB2-depleted AS43 or AS901 CUP models and monitored tumor growth by periodical calibration. Our results indicated that the expression of G842C-mutated PlxnB2 is specifically required for the growth of AS43-derived tumors. In fact, whereas we observed a marked reduction in tumor volume in PlxnB2-silenced ADX43 mice (Fig 5C), there was no significant difference between silenced and control tumors in ADX901 that carry wild-type PlxnB2 (Fig 5D), further suggesting that G842C-mutated (but not wt) PlxnB2 is promoting CUP-derived ADX growth *in vivo*.

G842C-mutated PlxnB2 enhances CUP cell proliferation and tumor growth *in vivo*

Next, to determine whether the G842C mutation can actually drive tumor cell proliferation, we overexpressed wild-type or mutated PlxnB2 in AS901, AS906, and AS67, which do not basally carry the mutation (validation data in Fig EV3). Interestingly, we found enhanced proliferation of the cells overexpressing mutated PlxnB2 compared with both wt-PlxnB2 and control mock-transduced agnospheres (Fig 6A and Fig EV4).

To further explore the potential of G842C PlxnB2 mutation in a preclinical model, we inoculated NOD/SCID mice with AS901 agnospheres overexpressing mutated or wild-type PlxnB2 (or mock controls). We observed enhanced growth in tumors overexpressing G842C-mutated PlxnB2 compared with both PlxnB2 wt and controls (Fig 6B). Altogether, these data support the conclusion that the mutant G842C-PlxnB2 receptor drives a mechanism promoting the tumorigenic potential of CUP cells.

G842C-mutated PlxnB2 enhances cancer cell migration and invasion

In a previous study, we demonstrated that higher PlxnB2 signaling was associated with increased MCF7 cancer cell migration (Gurrapu *et al*, 2018). These luminal-type breast cancer cells are rather indolent and noninvasive. Thus, in order to assess a potential impact of the G842C mutation in plexin-dependent invasiveness, we initially overexpressed wild-type or mutated-PlxnB2 in MCF-7 cells (Fig 7A). PlxnB2 overexpression, either wild-type or mutated, did not affect the growth rate of MCF7 cells propagated in culture in the presence of serum (unpublished observation, by Serena Brundu). Consistent with previous findings, PlxnB2-WT overexpression promoted basal

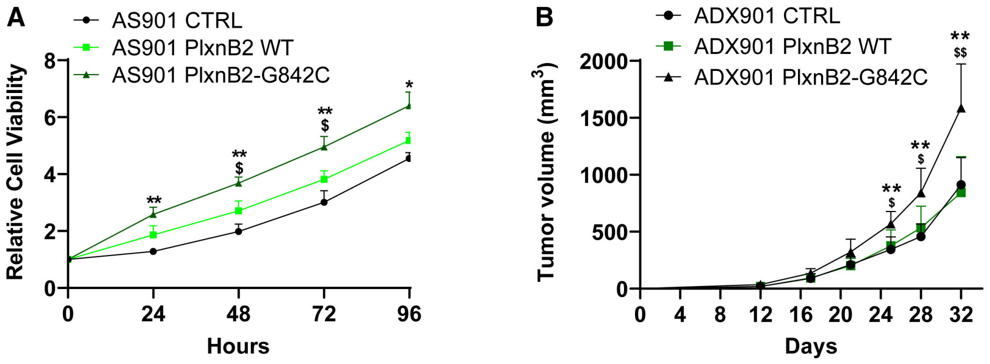

Figure 6. Overexpression of G842C-mutated PlxnB2 in wild-type CUP cells enhanced AS viability and tumor growth in mice.

A Time course analysis of the cellular viability of AS901, stably overexpressing either wild-type or G842C-mutated PlxnB2, or controls transduced with an empty vector. Plotted values are the mean ± SD of $n = 3$ independent experiments (six technical replicates for each). The statistical significance was assessed by two-way ANOVA with Tukey's correction for multiple comparisons. PlxnB2-G842C vs. controls (CTRL): at 24 h $^{**}P = 0.0097$, 48 h $^{**}P = 0.0026$, 72 h $^{**}P = 0.0079$, 96 h $^{*}P = 0.0226$. PlxnB2-G842C vs. PlxnB2-WT: at 48 h $^{\$}P = 0.0418$, 72 h $^{\$}P = 0.0318$.

B Tumor growth curves of ADX 901 overexpressing either PlxnB2-G842C or PlxnB2-WT, or mock control. Plotted values are the mean ± SD ($n = 8$ each group). The statistical significance was assessed by two-way ANOVA with Tukey's correction for multiple comparisons. PlxnB2-G842C vs. control (CTRL): at 25 days $^{**}P = 0.0029$, 28 days $^{**}P = 0.0028$, 32 days $^{**}P = 0.0033$. PlxnB2-G842C vs. PlxnB2-WT: at 25 days $^{\$}P = 0.0221$, 28 days $^{\$}P = 0.0234$, 32 days $^{\$\$}P = 0.0024$.

Source data are available online for this figure.

 © 2023 The Authors

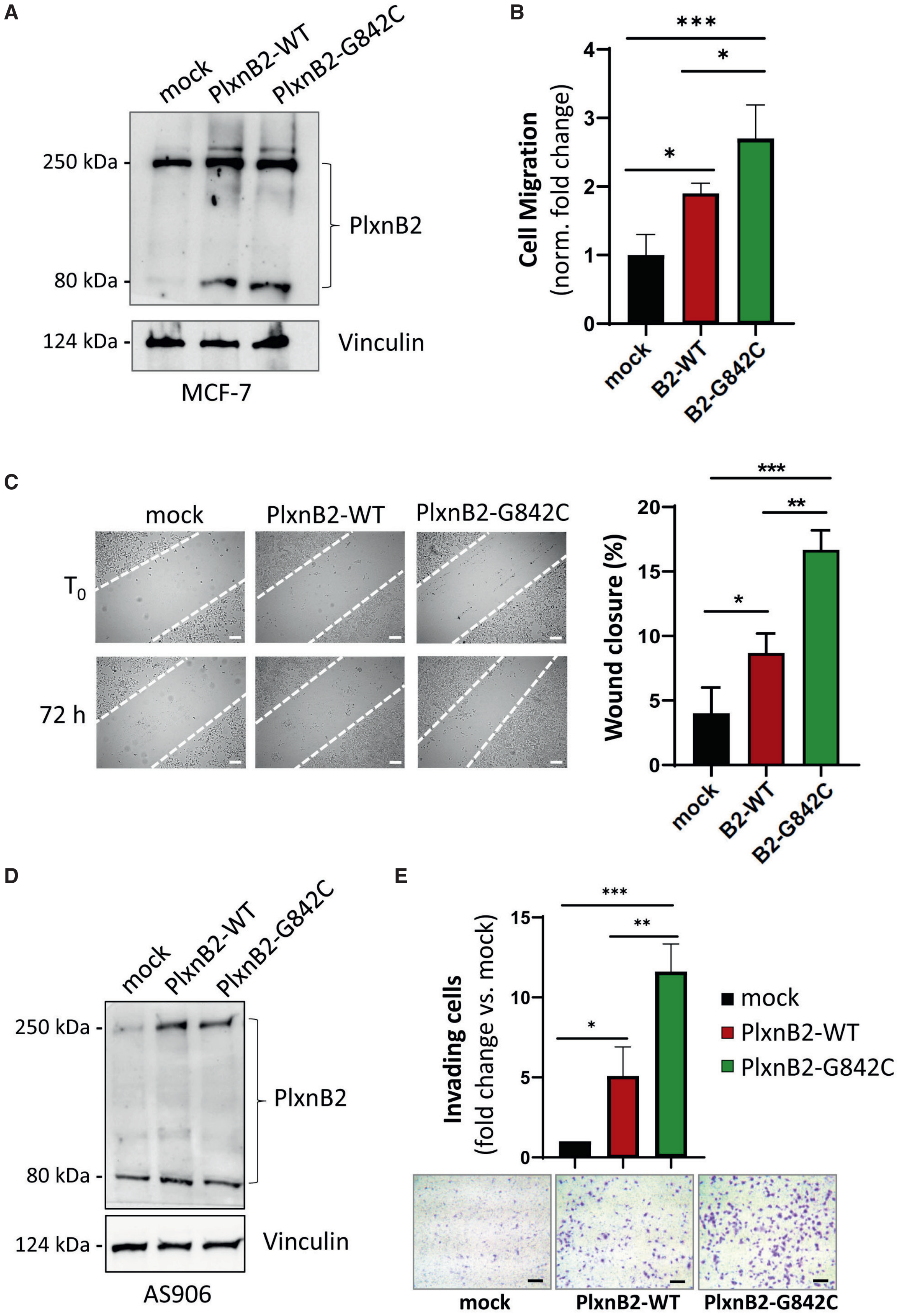
A
mock
PlxnB2-WT
PlxnB2-G842C
250 kDa
80 kDa
PlxnB2
124 kDa
Vinculin
MCF-7
B
Cell Migration
(norm. fold change)
mock
B2-WT
B2-G842C
C
mock
PlxnB2-WT
PlxnB2-G842C
T_0
72 h
Wound closure (%)
mock
B2-WT
B2-G842C
D
mock
PlxnB2-WT
PlxnB2-G842C
250 kDa
80 kDa
PlxnB2
124 kDa
Vinculin
AS906
E
Invading cells
(fold change vs. mock)
mock
PlxnB2-WT
PlxnB2-G842C
mock
PlxnB2-WT
PlxnB2-G842C

Figure 7.

Figure 7. Overexpression of G842C-PlxnB2 promotes cancer cell migration/invasion.

A Western blotting showing basal endogenous or overexpressed PlxnB2, either WT or G842C-mutated isoforms, in MCF7 cells. In this wide view of the hybridized membrane, it is appreciable the presence of full-length molecules at 250 kDa, as well as the processed 80 kDa fragments (reported in Artigiani *et al*, 2003). Vinculin staining provided protein loading controls.

B The graph shows the mean ± SD of relative increase in 24-h spontaneous migration across Transwell inserts of MCF7 cells (analyzed above) overexpressing either wild-type or G842C-mutated PlxnB2, versus mock-transfected controls ($n = 4$ biological replicates). The statistical significance was verified by one-way ANOVA with Bonferroni correction for multiple comparisons: PlxnB2-WT vs. mock *$P = 0.0145$; PlxnB2-G842C vs. PlxnB2-WT *$P = 0.0277$; PlxnB2-G842C vs. mock ***$P = 0.0002$. Representative images of the cells migrated across the semipermeable membrane are shown in Appendix Fig S4A.

C Wound healing scratch assays on monolayers of MCF7 cells engineered as described above. The graph shows the mean ± SD of values obtained in $N = 3$ independent experiments (with at least three technical replicates for each). The statistical significance was verified by one-way ANOVA with Tukey's correction for multiple comparisons: *$P = 0.0350$; **$P = 0.0029$; ***$P = 0.0002$. Representative phase contrast images of the wounded monolayers are further shown in larger size in Appendix Fig S4B.

D Western blotting analysis of endogenous and overexpressed PlxnB2 (either WT or G842C-mutated isoforms) in CUP cells AS906 (both full-length molecules at 250 kDa and processed 80 kDa fragments). Vinculin staining provided protein loading controls.

E The invasive capacity of CUP cells AS906 (shown in previous panel), overexpressing WT or G842-mutated PlxnB2 (or mock control), was assessed across Matrigel-coated Transwell inserts. The graph shows the mean ± SD of values obtained in $N = 3$ independent experiments (including technical duplicates, in two cases). Below the graph, are shown representative fields of the semipermeable membranes (additional larger size images can be found in Appendix Fig S4C); scale bar: 100 µm. The statistical significance was verified by one-way ANOVA with Bonferroni correction for multiple comparisons: *$P = 0.0406$; **$P = 0.0045$; ***$P = 0.0003$.

Source data are available online for this figure.

cancer cell migration to some extent; however, here we found that this effect was far more pronounced in the presence of G842C-mutated receptor (Fig 7B and Appendix Fig S4A). We furthermore analyzed MCF-7 cell migration by scratch assays, in which the gap of a "wounded" monolayer may be filled by migrating cells. Again, wound healing by cells transduced with wild-type PlxnB2 was basally increased compared with controls; however, the expression of G842C-PlxnB2 elicited a strikingly greater wound closure, further suggesting that the mutated plexin was associated with a greater functional activity (Fig 7C and Appendix Fig S4B).

The importance of G842-mutated PlxnB2 in cancer invasion was further assessed in the framework of CUP cells carrying an endogenous wild-type gene. Thus, AS906 transduced to overexpress either wild-type or mutated PlxnB2 (or control cells; Fig 7D) were seeded in the upper chamber of a Transwell insert, on the top of a Matrigel layer, recapitulating the extracellular matrix (ECM). Notably, in the presence of G842-mutated PlexinB2, CUP cells were consistently more efficient than controls in invading the ECM barrier, indicating that this mutation mediates a gain-of-function highly relevant for cancer progression (Fig 7E and Appendix Fig S4C).

G842C-mutated PlxnB2 is required and sufficient to promote EGFR phosphorylation and EGFR-dependent invasiveness in CUP cells

We aimed at identifying signaling mechanisms potentially responsible for the promotion of CUP cell stemness, proliferation, and invasiveness by G842C-mutated PlxnB2. It was previously shown that ligand-dependent PlexinB2 activity elicits ErbB2 tyrosine phosphorylation and oncogenic signaling in breast cancer cells (Gurrapu *et al*, 2018). However, we did not observe a significant change in phospho-ErbB2 levels in association with G842C-mutated PlxnB2 expressed in CUP cells (unpublished observation, by Virginia Napolitano). Intriguingly, in a previous study, we reported that the homologous EGFR tyrosine kinase is basally phosphorylated at low levels in CUP cells carrying a wild-type PlxnB2 (AS901, AS906), while it is highly phosphorylated in AS43 cells, which carry the G842C-mutated receptor (Verginelli *et al*, 2021). EGFR is a major oncogenic promoter in human cancers, especially upon gene amplification and overexpression (Sigismund *et al*, 2018); however, AS43 do not carry *EGFR* mutations or elevated expression, so we posited that the presence of G842C-PlxnB2 might promote its noncanonical activation.

First, we tested the impact of silencing the mutated-PlexinB2 in AS43 CUP cells and found that this resulted in strikingly reduced EGFR tyrosine phosphorylation (Fig 8A), confirming that this altered axon guidance cue controls a major oncogenic pathway in CUP cells. We then analyzed phospho-EGFR levels in basally nonmutated CUP cells (AS906 and AS901), upon transduction of either the wild-type or the G842C-mutated PlxnB2 isoform expressed in AS43. Data in Fig 8B show that the mutated plexin elicited a significantly stronger EGFR phosphorylation, with respect to controls. Thus, our data indicate that G842C-PlxnB2 expression is required and sufficient to sustain EGFR phosphorylation in CUP-derived agnospheres.

Although we were unable to co-immunoprecipitate endogenous EGFR and G842C-PlxnB2 in CUP cells, the two receptors strongly and specifically associated in a complex upon transient transfection in HEK293T cells (Fig EV5A); in this setting, however, we could not achieve conclusive evidence about a quantitative difference between EGFR association with mutated vs. wild-type PlxnB2. Thus, our data suggest that the two receptors can interact in a dynamic complex on the cell surface, which could enable EGFR trans-regulation by mutation-driven conformational changes in PlxnB2.

In order to assess whether the increased basal activation of EGFR in CUP cells carrying mutated PlxnB2 might be accounted to explain their proliferative self-sufficiency, we functionally inhibited EGFR by the specific monoclonal antibody cetuximab, commonly used as anticancer drug. Notably, EGFR blockade in AS43 was associated with abrogated ERK and S6 phosphorylation (Fig 8C), indicating that these intracellular effectors are dependent on EGFR signaling in cells carrying mutated PlexinB2. By contrast, the same pathways were unaffected by cetuximab treatment in CUP cells expressing wild-type PlxnB2 (Fig 8C).

In line with these findings, cetuximab treatment resulted in a substantial inhibition of AS43 cell viability and clonogenic capacity (Fig 9A–C), indicating that these cells are functionally dependent on EGFR signaling. By contrast, AS901, AS906, and AS67, which carry wild-type PlxnB2, were minimally affected or unresponsive to EGFR

 © 2023 The Authors

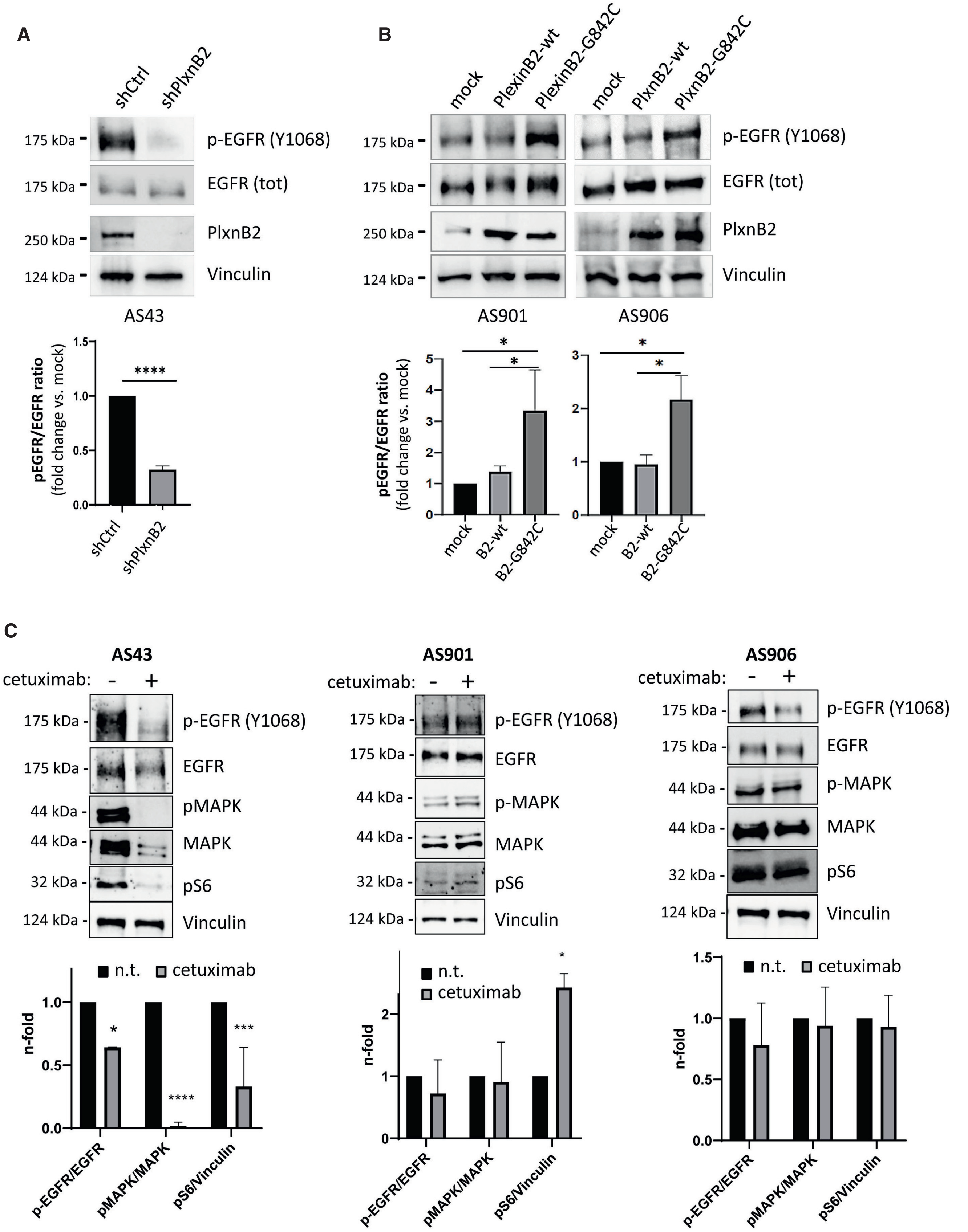
A
shCtrl
shPlxnB2
175 kDa
p-EGFR (Y1068)
175 kDa
EGFR (tot)
250 kDa
PlxnB2
124 kDa
Vinculin
AS43
pEGFR/EGFR ratio (fold change vs. mock)
****
B
mock
PlexinB2-wt
PlexinB2-G842C
mock
PlxnB2-wt
PlxnB2-G842C
175 kDa
p-EGFR (Y1068)
175 kDa
EGFR (tot)
250 kDa
PlxnB2
124 kDa
Vinculin
AS901
AS906
pEGFR/EGFR ratio (fold change vs. mock)
*
*
mock
B2-wt
B2-G842C
C
AS43
AS901
AS906
cetuximab: - +
p-EGFR (Y1068)
EGFR
pMAPK
p-MAPK
MAPK
pS6
Vinculin
175 kDa
44 kDa
32 kDa
124 kDa
n.t.
cetuximab
n-fold
*
****
***
p-EGFR/EGFR
pMAPK/MAPK
pS6/Vinculin

Figure 8.

Figure 8. Expression of G842C-PlxnB2 controls EGFR phosphorylation and intracellular signaling.

A Phospho-EGFR analysis by western blotting in control and PlxnB2-silenced AS43 cells. Images show representative results of $n = 3$ independent experiments, while the graph at the bottom shows the mean ± SD of normalized band quantification. The statistical significance across replicates was verified by unpaired t-test: ****$P < 0.0001$.

B Phospho-EGFR analysis by western blotting in AS901 and AS906 cells either mock, or overexpressing WT or G842C-mutated PlxnB2. Images show representative results of $n = 3$ independent experiments, while the graphs at the bottom show the mean ± SD of normalized band quantification. The statistical significance across independent replicas was verified by one-way ANOVA followed by Tukey test: AS901 PlxnB2-G842C vs. PlxnB2-WT *$P = 0.0434$, PlxnB2-G842C vs. mock *$P = 0.0211$; AS906 PlxnB2-G842C vs. PlxnB2-WT *$P = 0.0436$, PlxnB2-G842C vs. mock *$P = 0.0481$.

C Western blotting analysis of pERK and pS6 intracellular signal transducers in the indicated agnospheres, after 6-h treatment with the EGFR inhibitor cetuximab (50 μg/ml). Images show representative results of $n = 3$ independent experiments, while the graphs at the bottom show the mean ± SD of normalized band quantification. The statistical significance across independent replicas was verified by Sidak's multiple comparison test between cetuximab-treated and untreated conditions, per each group: AS43 *$P = 0.0153$, ***$P = 0.0001$, ****$P < 0.0001$; AS901 *$P = 0.0201$.

Source data are available online for this figure.

blockade (Fig 9A–C). Thus, the increased EGFR activity driven by genetically altered PlxnB2 controls intracellular signaling and proliferative self-sufficiency in CUP cells.

We finally asked whether EGFR kinase activity might be responsible for the increased invasiveness of CUP cells driven by G842C-mutated PlxnB2 overexpression. Notably, cetuximab treatment did not significantly interfere with AS906 cell basal invasion, indicating that this is not dependent on EGFR activity. By contrast, the elevated CUP cells invasiveness elicited by PlexinB2 G842C-mutated isoform was almost completely abrogated by the EGFR inhibitor (Fig 9D), indicating that the axon guidance receptor PlexinB2 is functionally placed upstream EGFR kinase in the regulation of CUP cells. Similar results were obtained upon EGFR blockade with the selective kinase inhibitor erlotinib (Fig EV5B).

Discussion

Surveying the genomic mutational landscape of human cancers has revealed a number of driver genetic changes and associated dependences on activated signaling cascades, enabling the development of specific targeted therapies. This is epitomized by the application of ErbB2-targeted drugs in a subset of breast cancers, of BRAF inhibitors in melanomas, of drugs targeting liabilities due to mutated BRCA in gynecological tumors, to cite some key examples. Notably, the therapeutic targeting of certain relevant mutations is still challenging, such as for K-RAS in colon and pancreatic cancers, but thanks to progresses in research the goal is at sight. Yet, metastasis, the main culprit for cancer-associated lethality, could not be associated so far with specific genetic changes or driver pathways, disabling the chance to design targeted therapeutic approaches, and leaving scanty options for patient treatment at this stage. Paradigmatic examples of this scenario are cancers that appear as primarily metastatic at diagnosis; as such, they also miss the classical indications for tumor type-specific chemotherapy, which leads to the undertaking of agnostic therapeutic approaches with poor efficacy. Actually, unlike most human tumor types, the genomic mutational landscape of cancers of unknown primary (CUP) has been poorly investigated so far, mainly focusing on the hunt for genetic changes in known oncogenes and tumor suppressors.

In the present study, we performed a genome-wide unbiased analysis by NGS of a cohort of CUP samples. A limiting factor to this approach has previously been the lack of a coherent protocol to ensure the recruitment of definite CUP cases, accurately discriminated from early metastatic cancers for which the primary site had not been identified due to incomplete clinicopathological characterization. Thus, the CUP cohort analyzed here was unambiguously selected adopting current ESMO guidelines (Fizazi *et al*, 2015; Pisacane *et al*, 2022). WES analysis of CUP samples did not reveal recurrent genetic changes in known oncogenes or tumor suppressors, apart from frequent hits in TP53, which are in the range of those observed in many other human tumors and can hardly be considered specific. A number of conclusions could be drawn from these findings; obviously, the lack of positive identification of specific

Figure 9. Viability and invasive capacity of CUP cells expressing G842C-mutated, but not wild-type, PlxnB2 is dependent on EGFR.

A The cellular viability of the indicated agnospheres was assessed after 6 days in the presence or absence of the EGFR inhibitor cetuximab (50 μg/ml). In the plot, each box represents values from 25th to 75th percentiles, the central band indicates the mean, and the whiskers expand from maximum to minimum values of individual cetuximab-treated experimental points ($n \geq 9$), normalized to the average value of untreated cells within each group. The statistical significance across three independent experiments, each comprising ≥ 3 technical replicates, was verified by unpaired t-test multiple comparisons of cetuximab-treated and untreated conditions, per each group: ***$P = 0.0002$; ****$P < 0.0001$.

B The viability of AS43, in presence (or absence) of cetuximab (50 μg/ml), was assessed over the indicated time course, compared with values at T_0. The graph shows the mean ± SD of two independent experiments (with quadruplicate technical replicates each). The statistical significance was verified through multiple comparisons by two-way ANOVA with Bonferroni correction of cetuximab-treated and untreated samples, per each time point: ****$P < 0.0001$.

C Limiting dilution assays (LDA) were used to assess the clonogenic capacity of dissociated CUP cells derived from the indicated agnospheres in the presence or absence of cetuximab (50 μg/ml). Analyses generated by the ELDA software are shown below, reporting the estimated stem cell frequency (percentage of clonogenic cells) with confidence intervals (C.I.) and statistical analysis by chi-square test.

D The invasive capacity of AS906 cells (overexpressing WT or G842C-mutated PlxnB2, or mock transfected) was assessed as in Fig 7E, in the presence or absence of cetuximab 50 μg/ml. Images show representative results (scale bar: 100 μm), while the graph on the right shows the mean ± SD of $n = 3$ independent experiments. The statistical significance across replicates was verified by one-way ANOVA with Bonferroni correction: **$P = 0.0010$; ***$P = 0.0005$; ****$P < 0.0001$.

Source data are available online for this figure.

 © 2023 The Authors

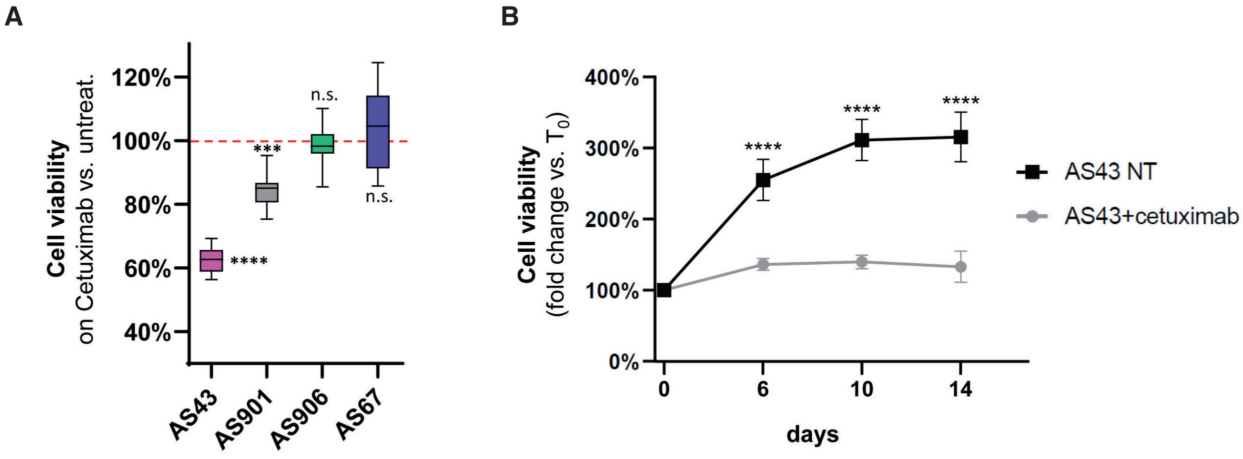

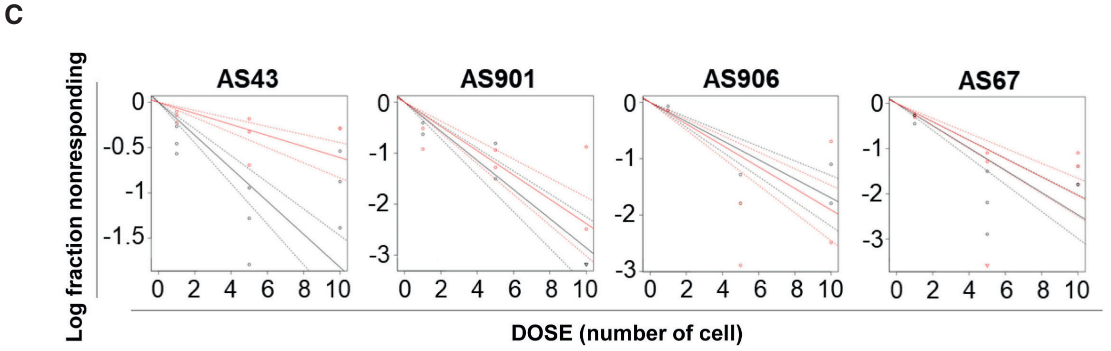

	AS43		AS901		AS906		AS67	
	Stem Cell Frequency	C.I.	Stem Cell Frequency	C.I.	Stem Cell Frequency	C.I.	Stem Cell Frequency	C.I.
AS	18%	(14%-22%)	29%	(23%-36%)	17%	(13%-22%)	25%	(20%-30%)
AS+Cetuximab	6%	(4%-8%)	24%	(19%-30%)	19%	(15%-25%)	20%	(17%-25)
	p-value = 4.19e-10		p-value = 0.26		p-value = 0.523		p-value = 0.174	

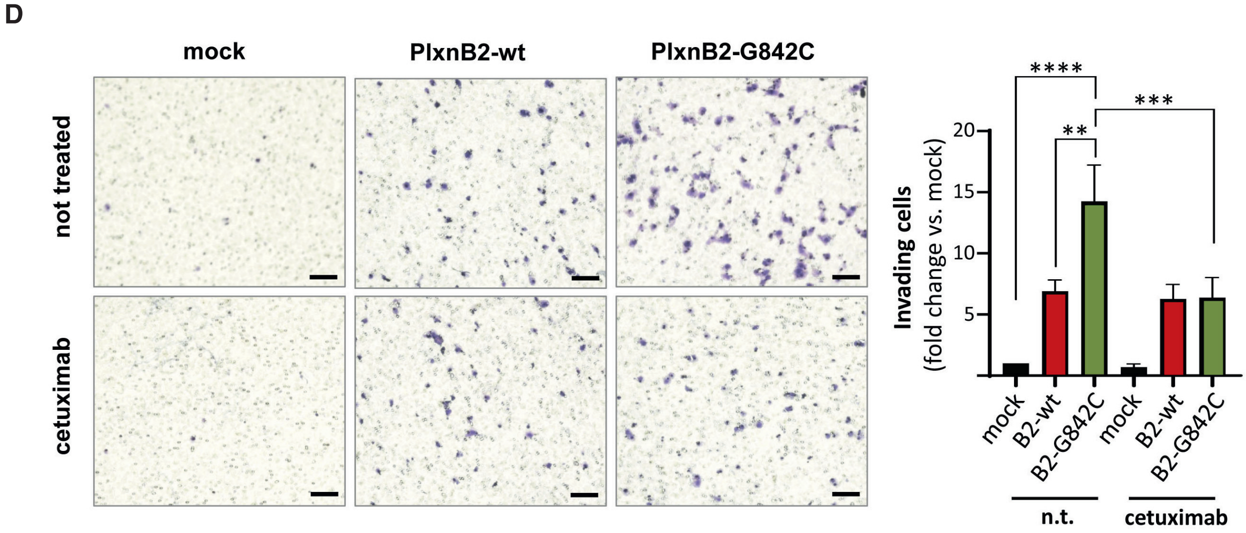

Figure 9.

metastasis-driver mutations may still be accounted by our inability to unveil their presence. However, we posited here that the CUP phenotype may also be sustained by the occurrence of diverse genetic (and possibly epigenetic) changes in some defined pathway controlling cell behavior. In order to tackle this hypothesis, we applied a validated algorithm calculating the frequency of mutations in genes that were associated in functional pathways in the Kyoto Encyclopedia of Genes and Genomes (KEGG). On these bases, the genomic mutational profile of CUPs was compared with those of 33 other tumor types (defined in TCGA database), revealing a significant enrichment of changes in specific pathways. In particular, mutations in axon guidance genes were found in 10 out of 14 CUP samples, independently from clinical or histopathological features of the tumors, and were more frequently occurring than in most other tumor types.

The axon guidance KEGG pathway comprises 125 genes, controlling cell migration and axonal navigation in development, but also associated with pathological dysregulation of cell motility in the adult stage. Notably, many of these genes have been implicated in tumor metastasis, including Met tyrosine kinase, semaphorins, and their receptors, the plexins. We decided to focus our attention on the mutated axon guidance gene most expressed in CUP samples, *PLXNB2*. Although previous studies have implicated this semaphorin receptor in the regulation of cancer cell proliferation, invasiveness, and metastatic spreading (Le *et al*, 2015; Yu *et al*, 2017; Gurrapu *et al*, 2018; Huang *et al*, 2021), none had associated its mutations with a causal role. In order to establish a proof of principle about the relevance of axon guidance genes in CUPs, we investigated G842C-PlxnB2 variant, which was suspected to be damaging based on sequence conservation and structural modeling results. Moreover, this mutation affected the conserved fold of an IPT domain, a moiety also found in Met and Ron oncogenic receptors and previously found to be affected by activating mutations in human cancers (Ma *et al*, 2010; Navis *et al*, 2015). Notably, the large intracellular portion of the plexins does not contain a kinase domain or other classical signaling domains, but was consistently reported to regulate the activity of monomeric GTPases, especially R-Ras, Rap-1, and RhoA, controlling cell migration, and axonal extension in neural development. In addition, plexins have been shown to couple with transmembrane tyrosine kinases of the receptor type such as ErbB2 and Met, thereby triggering alternative noncanonical signaling cascades, especially in cancer cells (Cagnoni & Tamagnone, 2014).

Our experimental evidence indicated that G842C-mutated PlxnB2 was competent for signaling, even in absence of semaphorin stimulation. Interestingly, other studies have recently reported signaling mechanisms mediated by plexins (including PlxnB2), which are not necessarily activated by semaphorins (Mehta *et al*, 2020; Jiang *et al*, 2021; Yang *et al*, 2022). Moreover, while knocking down PlxnB2 expression in CUP cells bearing a wild-type receptor had no apparent functional impact, the metastatic cells carrying G842C mutation proved to be dependent on this variant PlxnB2 to sustain self-renewal and proliferation in culture, as well as tumorigenesis in mice. Altogether, these data indicated that G842C-PlxnB2 can be considered a gain-of-function mutation.

We therefore aimed at assaying the potential self-sufficiency of this mutation to induce a more aggressive cancer phenotype. To this end, the functional impact of ectopically expressed G842C-mutated PlxnB2 was compared to that of the WT receptor, in diverse tumor cells. The variant plexin proved capable to promote cell proliferation and tumor growth, as well as cancer invasiveness, consistent with the selective advantage of its acquisition and maintenance in CUPs. Although initially reported to bind Sema4C with high affinity, PlexinB2 is now considered a relatively promiscuous receptor of class-4 semaphorins, which probably explains its pivotal role in embryo development. In addition to semaphorins, PlexinB2 was reported to act as receptor for the unrelated molecule angiogenin, controlling cancer cell survival and growth, beyond angiogenesis (Yu *et al*, 2017). Moreover, it was recently reported that PlexinB2 acts as a cell surface mechanotransducer, adjusting cell adhesion and migration dynamics to matrix stiffness, and empowering glioblastoma cell invasiveness (Junqueira Alves *et al*, 2021). It is therefore possible to envisage a semaphorin-independent signaling competence of G842C-PlxnB2. Intriguingly, previous studies have reported that a complex conformational mechanism maintains the plexins in the inactive state, since IPT2-*sema* domain intermolecular interaction within dimeric plexin complexes results in receptor auto-inhibition, in the absence of the ligands (Marita *et al*, 2015; Kong *et al*, 2016). Unfortunately, the time course analyzable in our molecular modeling experiments on mutated receptors is limited to 500 ns of simulation, which is insufficient to reveal this kind of large conformational changes. Nevertheless, considering the conservation of the IPT3 domain in the plexin family (Junqueira Alves *et al*, 2019), it may be speculated that a mutation altering the stability of this highly structured domain may impact also on the state of the neighboring IPT2 and possibly destabilize the auto-inhibitory intermolecular complex. Notably, two additional CUP cases in our panel were found to carry *PLXNB2* mutations (summing up to 21% global incidence), though they did not concern Gly842 residue or the IPT3 domain. We analyzed the impact on receptor conformation and activity of these other genetic changes, as well as of additional mutations in the PlxnB2 IPT3 domain reported in non-CUP human tumors. However, none of the mutants was found to produce comparable effects as G842C, or in the presence of some mutations the activity seemed to be reduced, altogether supporting the signaling specificity of this novel variant found in CUPs. Future studies will tell if additional genetic variants of this receptor can promote oncogenic signaling in CUPs or other tumor types.

With the aim to elucidate the signaling cascade elicited downstream G842C-PlxnB2 mutant receptor, we posited the involvement of a tyrosine kinase receptor, since members of this superfamily have been previously associated with plexin signaling in cancer cells. Eventually, we identified EGFR as a previously unknown partner of PlxnB2 activity. In fact, PlxnB2 was found in complex with EGFR, and EGFR phosphorylation was enhanced in the presence of G842C-PlxnB2. Moreover, the greater invasiveness of CUP cells driven by the expression of the mutated plexin was abrogated by selective EGFR inhibitors applied in clinical practice, cetuximab and erlotinib.

In conclusion, we found that a novel activating mutation of the semaphorin receptor PlxnB2 sustains the proliferative autonomy of CUP stem cells and enhances their tumorigenic capacity and EGFR-dependent invasiveness. These data provide proof-of-principle of the functional involvement of an unexpected aberrant signaling pathway in CUP development and prompt for the characterization of additional axon guidance genes that we found to be mutated in human CUPs.

 © 2023 The Authors

Materials and Methods

Patient recruitment, diagnosis, and tissue sample collection

Patients were enrolled at Candiolo Cancer Institute within Agnostos Profiling protocol (no. 010-IRCC-10IIS-15) approved by the Institute Ethical Committee. Informed consensus was obtained from all patients and the experiments conformed to the principles set out in the WMA Declaration of Helsinki and the Department of Health and Human Services Belmont Report. The diagnostic workflow of CUPs followed ESMO guidelines (Fizazi *et al*, 2015). Fresh human specimens were collected and either stored in RNAlater (Life Technologies) or fixed in 4% buffered formaldehyde and embedded in paraffin. Sections were stained with hematoxylin and eosin to select tumor cell-rich areas before RNA extraction.

RNA extraction, libraries preparation, and sequencing

Total RNA from FFPE from patient's tissues was purified using Maxwell® RSC RNA FFPE Kit (Promega). RNA was quantified using the Qubit 2.0 fluorimetric Assay (Thermo Fisher Scientific). Libraries were prepared from 100 ng of total RNA using the QuantSeq 3′ mRNA-Seq Library Prep Kit FWD for Illumina (Lexogen GmbH) and sequenced on a 15 NovaSeq 6000 sequencing system using an S1, 100 cycles flow cell (Illumina Inc.). Fastq files were generated using bcl2fastq (version v2.20.0.422, Illumina Inc.), and trimming was performed with bbduk software (bbmap suite 20 37.31, Joint Genome Institute) and alignment on a human genome reference assembly (hg38) with STAR 2.6.0a (Dobin *et al*, 2013). Expression levels were determined with htseq-count 0.9.1 using cellRanger prebuild genes annotations (Single Cell Gene Expression, 10x Genomics; Ensembl Assembly 93). Data normalization was performed using edgeR (Anders *et al*, 2015).

gDNA extraction, library preparation, and sequencing

Tumor gDNA was isolated using Relia PrepTM gDNA Tissue Miniprep System (Promega). Normal gDNA was derived from peripheral blood mononuclear cells (PBMCs) of the same patient using ReliaPrepTM Blood gDNA Miniprep System (Promega). DNA was quantified using Nanodrop ND1000 spectrophotometer (Thermo Fisher Scientific) and Qubit 4 Fluorometer (Thermo Fisher Scientific). Whole-exome sequencing with 150-bp paired reads was performed with a NextSeq 500 (Illumina) using 1 μg genomic DNA and enrichment for whole exome performed according to SeqCap EZ MedExome (Roche). Adapters were clipped using Scythe (https://github.com/vsbuffalo/scythe) and trimmed using Sickle (https://github.com/najoshi/sickle). Alignment to the human genome (hg38) was done using Burrows–Wheeler Aligner (BWA) MEM (Li & Durbin, 2010). PCR duplicates were removed using rmdup of SAMtools (Li *et al*, 2009). Somatic SNVs and small insertion/deletions (InDels) were identified using Strelka2 (Kim *et al*, 2018). ANNOVAR (Wang *et al*, 2010) was used to annotate nonsilent (nonsynonymous, stopgain, stoploss, frameshift, nonframeshift, and splicing modifications) somatic mutations in each tumor.

MEGA-V analysis

We used MEGA-V (Mutation Enrichment Gene set Analysis of Variants; Gambardella *et al*, 2017) tool to identify gene sets with a significantly higher number of variants in a CUP cohort compared with cohorts of patients affected by other cancer types. To this end, we first used *TCGAbiolinks* (Colaprico *et al*, 2016) package in the R statistical environment to download from TGCA database the simple nucleotide variation datasets of somatic mutations in 33 distinct cancer types (*BRCA*, *AML*, *DLBC*, *CHOL*, *MESO*, *ACC*, *UCS*, *KICH*, *PCPG*, *ESCA*, *THYM*, *TGCT*, *UVM*, *CESC*, *BLCA*, *PAAD*, *LIHC*, *SKCM*, *UCEC*, *PRAD*, *THCA*, *OV*, *LGG*, *SARC*, *COAD*, *READ*, *KIRP*, *GBM*, *STAD*, *LUAD*, *KIRC*, *LUSC*, and *HNSC*), available at: https://gdc.cancer.gov/. We used MEGA-V tool to compare the frequency of mutations in 186 manually curated KEGG gene sets (https://www.genome.jp/kegg/pathway.html; Kanehisa & Goto, 2000) within samples of the CUP cohort, with respect to that observed in each of the 33 other cancer types found in TCGA dataset. Only nonsynonymous point mutations were considered for comparison (i.e., missense, nonsense, and nonstop mutations).

Plexin-B2 systems preparation and molecular dynamics simulations

The human Plexin-B2 structure was retrieved from Alphafold Database (Jumper *et al*, 2021; Varadi *et al*, 2022), available at https://alphafold.ebi.ac.uk/. In the present paper, only the Plexin-B2 extracellular domain was used; therefore, the whole structure was trimmed and the residues with higher confident score (local Distance Difference Test (pLDDT) > 50) have been kept; thus, the system comprehends the amino acidic sequence 21–1,190. In order to set up the p.G842C mutants, firstly Glycine 842 was substituted with a cysteine residue, using Maestro tools. This step was sufficient to generate the pseudo-wild-type mutant where the single point mutation did not perturb the sulfur bridge network, leaving the native bond between aa 845 and 860. Then, the disulfide bond between C845 and C860 was broken, and two different conformations were considered, respectively, between C842 and C845 (for the system p.G842C 842–845) and between C842 and C860 (for the system p.G842C 842–860). Finally, all four systems, the native protein, the pseudo-wild-type one and the two mutants with alternative disulfide bonds, have been minimized with Prime (Jacobson *et al*, 2002, 2004). Classical molecular dynamics simulations were performed with GROMACS 2020.4 package (Abraham *et al*, 2015), using the CHARMM36m force field (Huang *et al*, 2017) at full atomistic level using a TIP3 water solvent. The systems were solvated in a water box of dimension 13.5 × 12.9 × 15.12 Å under periodic boundary conditions. The total charge of the system was neutralized by randomly substituting water molecules with Na^+ ions and Cl^- ions to obtain neutrality with 0.15 M salt concentration. Following a steepest descent minimization algorithm, the system was equilibrated in canonical ensemble (NVT) conditions for 125 ps, using Nose–Hoover thermostat with position restraints for the protein–peptide complexes. Immediately after this minimization procedure, all restraints were removed, and molecular dynamics runs were performed under NPT conditions at 303.15 K, using Nose–Hoover thermostat, with a T-coupling constant of 1 ps, and a Parrinello–Raman barostat at 1 atm. Van der Waals interactions

© 2023 The Authors

were modeled using 6–12 Lennard–Jones potential with a 1.2 nm cutoff. Long-range electrostatic interactions were calculated, with a cutoff for the real space term of 1.2 nm. All covalent bonds were constrained using the LINCS algorithm. The time step employed was 2 fs, and the coordinates were saved every 5 ps for analysis, which was performed using the MDAnalysis (Michaud-Agrawal *et al*, 2011) python library and PLUMED (Bonomi *et al*, 2009; Tribello *et al*, 2014; PLUMED consortium, 2019) tools.

BETARMSD analysis

PLUMED BETARMSD Collective Variable (CV) probes the antiparallel beta-sheet content of protein structures. Two protein segments containing three contiguous residues can form an antiparallel beta sheet. Although, if the two segments are part of the same protein chain, they must be separated by a minimum of two residues allowing enough space for the turn. This CV, thus, generates the set of all possible six residue sections that could form an antiparallel beta sheet and calculates the RMSD distance between the configuration in which the residues find themselves and an idealized antiparallel beta-sheet structure (Pietrucci & Laio, 2009). This is done by calculating the following sum of functions of the RMSD distances:

$$s = \sum \frac{1-\left(\frac{RMSD}{r_0}\right)^n}{1-\left(\frac{RMSD}{r_0}\right)^m}$$

where the sum runs over all possible segments of antiparallel beta sheet. For r_0, the default value of 0.8 was used.

Production of cDNA constructs expressing wild-type and mutated Plexin-B2

Expression constructs for wild-type human Plexin-B2 were reported previously (Gurrapu *et al*, 2018). In a plasmid encoding the full-length receptor, we replaced short cDNA restriction fragments with inserts containing *PLXNB2* mutations, produced through gene synthesis approach (supported by Biocat GmbH, Heidelberg, Germany). In addition to mutations identified in CUP samples in this study, genomic datasets of human non-CUP tumor samples found to carry other PlxnB2 mutations in the IPT3 domain are available at the following links:

R820H: www.cbioportal.org/patient = TCGA-EW-A1IZ; cancer.sanger.ac.uk/cosmic/sample/overview?id = 1766751;
L828F: www.cbioportal.org/patient = TCGA-H4-A2HQ;
R843Q: www.cbioportal.org/patient = TCGA-B5-A3FC;
Y852C: www.cbioportal.org/patient = TCGA-A5-A1OF; cancer.sanger.ac.uk/cosmic/sample/overview?id = 1759420.

Cell culture

Agnospheres were isolated and cultured as described previously (Verginelli *et al*, 2021). Briefly, tumor samples were digested with collagenase I (Gibco), and after filtration, single-cell suspensions were resuspended in culture medium [Dulbecco's Modified Eagle's Medium: F12 (DMEM; Sigma) supplemented with N2 supplement (Life Technologies-GIBCO), BSA 0.5% (Sigma), Heparin 4 μg/ml (Sigma), 2 mM Glutamine (Sigma), and Penicillin–Streptomycin (EuroClone)]. Ultralow-attachment flasks (Corning, cat. CLS3814) were used in case of AS901. The same medium composition was used for further propagation of the agnospheres; when subculturing, agnospheres dissociation was mechanically achieved by pipetting and by trypsin treatment. MCF7 and HEK-293T cells were provided by American Type Culture Collection (ATCC) and cultured in DMEM or Iscove medium (Sigma-Aldrich), respectively, supplemented with 10% fetal bovine serum (FBS; Euroclone), 2 mM Glutamine (Sigma-Aldrich), and Penicillin–Streptomycin (EuroClone). The cells were periodically checked for mycoplasma contamination.

Antibodies and chemicals

Anti-PlexinB2 antibody used for Western blotting analysis was purchased from Abcam (ab193355; dilution 1:500). EGFR antibody was provided by Enzo Life Sciences (ALX-804-064-C100; dil. 1:500), while anti-phospho-EGFR-specific antibodies (directed to p-Tyr1068; ab5644; dil. 1:500) were from Abcam. Anti-vinculin (V4505; dil. 1:1,000) and anti-VSV-G (clone P5D4; dil. 1:1,000) were provided by Sigma-Aldrich. Anti-phospho-p44/42-MAPK (Erk1/2; Thr202/Tyr204; #4370; dil. 1:1,000), anti-p44/42-MAPK (Erk1/2; 137F5; #4695; dil. 1:1,000), and anti-phospho-S6 ribosomal protein (Ser240/244; #2215; dil. 1:1,000) antibodies were from Cell Signaling.

Ligand binding and "collapsed" phenotype analysis by cellular immunostaining

Semaphorin-binding assays were performed as described previously (Tamagnone *et al*, 1999). Briefly, expression constructs encoding WT or mutated PlxnB2 (containing a VSV tag), or a control vector, were transiently transfected in COS-7 cells, by DEAE-Dextran method. Two days after transfection with the receptors, the cells were incubated for 1 h with alkaline phosphatase-conjugated Sema4C; after rinsing, the cells were fixed and the attached ligand was revealed by incubation with NBT/BCIP substrate mix (Promega, cat. S3771).

For immunofluorescence analysis of cell phenotype, transfected COS-7 cells were seeded on glass coverslips; 2 days after transfection, they were fixed in 4% paraformaldehyde for 15 min, permeabilized with 0.1% Triton/phosphate-buffered saline (PBS) for 3 min at room temperature, and then blocked by 5% donkey serum for 30 min. The fixed cells were then incubated with primary antibodies for 1 h at room temperature, followed by incubation with the fluorochrome-conjugated secondary antibodies for 30 min at room temperature. F-actin was stained by using fluorescent-Phalloidin conjugates. Nuclei were stained with 4,6-diamidino-2-phenylindole (DAPI). The coverslips were then washed and mounted on slides. The images were acquired with a confocal laser-scanning microscope (SP5-Leica-CLSM) and analyzed using LAS AF LITE 2.6.0 software (Leica Application Suite); cells with one diameter lower than 40 μm were scored as collapsed.

Lentiviral-mediated shRNA or gene transfer

PLXNB2 knockdown was commonly achieved by lentiviral-mediated transfer of a validated puromycin-selectable construct expressing a targeted shRNA and GFP marker (Origene, cat.

 © 2023 The Authors

TL317033B; targeting seq. 5′-CCACTGGCTGTGGAGCCGAAGCAAG TCCT-3′). For validation experiments, we transferred an independent shRNA sequence carried by TRCN0000048188 clone (targeting seq. 5′-GCTCTACCAATACACGCAGAA-3′), provided by Sigma-Aldrich. For overexpression experiments, a cDNA construct encoding human PlxnB2 (VSV-tagged, provided by Jun Takagi, Osaka, Japan) was subcloned into the lentiviral expression construct pLVX. Moreover, a cDNA fragment containing the sequence encoding PLXNB2-G842C mutation was produced by gene synthesis (BioCat GmbH, Heidelberg, Germany) and replaced to the wild-type sequence, by restriction site-mediated recombination, in the expression construct.

Lentiviral-mediated gene transfer was performed as described previously (Follenzi & Naldini, 2002; Brown *et al*, 2020). Briefly, nonreplicating viral particles containing constructs expressing cDNA or shRNAs (or pGFP-C-shRNA Vector [Origene], as control) were produced in HEK-293 T packaging cells by the calcium phosphate precipitation method. The harvesting of viral particles was carried out 48 h after transfection: the conditioned medium was filtered and centrifuged at 19,500 rpm for 2 h to obtain concentrated viral suspensions. Host cells were then incubated with viral particle-containing media in the presence of 8 μg/ml polybrene at 37° (multiplicity of infection [moi] = 5); CUP cells were dissociated from agnospheres and incubated with viral particles in suspension. Gene-transduced cells were then selected by 0.5 μg/ml puromycin treatment.

Cell viability assay

Agnospheres (400 cells/well) were seeded into 96-well cluster plates (in case of AS901, ultralow-attachment type; Corning, cat. CLS3474). Cell viability was evaluated at 24, 48, and 72 h by CellTiter-Glo® Luminescent Cell Viability Assay (Promega), according to the manufacturer's recommendations by employing the VICTOR X Multilabel Plate Readers (Perkin Elmer).

Limiting dilution assay

CUP cell clonogenic capacity was determined by limiting dilution assays (Hu & Smyth, 2009). Briefly, dissociated cells from different agnospheres were plated into 96-well plates at three different densities, with the following scheme: 30 wells with 1 cell/well, 18 wells with 5 cells/well, and 12 wells with 10 cells/well. After 4 weeks in culture, the number of agnosphere-containing wells was determined by checking under microscope. Positive test was considered wells with spheres larger than 100 μm. Extreme limiting dilution analysis (ELDA) online software was used to calculate the cancer cell stem frequency and statistical significance, by chi-squared test (Hu & Smyth, 2009).

Cell migration and invasion assays

For wound healing assays, MCF7 cells were cultured until 80–90% confluent into 6-well plate with complete medium. Cell monolayers were scratched manually with a micropipette tip; any detached cells were removed by washing with PBS, and the wells were replenished with fresh medium supplemented with 1% FBS. Low magnification images were taken by Leica DM1400B microscope (Leica Microsystems) and then analyzed by ImageJ (NIH, Bethesda, MD, USA) measuring the scratched cell-free area at the time of the scratch (T_0) and after 72 h (T_{72}); wound closure % was computed as 1-(cell-free area at T_{72}/T_0) *100.

Individual MCF-7 cell migration assays were performed using Transwell chamber inserts with a porous polycarbonate membrane (8 μm pore size) (Corning Costar Incorporated, Corning, NY, USA). Briefly, the lower side of the filter was coated with 10 μg/ml fibronectin. 10×10^4 cells were added in the upper chamber in serum-free medium and then allowed to migrate for 24 h through the filter toward the lower chamber with 10% FBS-containing medium. At the end of the experiment, the nonmigrated cells on the upper side of the filter were removed by a cotton swab, followed by fixing with 4% paraformaldehyde, and cell staining with crystal violet. The microscopic images were then quantified either by cell counting or by quantifying the integrated pixel values using ImageJ.

For invasion assays, 1×10^5 AS906 cells were added to the upper chamber of a Transwell insert coated with Matrigel (40 μg; Cultrex Reduced Growth Factor Basement Membrane Extract, Type 2, Pathclear, R&D) and allowed to migrate in the lower chamber. After 48 h, the cells adherent to the lower side of the porous membrane were fixed and analyzed as above.

ADX preclinical model

Agnospheres-derived xenograft preclinical mouse model (ADX) has been described previously (Verginelli *et al*, 2021). Approx. eight-week-old female NOD-SCID mice were purchased from Charles River (Lecco, Italy), and 50,000 cells were injected subcutaneously in each mouse. Once tumor mass became palpable, the tumor burden was measured twice weekly, and the volume was estimated by the formula $(a^2 \times b) \times 0.52$, where a is the minor and b is the major tumor diameter. Mice were sacrificed 4 or 8 weeks after injection, for ADX901 and ADX43, respectively.

All animal procedures were approved by the Ethical Committee of the University of Turin (Candiolo, Turin, Italy) and by the Italian Ministry of Health (auth. n. 741/2020-PR) and were conducted in compliance with European laws and policies. Throughout the study, the mice were kept at a temperature of 22 ± 1°C and a relative humidity of 60 ± 5%, with a 12 h light/dark cycle and 12 air changes/h.

Western blotting analysis and protein immunoprecipitation

The analysis of phosphorylated proteins in CUP cells was done 3 h after cell resuspension at a density of 125,000 cells/ml, in fresh serum-free medium. Cells were lysed in Cell Lysis Buffer (Cell Signaling cat#9803) containing 20 mM Tris–HCl (pH 7.5), 150 mM NaCl, 1 mM Na2EDTA, 1 mM EGTA, 1% Triton, 2.5 mM sodium pyrophosphate, 1 mM beta-glycerophosphate, 1 mM Na3VO4, 1 μg/ml leupeptin, protease inhibitor cocktail (Sigma-Aldrich). Cellular lysates were incubated for 30 min on ice and then centrifuged at 15,000 *g*, 15 min, at 4°C. Protein concentration of cell extracts was determined by using Pierce bicinchoninic acid (BCA) reagent (Thermo Fisher Scientific, cat. 23227) according to the manufacturer's instructions. Protein samples were denaturated by adding a 4× loading buffer (β-mercaptoethanol 0.6 mol/l; SDS 8%; Tris–HCl 0.25 mol/l pH 6,8; glycerol 40%; bromophenol blue 0.2%), incubated at 95°C for 5 min. Samples containing equivalent amounts of

© 2023 The Authors

protein were subjected to 7,5% SDS–PAGE. Proteins were transferred onto a nitrocellulose membrane using the Trans-Blot Turbo Transfer System (Bio-Rad) according to the manufacturer's instructions, probed with Abs of interest, and revealed by enhanced chemiluminescence technique, using Chemidoc Image Lab analyzer and software (Bio-Rad), according to the manufacturer protocols.

For protein immunoprecipitation experiments, HEK-293T cells were transiently transfected to express either PlexinB2-WT or PlexinB2-G842C, in association with EGFR. 48 h after the transient transfection, the cells were transferred on ice, washed three times in cold PBS 1×, and lysed with a buffer containing 20 mM Tris–HCl (pH 7.5), 150 mM NaCl, 1 mM Na2EDTA, 1 mM EGTA, 1% Triton, 2.5 mM sodium pyrophosphate, 1 mM beta-glycerophosphate, 1 mM Na3VO4, 1 μg/ml leupeptin, protease inhibitor cocktail (Sigma-Aldrich). Cellular lysates were incubated for 30 min on ice and then centrifuged at 15,000 *g*, 15 min, at 4°C. The total protein amount was determined using the bicinchoninic acid protein assay reagent (Thermo Fisher Scientific). Equivalent amounts (1 mg) of total proteins were incubated with anti-PlexinB2 antibodies and Dynabeads Protein G (Invitrogen, 10004D), for 4 h at 4°C. Immunocomplexes were washed four times with lysis buffer and then separated by SDS–PAGE. Proteins were finally transferred to a nitrocellulose membrane (Bio-Rad) and analyzed as described previously.

Data analysis and statistics

In general, blinding was not applied, but data analysis was validated by at least two scientists.

Results of experiments in which appropriate negative/positive controls were not validated were consequently excluded from the analysis. The statistical significance of quantitative data was analyzed by GraphPad Prism 8.0.0 software, applying the most appropriate methods and correction tests, specified in individual figure legends. Exact calculated P-values are also indicated in the respective figure legends, apart in case of low-end values: $****P < 0.0001$.

Data availability

Datasets containing WES raw data of CUP samples described in this study are available at the European Genome-Phenome Archive (EGA; https://ega-archive.org/studies/), under the accession codes EGAS00001006621, EGAS00001004868, and EGAS00001004059. Raw data relative to RNA-Seq are available from the Gene Expression Omnibus (GEO) repository, under the accession code GSE167473.

Expanded View for this article is available online.

Acknowledgments

The authors are grateful to Prof. Raffaele Calogero (University of Torino) for advice in gene expression analysis, to Prof. Mattia Lauriola (University of Bologna) for advice in EGFR signaling analysis, and to Silvia Benvenuti, Melissa Milan, and Giorgia Innamorati for help and advice in certain experiments. This work was supported by AIRC—Italian Association for Cancer Research (Molecular Clinical Oncology 5 × 1000 Grant #21052, to P.M.C., C.B., E.G., and L.T.; moreover, V.N. was supported by an AIRC Fellowship for Italy), by the Italian Ministry for Research (PRIN-2017TATYMP grant, to L.T.), and by the Italian Ministry of Health (Ricerca Corrente grants 2022 to E.G. [RRC-2022-23680791]; and to L.T. [FPG-IRCCS]). Università Cattolica del Sacro Cuore also contributed to the funding of this research project and to its publication. This work was furthermore supported by the CINECA supercomputing centers through the grant IsC97 (n.HP10CKTQ6H).

The paper explained

Problem

Cancer of unknown primary (CUP) is a pathological entity represented by metastatic tumors that are first time diagnosed in the absence of clinically detectable primary lesions. The genetic makeup of these tumors, and the mechanisms underlying their accelerated metastatic dissemination, are still elusive. Hence, currently applied therapies are neither specific nor usually effective. Therefore, a better definition of CUP genetic signature, and the identification of new suitable molecular therapeutic targets, may lead to the design of innovative effective treatments, increasing patient survival.

Results

The genomic analysis of confirmed CUP patients revealed a higher frequency of mutations in axon guidance genes, compared with other tumor types. In this study, we particularly focused on a novel activating mutation of PlexinB2 (G842C-PlxnB2), capable of inducing proliferation and invasiveness of CUP stem cells. Notably, the depletion of G842-PlxnB2 resulted in the impairment of cancer cell self-renewal and tumorigenic growth in mice. On the contrary, the overexpression of mutated PlexinB2 enhanced growth and tumorigenic capacity in mice. Remarkably, we found that the sustained proliferative autonomy and invasive properties induced by G842C-PlxnB2 in CUP cells are dependent on the oncogenic tyrosine kinase epidermal growth factor receptor (EGFR) and were abrogated by the treatment with selective EGFR inhibitors validated for clinical use.

Impact

The finding of mutations in axon guidance genes, PLXNB2 in particular, may provide novel genetic biomarkers guiding CUP disease management. Moreover, the identification of EGFR signaling deregulation, as functional consequence of the aberrant activation of axon guidance pathways, may prompt the development of novel therapeutic approaches for CUP patients carrying the implicated mutations.

Author contributions

Serena Brundu: Validation; investigation; visualization; writing—review and editing. **Virginia Napolitano:** Validation; investigation; visualization; writing—review and editing. **Giulia Franzolin:** Validation; investigation; visualization. **Ettore Lo Cascio:** Investigation; visualization. **Roberta Mastrantonio:** Validation; investigation; visualization. **Gabriele Sardo:** Validation; investigation; visualization. **Eliano Cascardi:** Investigation; methodology. **Federica Verginelli:** Resources; validation; investigation; methodology. **Sergio Sarnataro:** Software; validation; investigation; methodology. **Gennaro Gambardella:** Data curation; software; supervision; validation. **Alberto Pisacane:** Data curation; supervision; methodology. **Alessandro Arcovito:** Conceptualization; data curation; software; supervision; validation; writing—review and editing. **Carla Boccaccio:** Conceptualization; resources; data curation; writing—review and editing. **Paolo M Comoglio:** Conceptualization; supervision; funding acquisition; project administration. **Enrico Giraudo:** Conceptualization; supervision; funding acquisition; writing—original draft; project administration; writing—review and editing.

 © 2023 The Authors

Luca Tamagnone: Conceptualization; supervision; funding acquisition; visualization; writing—original draft; project administration; writing—review and editing.

Disclosure and competing interests statement

The authors declare that they have no conflict of interest.

References

Abraham MJ, Murtola T, Schulz R, Páll S, Smith JC, Hess B, Lindahl E (2015) GROMACS: high performance molecular simulations through multi-level parallelism from laptops to supercomputers. *SoftwareX* 1–2: 19–25

Anders S, Pyl PT, Huber W (2015) HTSeq—a python framework to work with high-throughput sequencing data. *Bioinformatics* 31: 166–169

Artigiani S, Barberis D, Fazzari P, Longati P, Angelini P, van de Loo J-W, Comoglio PM, Tamagnone L (2003) Functional regulation of semaphorin receptors by proprotein convertases. *J Biol Chem* 278: 10094–10101

Benvenuti S, Milan M, Geuna E, Pisacane A, Senetta R, Gambardella G, Stella GM, Montemurro F, Sapino A, Boccaccio C *et al* (2020) Cancer of unknown primary (CUP): genetic evidence for a novel nosological entity? A case report. *EMBO Mol Med* 12: e11756

Biankin AV, Waddell N, Kassahn KS, Gingras M-C, Muthuswamy LB, Johns AL, Miller DK, Wilson PJ, Patch A-M, Wu J *et al* (2012) Pancreatic cancer genomes reveal aberrations in axon guidance pathway genes. *Nature* 491: 399–405

Bonomi M, Branduardi D, Bussi G, Camilloni C, Provasi D, Raiteri P, Donadio D, Marinelli F, Pietrucci F, Broglia RA *et al* (2009) PLUMED: a portable plugin for free-energy calculations with molecular dynamics. *Comput Phys Commun* 180: 1961–1972

Brown LY, Dong W, Kantor B (2020) An improved protocol for the production of lentiviral vectors. *STAR Protoc* 1: 100152

Cagnoni G, Tamagnone L (2014) Semaphorin receptors meet receptor tyrosine kinases on the way of tumor progression. *Oncogene* 33: 4795–4802

Campbell BB, Light N, Fabrizio D, Zatzman M, Fuligni F, de Borja R, Davidson S, Edwards M, Elvin JA, Hodel KP *et al* (2017) Comprehensive analysis of hypermutation in human cancer. *Cell* 171: 1042–1056.e10

Chédotal A, Kerjan G, Moreau-Fauvarque C (2005) The brain within the tumor: new roles for axon guidance molecules in cancers. *Cell Death Differ* 12: 1044–1056

Colaprico A, Silva TC, Olsen C, Garofano L, Cava C, Garolini D, Sabedot TS, Malta TM, Pagnotta SM, Castiglioni I *et al* (2016) TCGAbiolinks: an R/Bioconductor package for integrative analysis of TCGA data. *Nucleic Acids Res* 44: e71

Deng S, Hirschberg A, Worzfeld T, Penachioni JY, Korostylev A, Swiercz JM, Vodrazka P, Mauti O, Stoeckli ET, Tamagnone L *et al* (2007) Plexin-B2, but not plexin-B1, critically modulates neuronal migration and patterning of the developing nervous system *in vivo*. *J Neurosci* 27: 6333–6347

Dobin A, Davis CA, Schlesinger F, Drenkow J, Zaleski C, Jha S, Batut P, Chaisson M, Gingeras TR (2013) STAR: Ultrafast universal RNA-seq aligner. *Bioinformatics* 29: 15–21

Fard D, Tamagnone L (2021) Semaphorins in health and disease. *Cytokine Growth Factor Rev* 57: 55–63

Fizazi K, Greco FA, Pavlidis N, Daugaard G, Oien K, Pentheroudakis G, ESMO Guidelines Committee (2015) Cancers of unknown primary site: ESMO clinical practice Guidelines for diagnosis, treatment and follow-up. *Ann Oncol* 26: v133–v138

Follenzi A, Naldini L (2002) HIV-based vectors. Preparation and use. *Methods Mol Med* 69: 259–274

Gambardella G, Cereda M, Benedetti L, Ciccarelli FD (2017) MEGA-V: detection of variant gene sets in patient cohorts. *Bioinformatics* 33: 1248–1249

Gurrapu S, Pupo E, Franzolin G, Lanzetti L, Tamagnone L (2018) Sema4C/PlexinB2 signaling controls breast cancer cell growth, hormonal dependence and tumorigenic potential. *Cell Death Differ* 25: 1259–1275

Hinck L (2004) The versatile roles of «axon guidance» cues in tissue morphogenesis. *Dev Cell* 7: 783–793

Hu Y, Smyth GK (2009) ELDA: extreme limiting dilution analysis for comparing depleted and enriched populations in stem cell and other assays. *J Immunol Methods* 347: 70–78

Huang J, Rauscher S, Nawrocki G, Ran T, Feig M, de Groot BL, Grubmüller H, MacKerell AD (2017) CHARMM36m: an improved force field for folded and intrinsically disordered proteins. *Nat Methods* 14: 71–73

Huang Y, Tejero R, Lee VK, Brusco C, Hannah T, Bertucci TB, Junqueira Alves C, Katsyv I, Kluge M, Foty R *et al* (2021) Plexin-B2 facilitates glioblastoma infiltration by modulating cell biomechanics. *Commun Biol* 4: 145

Jacobson MP, Friesner RA, Xiang Z, Honig B (2002) On the role of the crystal environment in determining protein side-chain conformations. *J Mol Biol* 320: 597–608

Jacobson MP, Pincus DL, Rapp CS, Day TJF, Honig B, Shaw DE, Friesner RA (2004) A hierarchical approach to all-atom protein loop prediction. *Proteins* 55: 351–367

Jiang C, Javed A, Kaiser L, Nava MM, Xu R, Brandt DT, Zhao D, Mayer B, Fernández-Baldovinos J, Zhou L *et al* (2021) Mechanochemical control of epidermal stem cell divisions by B-plexins. *Nat Commun* 12: 1308

Jumper J, Evans R, Pritzel A, Green T, Figurnov M, Ronneberger O, Tunyasuvunakool K, Bates R, Žídek A, Potapenko A *et al* (2021) Highly accurate protein structure prediction with AlphaFold. *Nature* 596: 583–589

Junqueira Alves C, Dariolli R, Haydak J, Kang S, Hannah T, Wiener RJ, DeFronzo S, Tejero R, Gusella GL, Ramakrishnan A *et al* (2021) Plexin-B2 orchestrates collective stem cell dynamics via actomyosin contractility, cytoskeletal tension and adhesion. *Nat Commun* 12: 6019

Junqueira Alves C, Yotoko K, Zou H, Friedel RH (2019) Origin and evolution of plexins, semaphorins, and met receptor tyrosine kinases. *Sci Rep* 9: 1970

Kanehisa M, Goto S (2000) KEGG: Kyoto encyclopedia of genes and genomes. *Nucleic Acids Res* 28: 27–30

Kim S, Scheffler K, Halpern AL, Bekritsky MA, Noh E, Källberg M, Chen X, Kim Y, Beyter D, Krusche P *et al* (2018) Strelka2: fast and accurate calling of germline and somatic variants. *Nat Methods* 15: 591–594

Kong Y, Janssen BJC, Malinauskas T, Vangoor VR, Coles CH, Kaufmann R, Ni T, Gilbert RJC, Padilla-Parra S, Pasterkamp RJ *et al* (2016) Structural basis for plexin activation and regulation. *Neuron* 91: 548–560

Le AP, Huang Y, Pingle SC, Kesari S, Wang H, Yong RL, Zou H, Friedel RH (2015) Plexin-B2 promotes invasive growth of malignant glioma. *Oncotarget* 6: 7293–7304

Li H, Durbin R (2010) Fast and accurate long-read alignment with burrows-wheeler transform. *Bioinformatics (Oxford, England)* 26: 589–595

Li H, Handsaker B, Wysoker A, Fennell T, Ruan J, Homer N, Marth G, Abecasis G, Durbin R, 1000 Genome Project Data Processing Subgroup (2009) The sequence alignment/map format and SAMtools. *Bioinformatics* 25: 2078–2079

Li Y, Ohira M, Zhou Y, Xiong T, Luo W, Yang C, Li X, Gao Z, Zhou R, Nakamura Y *et al* (2017) Genomic analysis-integrated whole-exome sequencing of neuroblastomas identifies genetic mutations in axon guidance pathway. *Oncotarget* 8: 56684–56697

© 2023 The Authors

Löffler H, Pfarr N, Kriegsmann M, Endris V, Hielscher T, Lohneis P, Folprecht G, Stenzinger A, Dietel M, Weichert W *et al* (2016) Molecular driver alterations and their clinical relevance in cancer of unknown primary site. *Oncotarget* 7: 44322–44329

Ma Q, Zhang K, Yao H-P, Zhou Y-Q, Padhye S, Wang M-H (2010) Inhibition of MSP-RON signaling pathway in cancer cells by a novel soluble form of RON comprising the entire sema sequence. *Int J Oncol* 36: 1551–1561

Marita M, Wang Y, Kaliszewski MJ, Skinner KC, Comar WD, Shi X, Dasari P, Zhang X, Smith AW (2015) Class A plexins are organized as preformed inactive dimers on the cell surface. *Biophys J* 109: 1937–1945

Mehta V, Pang K-L, Rozbesky D, Nather K, Keen A, Lachowski D, Kong Y, Karia D, Ameismeier M, Huang J *et al* (2020) The guidance receptor plexin D1 is a mechanosensor in endothelial cells. *Nature* 578: 290–295

Michaud-Agrawal N, Denning EJ, Woolf TB, Beckstein O (2011) MDAnalysis: a toolkit for the analysis of molecular dynamics simulations. *J Comput Chem* 32: 2319–2327

Navis AC, van Lith SAM, van Duijnhoven SMJ, de Pooter M, Yetkin-Arik B, Wesseling P, Hendriks WJAJ, Venselaar H, Timmer M, van Cleef P *et al* (2015) Identification of a novel MET mutation in high-grade glioma resulting in an auto-active intracellular protein. *Acta Neuropathol* 130: 131–144

Neufeld G, Mumblat Y, Smolkin T, Toledano S, Nir-Zvi I, Ziv K, Kessler O (2016) The semaphorins and their receptors as modulators of tumor progression. *Drug Resist Updates* 29: 1–12

Olivier T, Fernandez E, Labidi-Galy I, Dietrich P-Y, Rodriguez-Bravo V, Baciarello G, Fizazi K, Patrikidou A (2021) Redefining cancer of unknown primary: is precision medicine really shifting the paradigm? *Cancer Treat Rev* 97: 102204

Pavlidis N, Pentheroudakis G (2012) Cancer of unknown primary site. *Lancet* 379: 1428–1435

Pietrucci F, Laio A (2009) A collective variable for the efficient exploration of protein Beta-sheet structures: application to SH3 and GB1. *J Chem Theory Comput* 5: 2197–2201

Pisacane A, Cascardi E, Berrino E, Polidori A, Sarotto I, Casorzo L, Panero M, Boccaccio C, Verginelli F, Benvenuti S *et al* (2022) Real-world histopathological approach to malignancy of undefined primary origin (MUO) to diagnose cancers of unknown primary (CUPs). *Virchows Arch* 8: 1–13

PLUMED Consortium (2019) Promoting transparency and reproducibility in enhanced molecular simulations. *Nat Methods* 16: 670–673

Rassy E, Pavlidis N (2020) Progress in refining the clinical management of cancer of unknown primary in the molecular era. *Nat Rev Clin Oncol* 17: 541–554

Ross JS, Sokol ES, Moch H, Mileshkin L, Baciarello G, Losa F, Beringer A, Thomas M, Elvin JA, Ngo N *et al* (2021) Comprehensive genomic profiling of carcinoma of unknown primary origin: retrospective molecular classification considering the CUPISCO study design. *Oncologist* 26: e394–e402

Ross JS, Wang K, Gay L, Otto GA, White E, Iwanik K, Palmer G, Yelensky R, Lipson DM, Chmielecki J *et al* (2015) Comprehensive genomic profiling of carcinoma of unknown primary site: new routes to targeted therapies. *JAMA Oncol* 1: 40–49

Sigismund S, Avanzato D, Lanzetti L (2018) Emerging functions of the EGFR in cancer. *Mol Oncol* 12: 3–20

Stella GM, Benvenuti S, Gramaglia D, Scarpa A, Tomezzoli A, Cassoni P, Senetta R, Venesio T, Pozzi E, Bardelli A *et al* (2011) MET mutations in cancers of unknown primary origin (CUPs). *Hum Mutat* 32: 44–50

Subbiah IM, Tsimberidou A, Subbiah V, Janku F, Roy-Chowdhuri S, Hong DS (2017) Next generation sequencing of carcinoma of unknown primary reveals novel combinatorial strategies in a heterogeneous mutational landscape. *Onco Targets Ther* 4: 47–56

Tamagnone L, Artigiani S, Chen H, He Z, Ming GI, Song H, Chedotal A, Winberg ML, Goodman CS, Poo M *et al* (1999) Plexins are a large family of receptors for transmembrane, secreted, and GPI-anchored semaphorins in vertebrates. *Cell* 99: 71–80

Tan H, Bao J, Zhou X (2015) Genome-wide mutational spectra analysis reveals significant cancer-specific heterogeneity. *Sci Rep* 5: 12566

Tothill RW, Li J, Mileshkin L, Doig K, Siganakis T, Cowin P, Fellowes A, Semple T, Fox S, Byron K *et al* (2013) Massively-parallel sequencing assists the diagnosis and guided treatment of cancers of unknown primary. *J Pathol* 231: 413–423

Tribello GA, Bonomi M, Branduardi D, Camilloni C, Bussi G (2014) PLUMED 2: new feathers for an old bird. *Comput Phys Commun* 185: 604–613

Varadi M, Anyango S, Deshpande M, Nair S, Natassia C, Yordanova G, Yuan D, Stroe O, Wood G, Laydon A *et al* (2022) AlphaFold protein structure database: massively expanding the structural coverage of protein-sequence space with high-accuracy models. *Nucleic Acids Res* 50: D439–D444

Varghese AM, Arora A, Capanu M, Camacho N, Won HH, Zehir A, Gao J, Chakravarty D, Schultz N, Klimstra DS *et al* (2017) Clinical and molecular characterization of patients with cancer of unknown primary in the modern era. *Ann Oncol* 28: 3015–3021

Verginelli F, Pisacane A, Gambardella G, D'Ambrosio A, Candiello E, Ferrio M, Panero M, Casorzo L, Benvenuti S, Cascardi E *et al* (2021) Cancer of unknown primary stem-like cells model multi-organ metastasis and unveil liability to MEK inhibition. *Nat Commun* 12: 2498

Wang K, Li M, Hakonarson H (2010) ANNOVAR: functional annotation of genetic variants from high-throughput sequencing data. *Nucleic Acids Res* 38: e164

Yang H, Yuan L, Ibaragi S, Li S, Shapiro R, Vanli N, Goncalves KA, Yu W, Kishikawa H, Jiang Y *et al* (2022) Angiogenin and plexin-B2 axis promotes glioblastoma progression by enhancing invasion, vascular association, proliferation and survival. *Br J Cancer* 127: 422–435

Yazdani U, Terman JR (2006) The semaphorins. *Genome Biol* 7: 211

Yu W, Goncalves KA, Li S, Kishikawa H, Sun G, Yang H, Vanli N, Wu Y, Jiang Y, Hu MG *et al* (2017) Plexin-B2 mediates physiologic and pathologic functions of angiogenin. *Cell* 171: 849–864.e25

Zehir A, Benayed R, Shah RH, Syed A, Middha S, Kim HR, Srinivasan P, Gao J, Chakravarty D, Devlin SM *et al* (2017) Mutational landscape of metastatic cancer revealed from prospective clinical sequencing of 10,000 patients. *Nat Med* 23: 703–713

License: This is an open access article under the terms of the Creative Commons Attribution License, which permits use, distribution and reproduction in any medium, provided the original work is properly cited.

Expanded View Figures

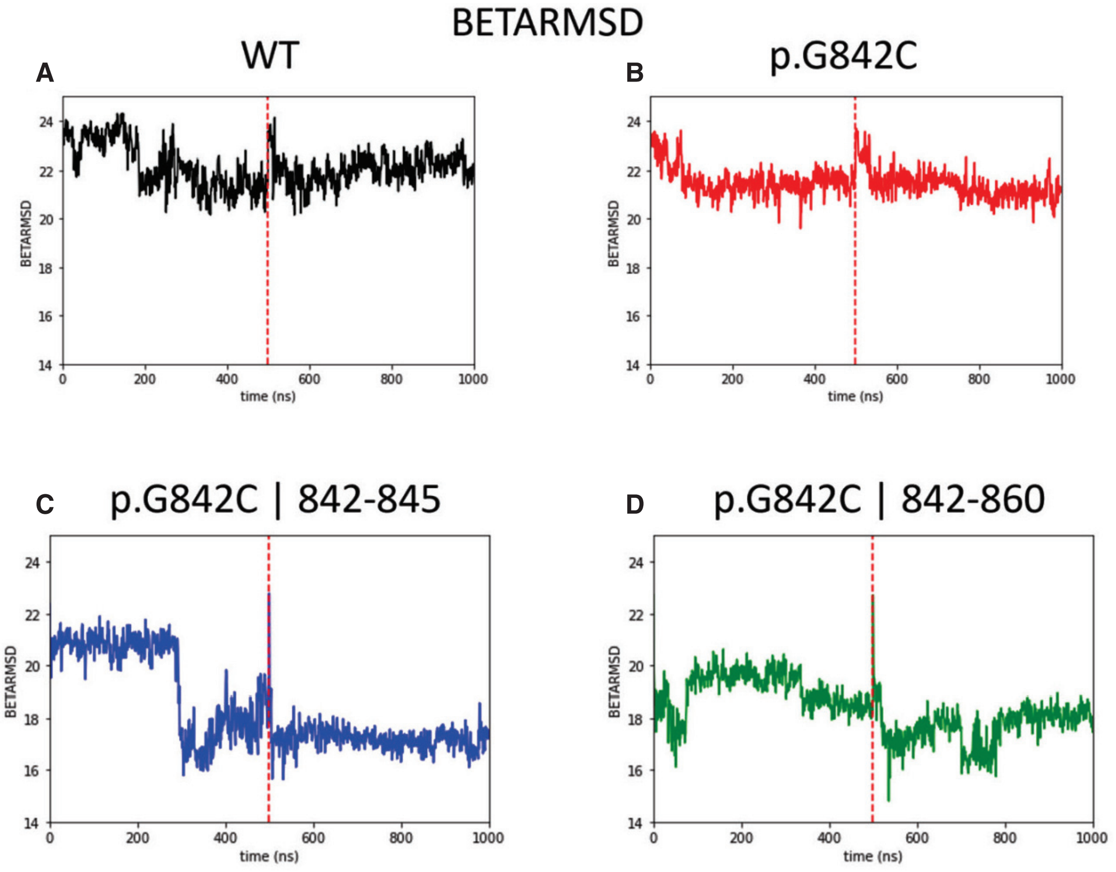

Figure EV1. BETARMSD plots of WT PlxnB2 and G842C-mutated isoforms.

A–D BETARMSD plots of the concatenated replicas of PlxnB2 WT, p.G842C, p.G842C 842–845, and p.G842C 842–860, respectively. The plots report the beta-sheet content evolution among the four studied systems, using the BETARMSD score described in the Materials and Methods section. In panel A, it is reported the time course of the selected parameter for the two concatenated MD simulations of the wild-type protein. It is evident that, starting from the initial state, a small rearrangement of the secondary structure is present even if the parameter remains quite stable along the simulation as identified by the average value obtained that is <BETARMSDWT> = 21.9 ± 0.5. The behavior of the same parameter for the point mutation of G842 in a cysteine residue, still maintaining the native disulfide bond, is reported in panel B, and it shows a slight decrease of beta-sheet content (<BETARMSDp.G842C> = 20.4 ± 0.4), compared with WT due to the loss of a single beta strand (βG, as it is shown in Fig 3A in the main text). The same point mutation coupled with alternative disulfide bonds leads to a more relevant loss of beta-sheet content in both cases (<BETARMSDp.G842C|842–845> = 17.6 ± 1.19, <BETARMSDp.G842C|842–860> = 18.4 ± 0.80) due to the disruption of βG, βC, and βD strands (see Fig 3D and E in the main text).

Figure EV2. RMSD plots of additional PlxnB2 ectodomain mutants found in human tumors.

A–D RMSD plots of two independent repetitions of 250 ns molecular dynamics of diverse PlxnB2 IPT3 domain mutants found in human tumors. (A) Mutant R820H; (B) mutant L828F; (C) mutant R843Q; (D) mutant Y852C.

E, F RMSD plots of two independent repetitions of 250 ns molecular dynamics of additional PlxnB2 mutants (outside IPT3 domain) found in CUP samples: (E) R531P and (F) P1058S.

G, H RMSD plots of two independent repetitions of 500 ns molecular dynamics of wild-type PlxnB2 and G842C mutant, extracted from Fig 3 and reported here for internal reference.

© 2023 The Authors

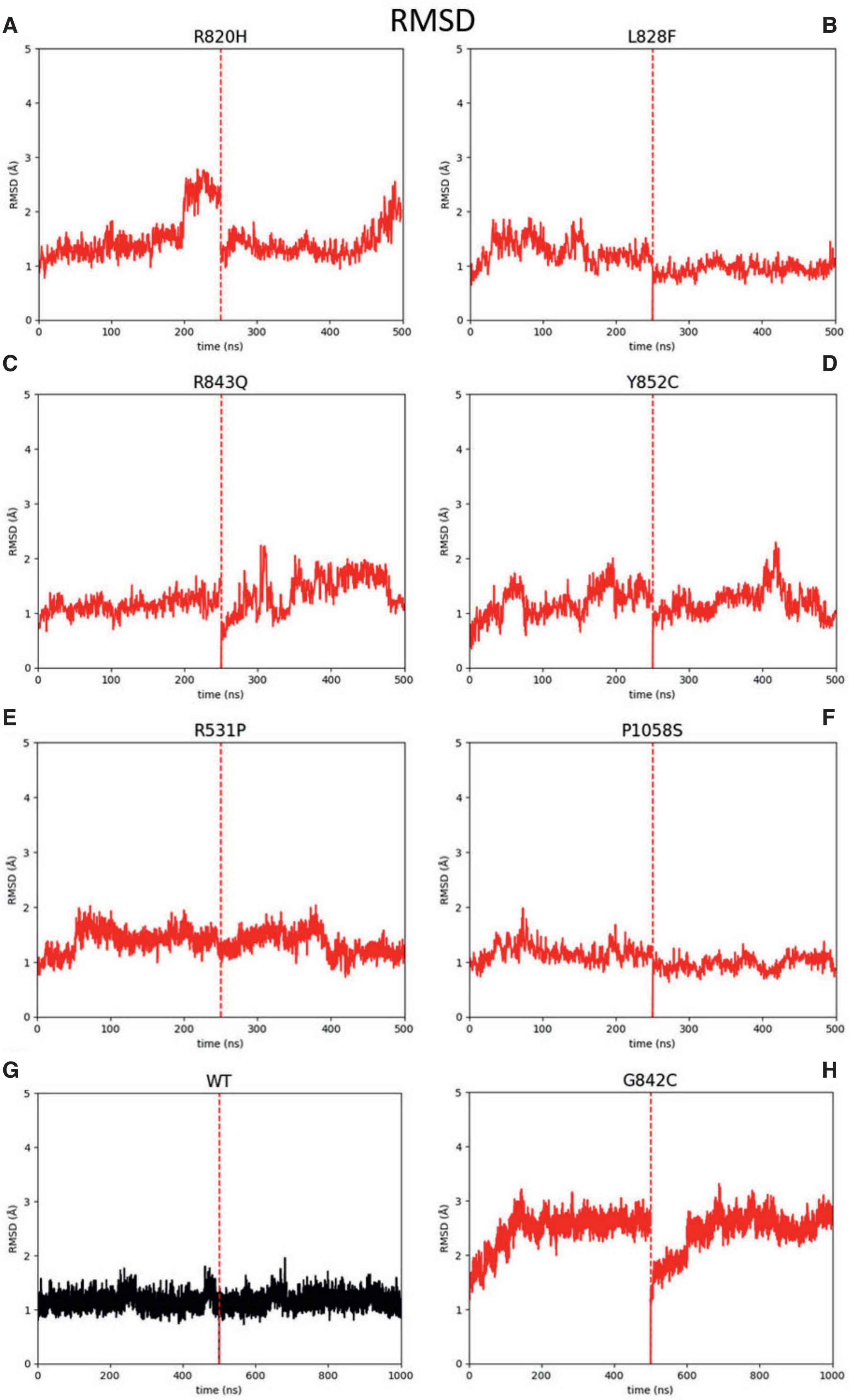

Figure EV2.

 © 2023 The Authors

Figure EV3. Validation of CUP models with modified expression of PlxnB2 WT or G842C mutant.

A Western blotting analysis of PlxnB2 expression in the indicated CUP-derived agnospheres, either subjected to gene knockdown by lentiviral-mediated transfer of targeted shRNAs (shPlxnB2) or transduced with a nontargeting control sequence (shScramble).

B Western blotting analysis of PlxnB2 expression in CUP-derived AS67, engineered by lentiviral-mediated transfer of an empty vector control (mock), nontargeting scrambled shRNAs, PlxnB2-targeted shRNAs, a wild-type PlxnB2 expression construct, or a G842C-mutated PlxnB2 expression construct PlxnB2-G842C, respectively.

C Western blotting analysis of PlxnB2 expression in AS901 and AS906 (as indicated), engineered to overexpress either wild-type PlxnB2 or PlxnB2-G842C, or transduced with an empty vector (mock).

D Western blotting analysis of PlxnB2 expression in AS43 subjected to gene knockdown by lentiviral-mediated transfer of an alternative independent shRNA sequence (shPlxnB2 #2, see Materials and Methods), or transduced with a corresponding empty vector (shCtrl).

E Time course analysis of cellular viability in shPlxnB2-tranduced and control agnospheres described in the previous panel, over 7 days of growth in culture. Values are mean $\pm$ SD of $n = 3$ independent experiments (with quadruplicate technical replicates). The statistical significance was assessed by 2-way ANOVA multiple comparisons for each time point, with Bonferroni correction. shCtrl vs. shPlxnB2#2(1): at day 3 $^{**}P = 0.0086$, day 4 $^{**}P = 0.0047$; $^{****}P < 0.0001$. shCtrl vs. shPlxnB2#2(2): $^{\$}P = 0.0161$; $P < 0.0001$.

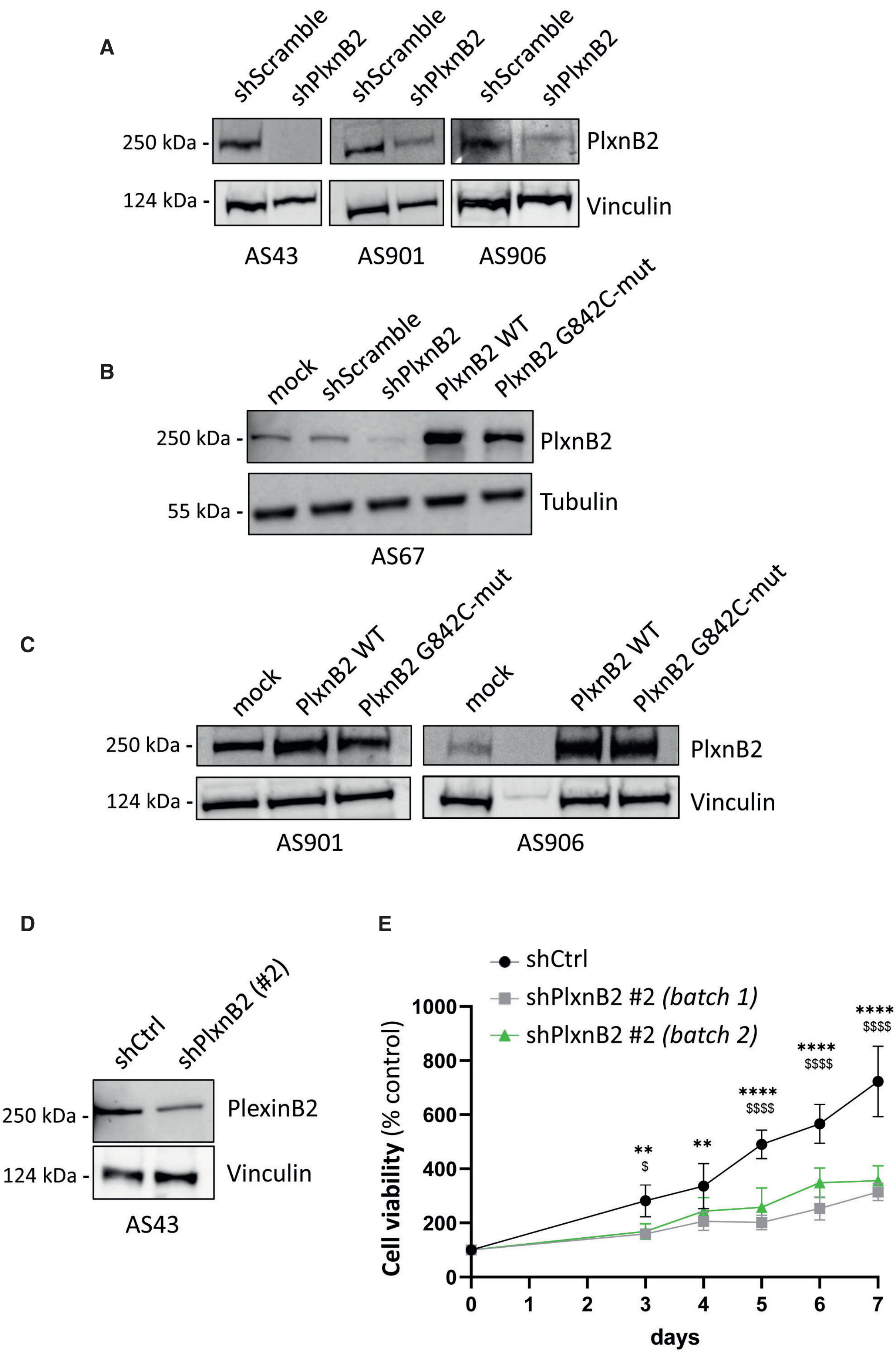

Figure EV3.

 © 2023 The Authors

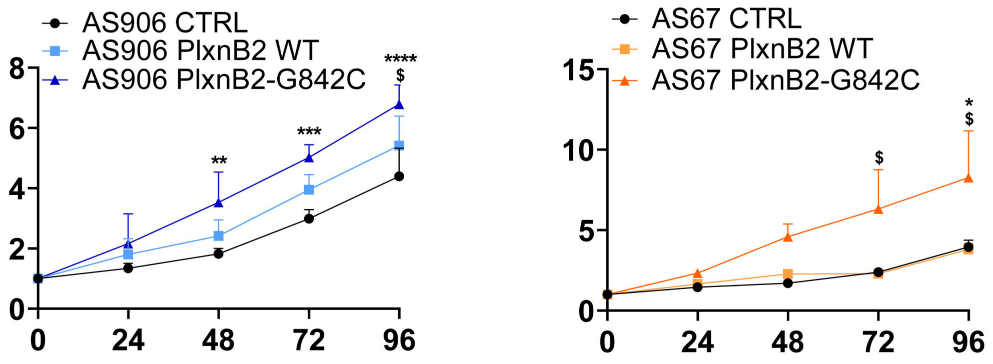

Figure EV4. Differential regulation of CUP cell growth by WT PlxnB2 or G842C mutant.

Time course cell viability analysis of AS906 (on the left) and AS67 (on the right) overexpressing either wild-type or PlxnB2-G842C, or mock controls transduced with an empty vector. Plotted values are the mean ± SD of $n = 3$ independent experiments (six technical replicates for each). The statistical significance was assessed by two-way ANOVA test. AS906 PlxnB2-G842C vs. controls (CTRL): at 48 h $^{**}P = 0.0042$, 72 h $^{***}P = 0.0007$, 96 h $^{****}P < 0.0001$. AS906 PlxnB2-G842C vs. PlxnB2-WT: at 96 h $^{\$}P = 0.0224$. AS67 PlxnB2-G842C vs. controls (CTRL): at 96 h $^{*}P = 0.0337$. AS67 PlxnB2-G842C vs. PlxnB2-WT: at 72 h $^{\$}P = 0.0494$, 96 h $^{\$}P = 0.0274$.

© 2023 The Authors

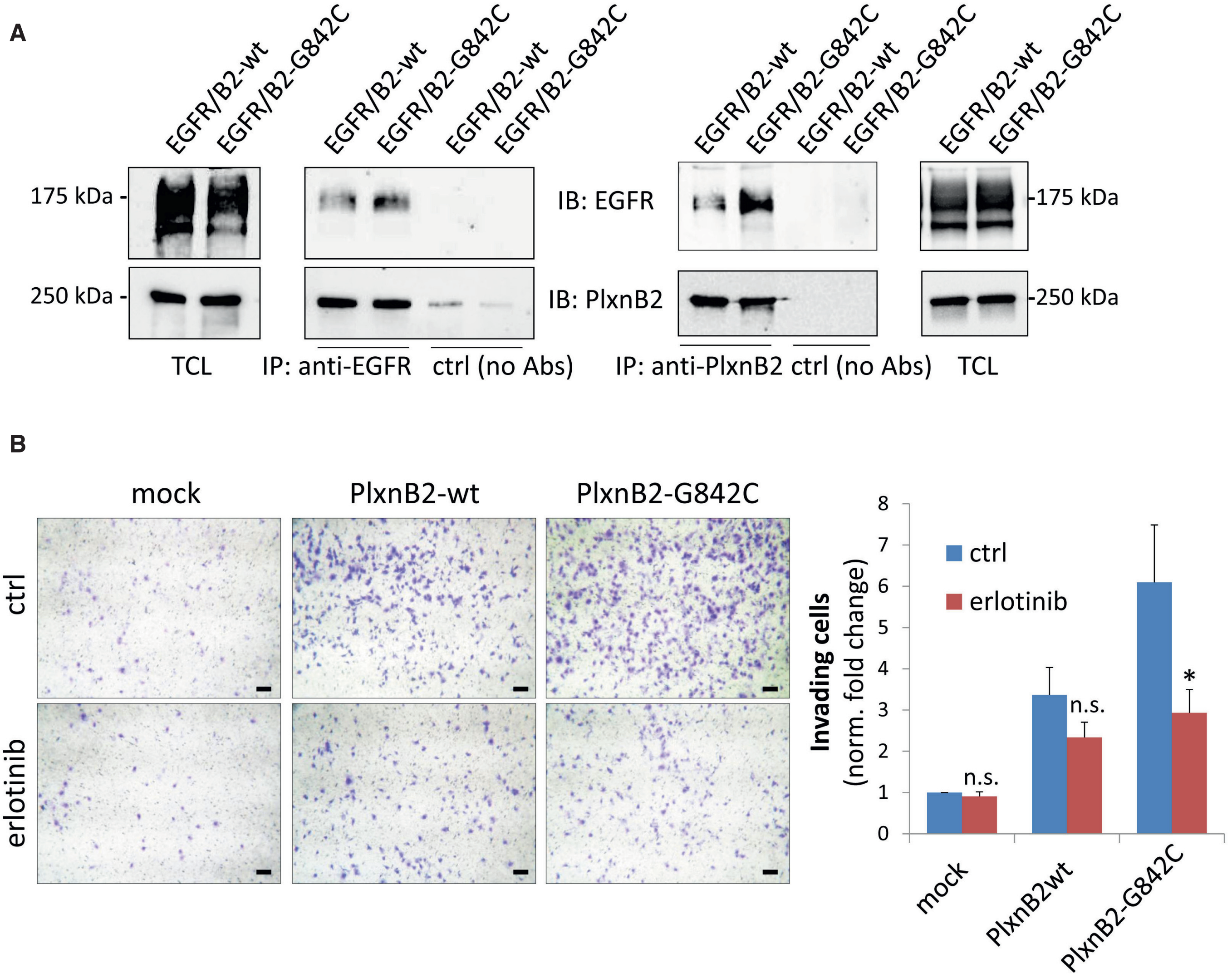

Figure EV5. PlxnB2-EGFR complex and EGFR-dependent regulation of CUP cell invasiveness.

A HEK293T cells were co-transfected with EGFR and either wild-type or PlxnB2-G842C. On the left, total cell lysates (TCL) were subjected (or not) to immunoprecipitation with anti-EGFR, and immunoblotted with the indicated antibodies. Data shown are representative of $n = 3$ experiments. On the right, EGFR-PlxnB2 co-immunoprecipitation was reciprocally assessed in HEK293T cells co-transfected as above, by subjecting total cell lysates to immunoprecipitation with anti-PlxnB2 antibodies. Data shown are representative of $n = 2$ experiments.

B Similar to the experiment shown in main Fig 9D, the invasiveness of AS906 cells either mock, or overexpressing WT or PlxnB2-G842C, was assessed in matrigel-coated Transwell inserts, in the presence or absence of the EGFR inhibitor erlotinib 1 μM. Invading cells were stained with crystal violet, photographed (see representative low magnification images on the left; scale bar: 100 μm), and quantified by ImageJ. Plotted values are the mean ± SD of $n = 3$ independent experiments. The statistical significance across replicates was verified by unpaired t-test multiple comparisons between erlotinib-treated and untreated conditions, per each group: $^*P = 0.021$.

 © 2023 The Authors

Brundu et al.

Mutation of the axon guidance gene PLXNB2 sustains proliferative autonomy and confers invasive properties to stem cells isolated from Cancers of Unknown Primary

APPENDIX

Table of Contents

- **Appendix Figure S1.** Histological images of CUP samples analyzed in this study *Page 2*
- **Appendix Figure S2.** Supplemental data on cell collapsing assays with PlnxB2 mutants *Page 3*
- **Appendix Figure S3.** Ligand binding assays comparing wild-type and G842C-PlxnB2 *Page 4*
- **Appendix Figure S4.** Supplemental images of cell migration/invasion assays *Page 5*

Appendix Figure S1

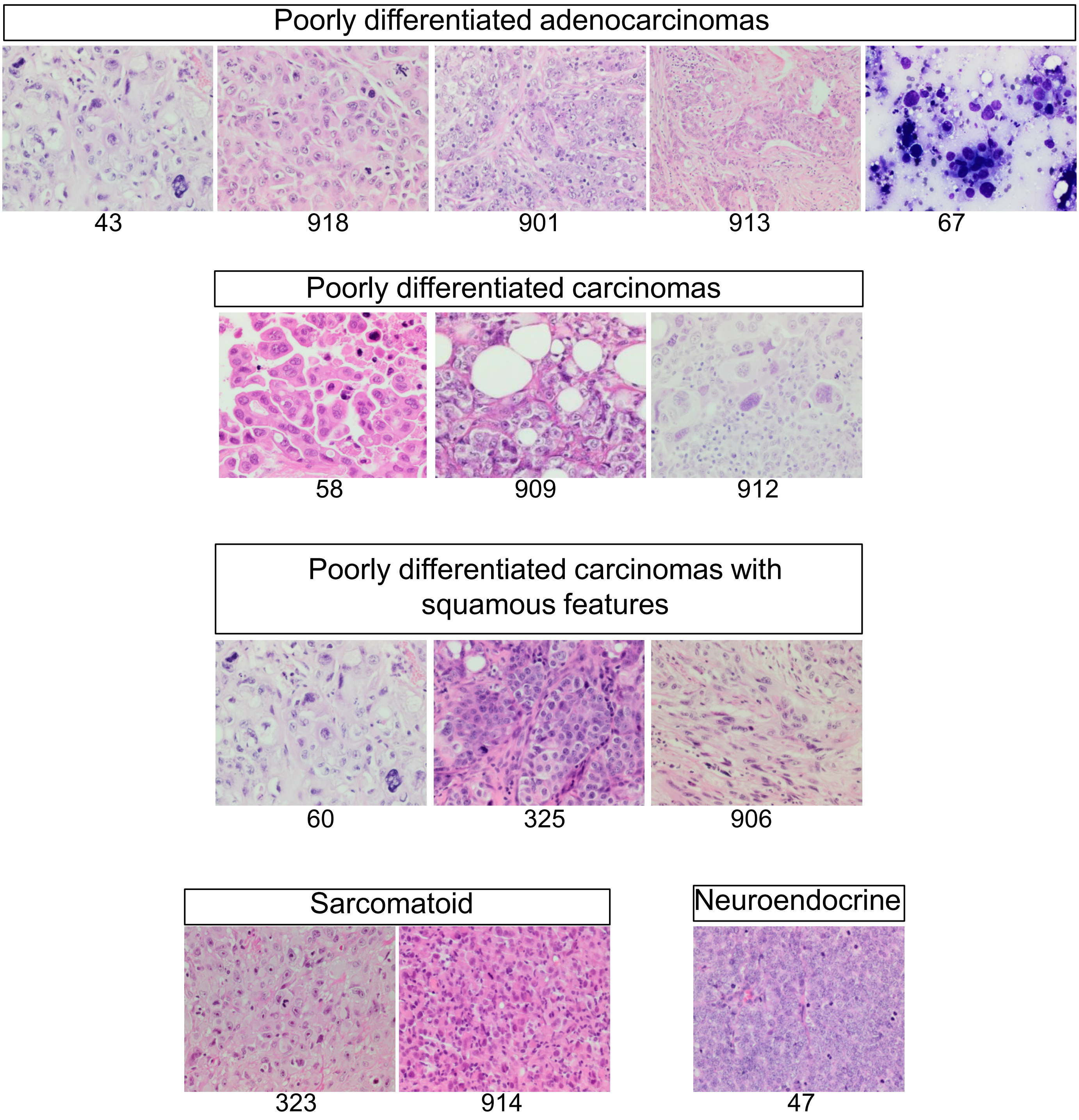

Appendix Fig. S1. Histological images of CUP samples analyzed in this study. Histological images (40x) of sectioned paraffin-embedded CUP samples (e.g. AGN43, AGN918, etc., as indicated in Table EV1), stained by Hematoxylin-Eosin staining, except for panel N (AGN67) which is a cytological specimen stained by Diff-Quick.

Appendix Fig. S2. Collapsing assays with PlnxB2 mutants. Immunofluorescence analysis of transfected COS7 cells expressing wild-type or mutant PlxnB2 variants. Cells expressing the WT receptor were also stimulated with 1 μg/ml Sema4C, in order to provide an internal positive control of the typical collapsing response. The area of around 50-100 representative cells per each condition (from multiple independent microscopic fields and experiments) was measured by ImageJ software, and the individual values were included in a violin plot (shown on the right), where the red line indicates the median value and yellow lines mark the first and third quartiles of the population. The statistical analysis was done by one-way ANOVA, comparing each of the groups with that of unstimulated WT cells: ****p<0.0001; *p<0.05.

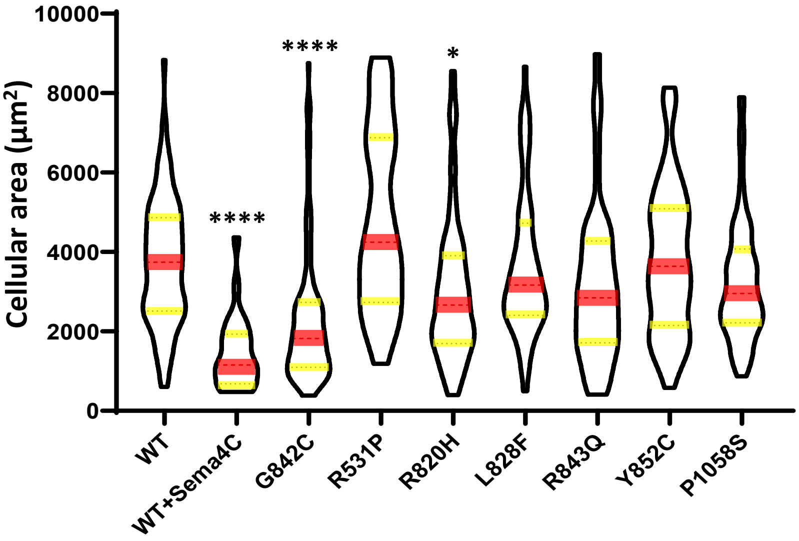

Below is shown a wide series of representative fields for the diverse conditions, presented as pairs of one image displaying anti-PlxnB2 staining in red, and one corresponding merge of fluorescent channels (including green staining of F-actin by phalloidin, and nuclear DAPI staining in blue). Scale bar: 50μm.

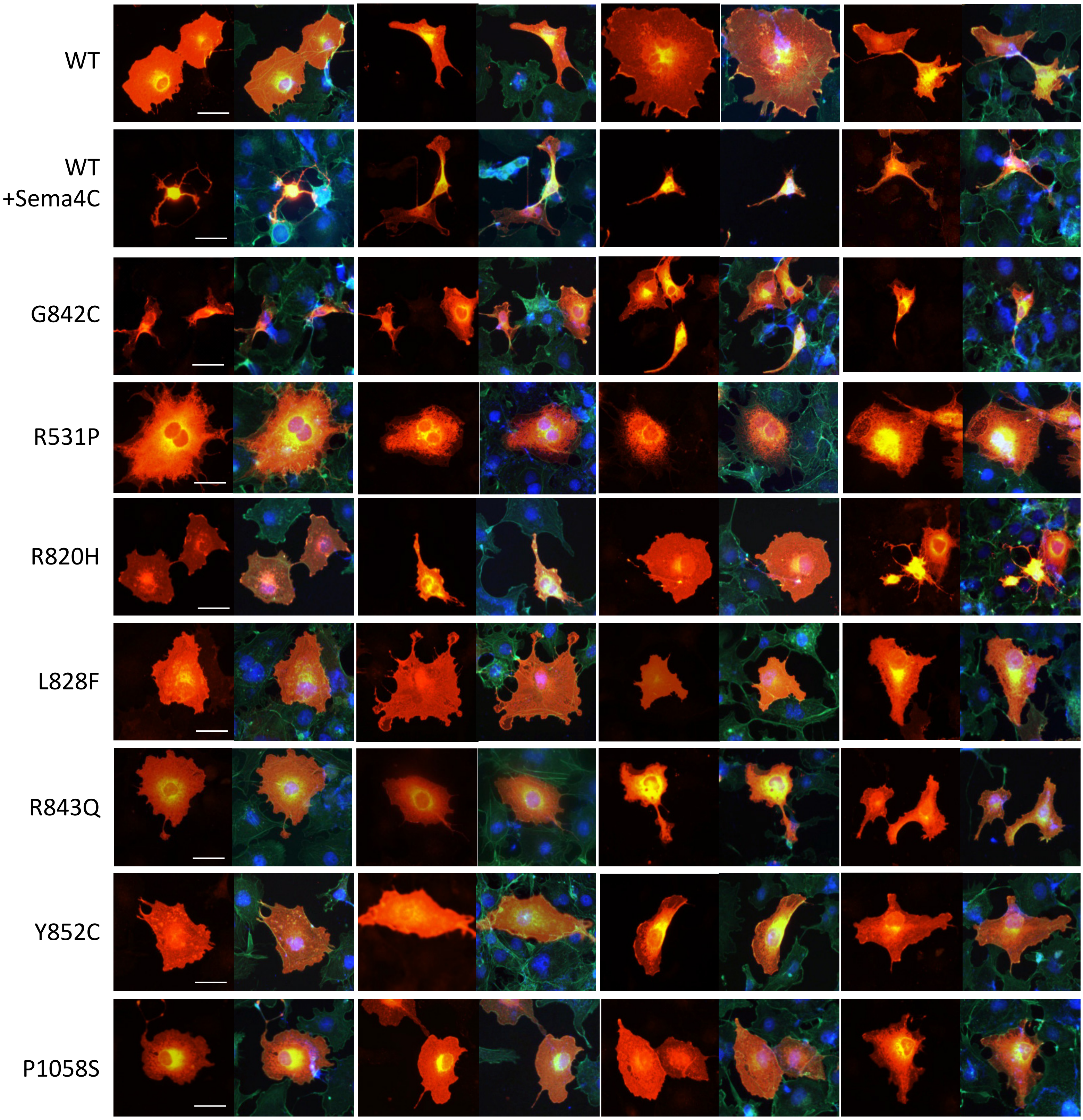

Appendix Figure S3

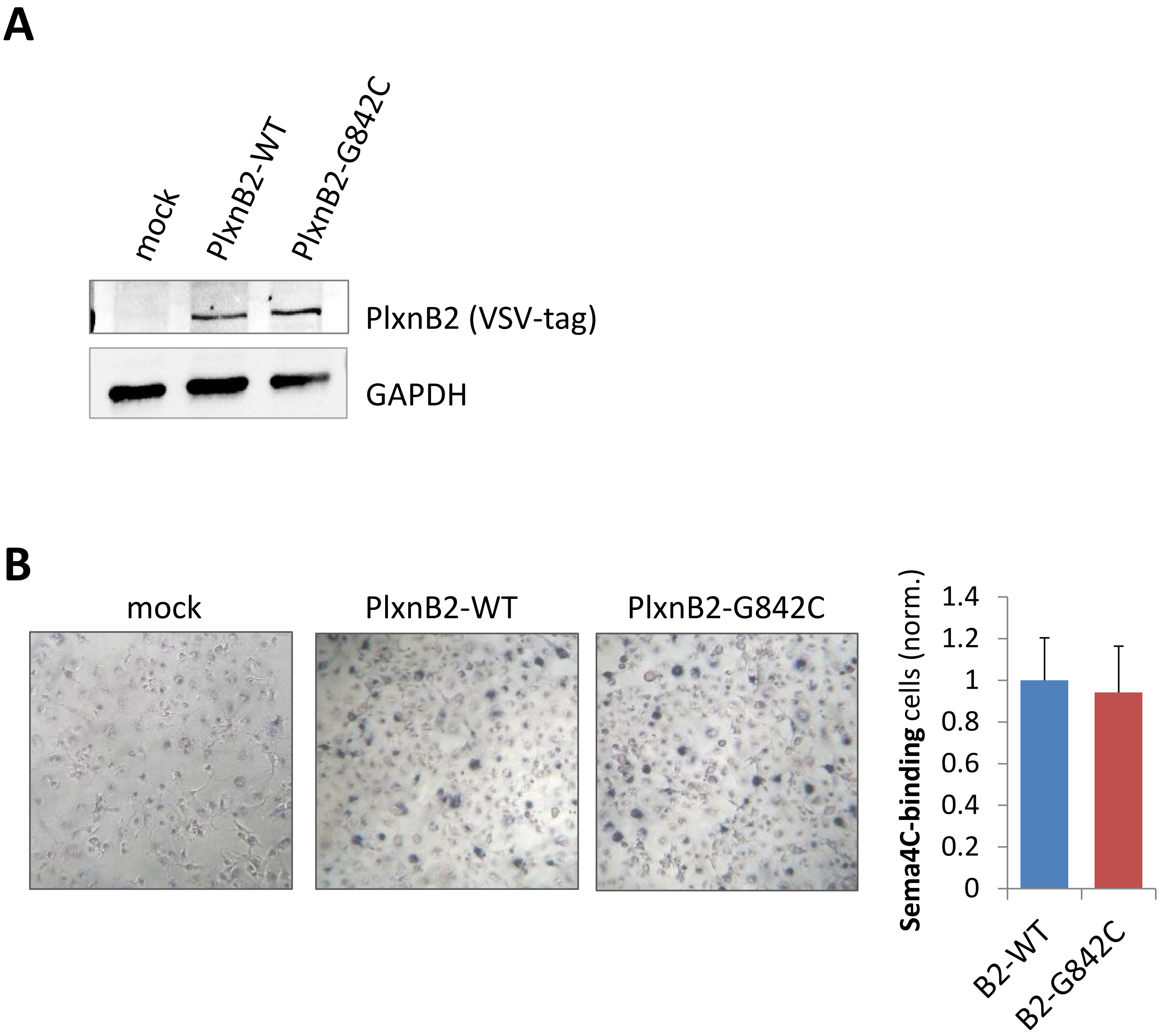

Appendix Fig. S3. Ligand binding assays comparing wild-type and G842C-PlxnB2

(A) The comparable expression of VSV-tagged wild-type (WT) and mutated PlxnB2 in transfected COS7 cells was verified by western blotting.

(B) Representative images of COS7 cells transfected with WT or G842-mutated PlxnB2 (analyzed in the previous panel) and probed with alkaline phosphatase-conjugated Sema4C, its cognate ligand, revealing comparable receptor binding. The graph at the bottom shows normalized mean values ± SD of the AP-labeled cell quantified in three experiments (after subtracting mock background signal).

Appendix Figure S4

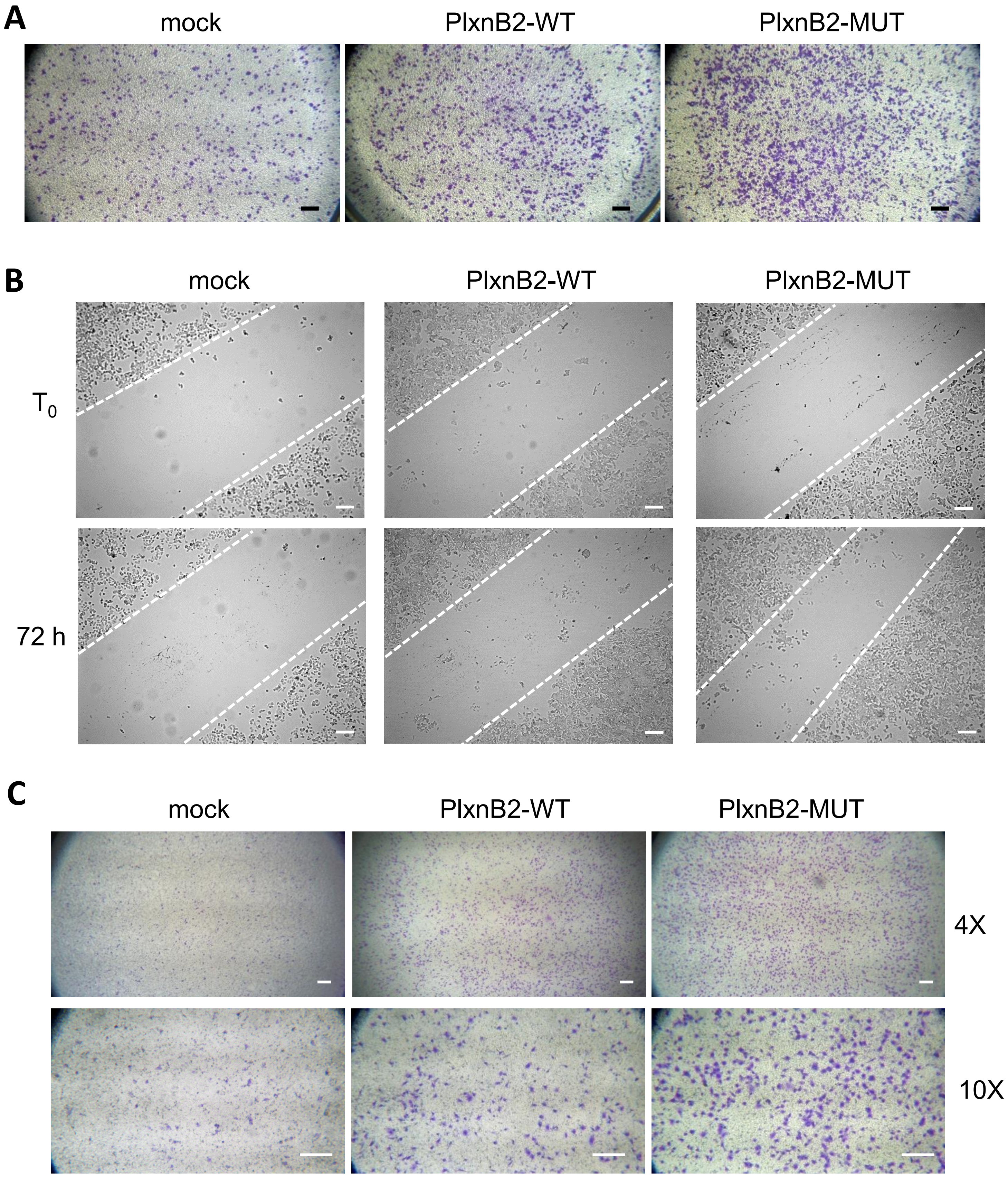

Appendix Fig. S4. Supplemental images of cell migration and cell invasion assays.
(A) Representative microscopic images of MCF-7 cells, either mock transfected, or overexpressing WT or G842C-mutated PlxnB2, migrated through Transwell inserts, then fixed and stained with crystal violet. Scale bar: 200 μm. Data quantification and statistical analysis across multiple replicates is shown in main Fig. 7B. (B) Representative phase contrast microscopic images of monolayers of the same MCF-7 cells as above (either mock, or overexpressing WT or G842C-mutated PlxnB2) immediately after scratching at T0, and then after 72 hours wound closure. Scale bar: 200 μm. Data quantification and statistical analysis across multiple replicates is shown in main Fig. 7C. (C) Representative microscopic images (at 4x or 10x magnification) of AS906 cells (either mock, or overexpressing WT or G842C-mutated PlxnB2), which had invaded across Matrigel-coated Transwell inserts, and were then fixed and stained with crystal violet. Scale bars: 200 μm. Data quantification and statistical analysis across multiple replicates is shown in main Fig. 7E.
