# Supplementary material for: Mutated axon guidance gene PLXNB2 sustains growth and invasiveness of stem cells isolated from cancers of unknown primary
Source: EMBO Mol Med. 2023 Feb 1;15(3):e16104. doi: 10.15252/emmm.202216104 (PMC9994481; doi:10.15252/emmm.202216104)

Fig.8A

AS43

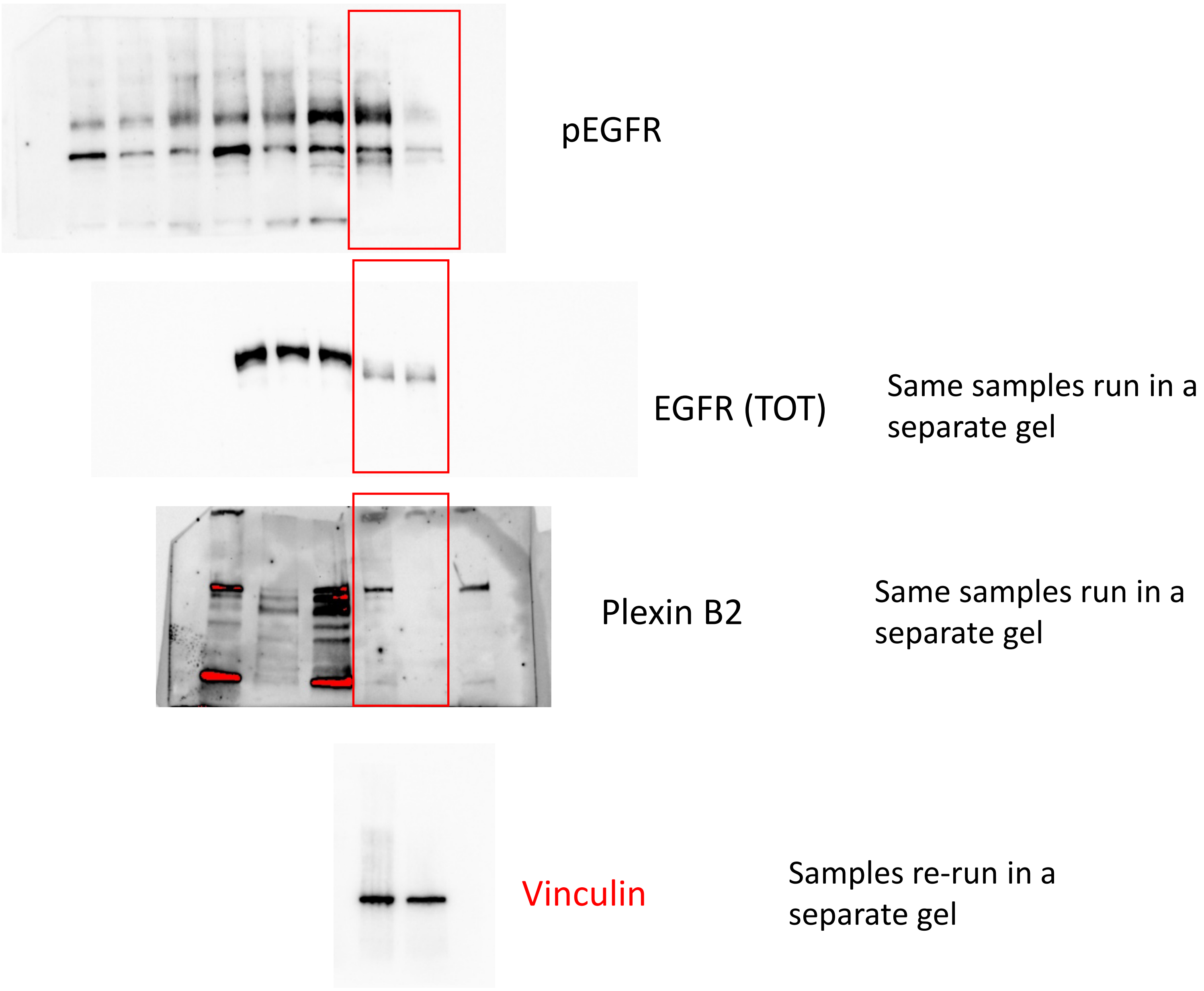
pEGFR
EGFR (TOT)
Same samples run in a separate gel
Plexin B2
Same samples run in a separate gel
Vinculin
Samples re-run in a separate gel

Fig.8B

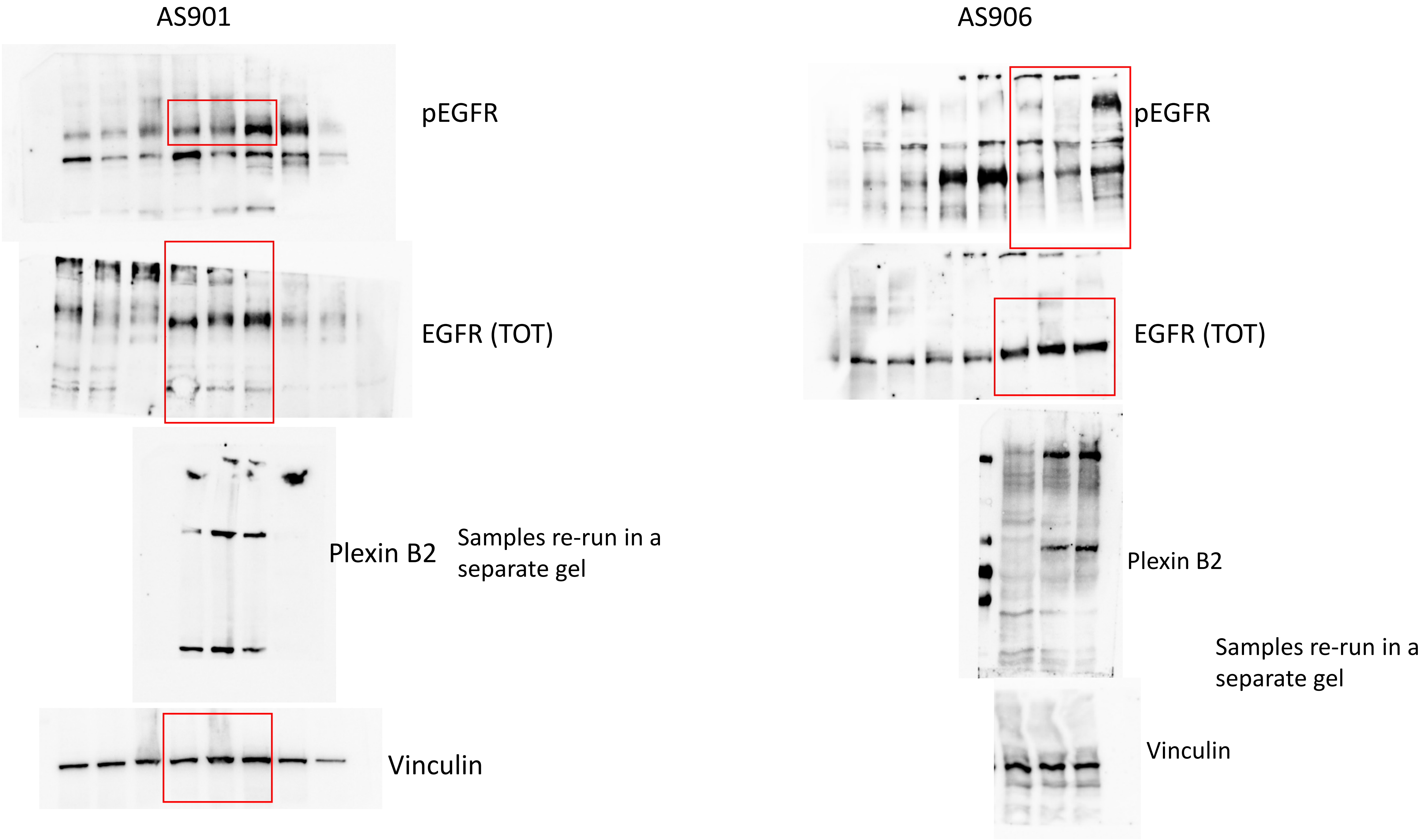
AS901
AS906
pEGFR
EGFR (TOT)
Plexin B2
Samples re-run in a separate gel
Vinculin
pEGFR
EGFR (TOT)
Plexin B2
Samples re-run in a separate gel
Vinculin

Fig.8C

AS43

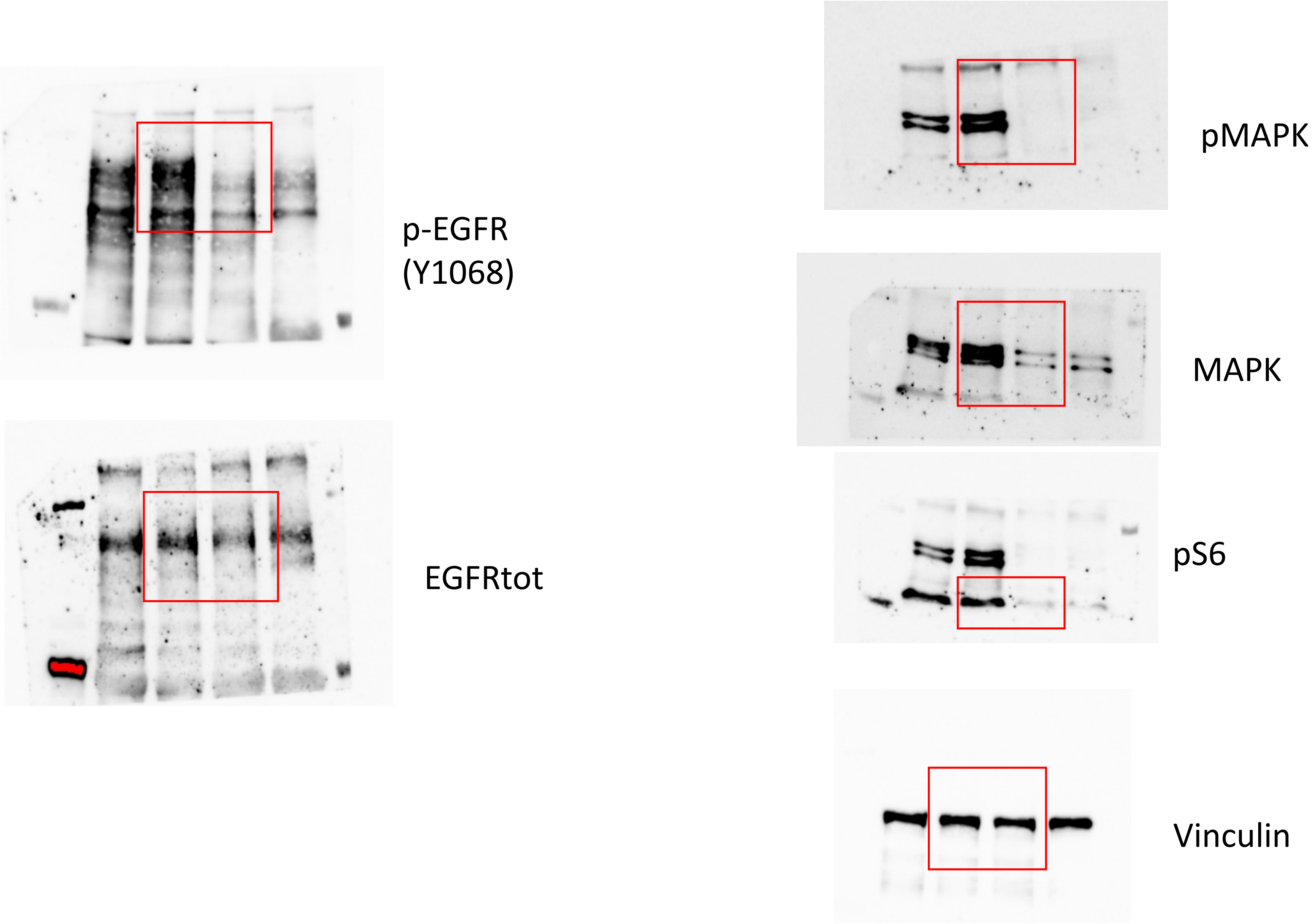

Fig.8C

AS901

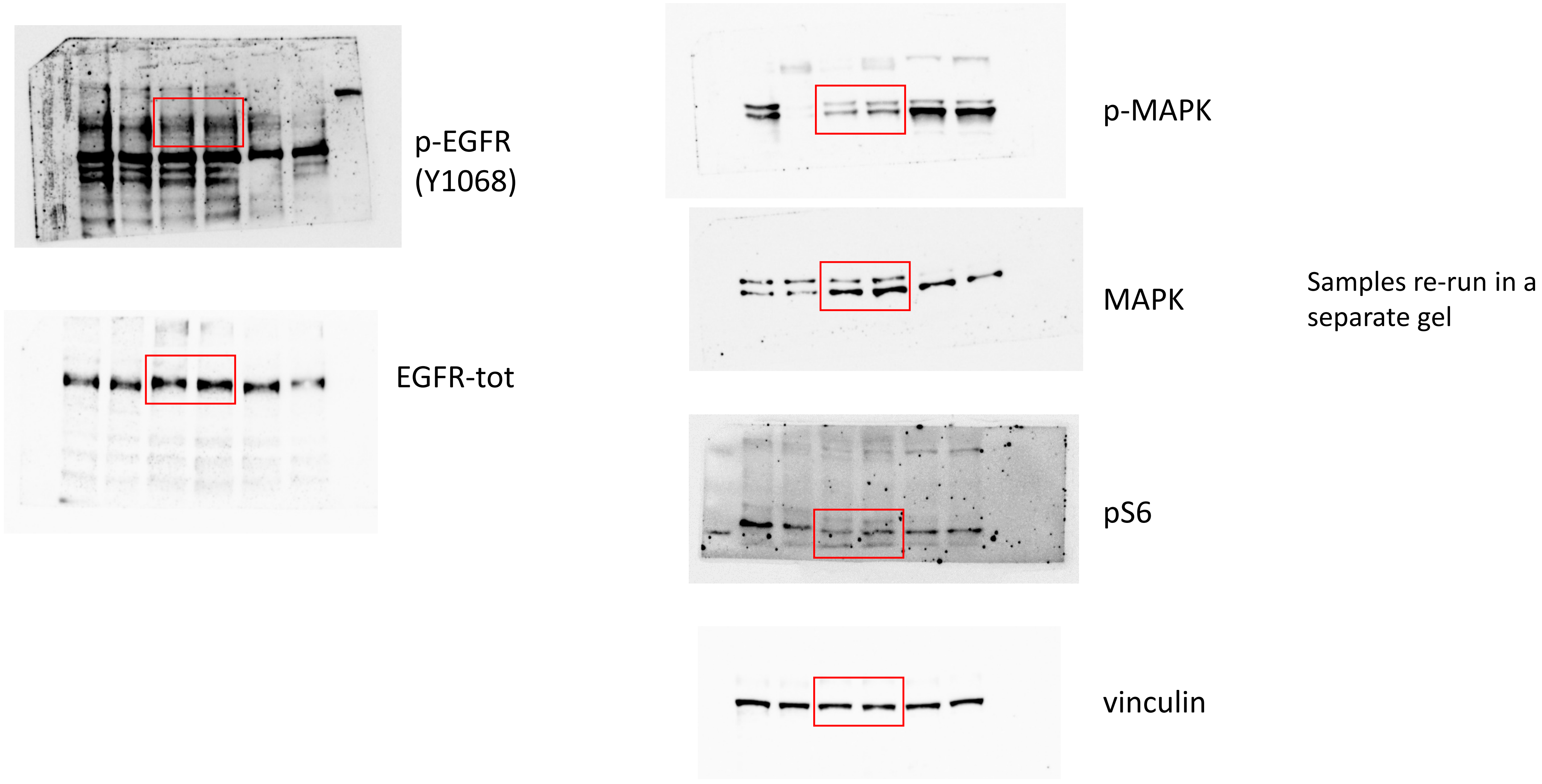
p-EGFR
(Y1068)
EGFR-tot
p-MAPK
MAPK
Samples re-run in a separate gel
pS6
vinculin

Fig.8C

AS906

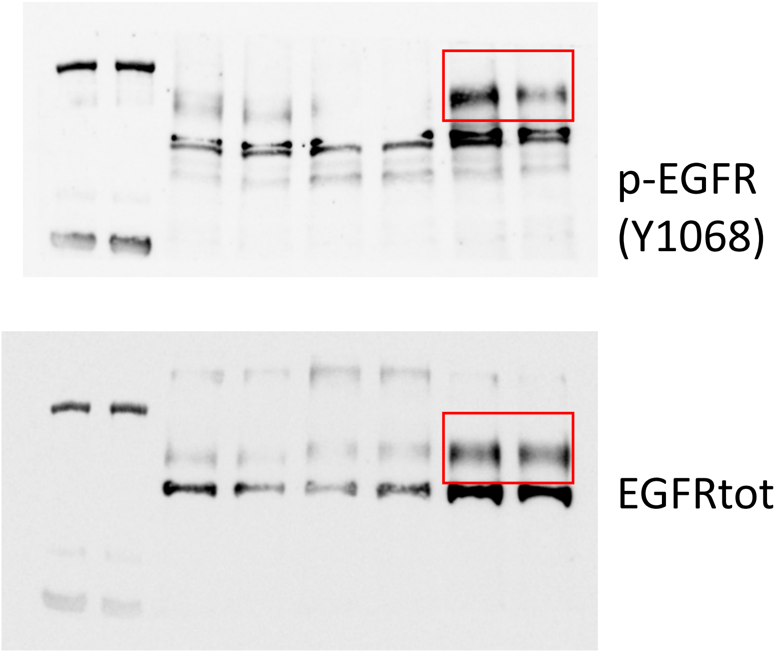

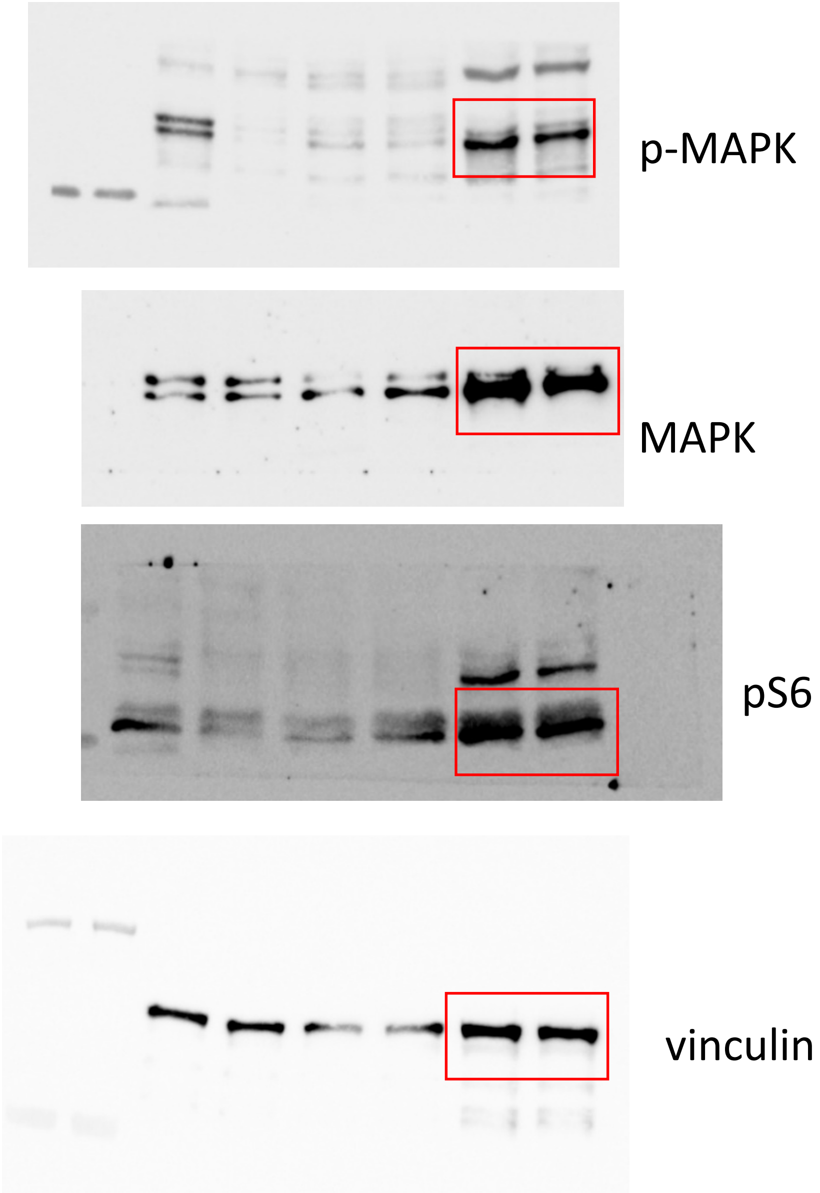

Supplement: Supplementary file 10 — Source Data for Figure 8 [file EMMM-15-e16104-s012.zip › Fig._8_raw_data_WB.pdf]
