# Supplementary material for: Mutated axon guidance gene PLXNB2 sustains growth and invasiveness of stem cells isolated from cancers of unknown primary
Source: EMBO Mol Med. 2023 Feb 1;15(3):e16104. doi: 10.15252/emmm.202216104 (PMC9994481; doi:10.15252/emmm.202216104)
Supplement: Supplementary file 12 [file EMMM-15-e16104-s011.pdf]

EMBO Molecular Medicine

Mutated axon guidance gene PLXNB2 sustains growth and invasiveness of stem cells isolated from Cancers of Unknown Primary

Serena Brundu, Virginia Napolitano, Giulia Franzolin, Ettore Lo Cascio, Roberta Mastrantonio, Gabriele Sardo, Eliano Cascardi, Federica Verginelli, Sergio Sarnataro, Gennaro Gambardella, Alberto Pisacane, Alessandro Arcovito, Carla Boccaccio, Paolo Comoglio, Enrico Giraudo, and Luca Tamagnone
DOI: 10.15252/emmm.202216104

Corresponding authors: Luca Tamagnone (luca.tamagnone@unicatt.it) , Enrico Giraudo (enrico.giraudo@ircc.it)

Review Timeline:

Submission Date:	31st Mar 22
Editorial Decision:	25th Apr 22
Revision Received:	28th Nov 22
Editorial Decision:	14th Dec 22
Revision Received:	28th Dec 22
Accepted:	11th Jan 23

Editor: Lise Roth

Transaction Report:

(Note: With the exception of the correction of typographical or spelling errors that could be a source of ambiguity, letters and reports are not edited. Depending on transfer agreements, referee reports obtained elsewhere may or may not be included in this compilation. Referee reports are anonymous unless the Referee chooses to sign their reports.)

25th Apr 2022

Dear Prof. Tamagnone,

Thank you for the submission of your manuscript to EMBO Molecular Medicine. We have now received feedback from the three reviewers who agreed to evaluate your manuscript. As you will see from the reports below, while referees #1 and #3 are overall supportive of publication pending minor revisions, referee #2 raises concerns on your work, both of technical and conceptual nature. In particular, this referee questions the relevance of the identified mutation for CUP in general, since it is a mixed tumor entity.
Upon cross-commenting, both referees #1 and #3 agreed that despite the uncertainty related to the difficulty in defining CUP cancers as a nosological entity, your findings could open the path to new therapeutic interventions.
They both requested that you confirm your observations in some other CUP models.

If you feel you can satisfactorily address this point and those listed by the referees, you may wish to submit a revised version of your manuscript. Please attach a covering letter giving details of the way in which you have handled each of the points raised by the referees. A revised manuscript will once again be subject to review and we cannot guarantee at this stage that the eventual outcome will be favorable.
EMBO Molecular Medicine encourages a single round of revision only and therefore, acceptance or rejection of the manuscript will depend on the completeness of your responses included in the next, final version of the manuscript. For this reason, and to save you from any frustrations in the end, I would strongly advise against returning an incomplete revision.

Revised manuscripts should be submitted within three months of a request for revision; they will otherwise be treated as new submissions, except under exceptional circumstances in which a short extension is obtained from the editor.

When submitting your revised manuscript, please carefully review the instructions that follow below. We perform an initial quality control of all revised manuscripts before re-review; failure to include requested items will delay the evaluation of your revision.

We require:

1) A .docx formatted version of the manuscript text (including legends for main figures, EV figures and tables). Please make sure that the changes are highlighted to be clearly visible.

2) Individual production quality figure files as .eps, .tif, .jpg (one file per figure). For guidance, download the 'Figure Guide PDF' (https://www.embopress.org/page/journal/17574684/authorguide#figureformat).

3) A .docx formatted letter INCLUDING the reviewers' reports and your detailed point-by-point responses to their comments. As part of the EMBO Press transparent editorial process, the point-by-point response is part of the Review Process File (RPF), which will be published alongside your paper.

4) A complete author checklist, which you can download from our author guidelines (https://www.embopress.org/page/journal/17574684/authorguide#submissionofrevisions). Please insert information in the checklist that is also reflected in the manuscript. The completed author checklist will also be part of the RPF.

5) Please note that all corresponding authors are required to supply an ORCID ID for their name upon submission of a revised manuscript.

6) It is mandatory to include a 'Data Availability' section after the Materials and Methods. Before submitting your revision, primary datasets produced in this study need to be deposited in an appropriate public database, and the accession numbers and database listed under 'Data Availability'. Please remember to provide a reviewer password if the datasets are not yet public (see https://www.embopress.org/page/journal/17574684/authorguide#dataavailability).
In case you have no data that requires deposition in a public database, please state so in this section. Note that the Data Availability Section is restricted to new primary data that are part of this study.

7) For data quantification: please specify the name of the statistical test used to generate error bars and P values, the number (n) of independent experiments (specify technical or biological replicates) underlying each data point and the test used to calculate p-values in each figure legend. The figure legends should contain a basic description of n, P and the test applied. Graphs must include a description of the bars and the error bars (s.d., s.e.m.).

8) We would also encourage you to include the source data for figure panels that show essential data. Numerical data should be provided as individual .xls or .csv files (including a tab describing the data). For blots or microscopy, uncropped images should be submitted (using a zip archive if multiple images need to be supplied for one panel). Additional information on source data and instruction on how to label the files are available at

.

9) Our journal encourages inclusion of *data citations in the reference list* to directly cite datasets that were re-used and obtained from public databases. Data citations in the article text are distinct from normal bibliographical citations and should directly link to the database records from which the data can be accessed. In the main text, data citations are formatted as follows: "Data ref: Smith et al, 2001" or "Data ref: NCBI Sequence Read Archive PRJNA342805, 2017". In the Reference list, data citations must be labeled with "[DATASET]". A data reference must provide the database name, accession number/identifiers and a resolvable link to the landing page from which the data can be accessed at the end of the reference. Further instructions are available at .

10) We replaced Supplementary Information with Expanded View (EV) Figures and Tables that are collapsible/expandable online. A maximum of 5 EV Figures can be typeset. EV Figures should be cited as 'Figure EV1, Figure EV2" etc... in the text and their respective legends should be included in the main text after the legends of regular figures.
- For the figures that you do NOT wish to display as Expanded View figures, they should be bundled together with their legends in a single PDF file called *Appendix*, which should start with a short Table of Content. Appendix figures should be referred to in the main text as: "Appendix Figure S1, Appendix Figure S2" etc.
- Additional Tables/Datasets should be labeled and referred to as Table EV1, Dataset EV1, etc. Legends have to be provided in a separate tab in case of .xls files. Alternatively, the legend can be supplied as a separate text file (README) and zipped together with the Table/Dataset file.
See detailed instructions here:
.

11) The paper explained: EMBO Molecular Medicine articles are accompanied by a summary of the articles to emphasize the major findings in the paper and their medical implications for the non-specialist reader. Please provide a draft summary of your article highlighting
- the medical issue you are addressing,
- the results obtained and
- their clinical impact.
This may be edited to ensure that readers understand the significance and context of the research. Please refer to any of our published articles for an example.

12) For more information: There is space at the end of each article to list relevant web links for further consultation by our readers. Could you identify some relevant ones and provide such information as well? Some examples are patient associations, relevant databases, OMIM/proteins/genes links, author's websites, etc...

13) Author contributions: the contribution of every author must be detailed in a separate section (before the acknowledgments).

14) Conflict of interest: We updated our journal's competing interests policy in January 2022 and request authors to consider both actual and perceived competing interests. Please review the policy https://www.embopress.org/competing-interests and update your competing interests if necessary.

15) Every published paper now includes a 'Synopsis' to further enhance discoverability. Synopses are displayed on the journal webpage and are freely accessible to all readers. They include a short stand first (maximum of 300 characters, including space) as well as 2-5 one-sentences bullet points that summarizes the paper. Please write the bullet points to summarize the key NEW findings. They should be designed to be complementary to the abstract - i.e. not repeat the same text. We encourage inclusion of key acronyms and quantitative information (maximum of 30 words / bullet point). Please use the passive voice. Please attach these in a separate file or send them by email, we will incorporate them accordingly.
Please also suggest a striking image or visual abstract to illustrate your article as a PNG file 550 px wide x 300-600 px high.

16) As part of the EMBO Publications transparent editorial process initiative (see our Editorial at http://embomolmed.embopress.org/content/2/9/329), EMBO Molecular Medicine will publish online a Review Process File (RPF) to accompany accepted manuscripts.
In the event of acceptance, this file will be published in conjunction with your paper and will include the anonymous referee reports, your point-by-point response and all pertinent correspondence relating to the manuscript. Let us know whether you agree with the publication of the RPF and as here, if you want to remove or not any figures from it prior to publication.
Please note that the Authors checklist will be published at the end of the RPF.

EMBO Molecular Medicine has a "scooping protection" policy, whereby similar findings that are published by others during review or revision are not a criterion for rejection. Should you decide to submit a revised version, I do ask that you get in touch after three months if you have not completed it, to update us on the status.

I look forward to receiving your revised manuscript.

Yours sincerely,

Lise Roth

Lise Roth, PhD
Editor
EMBO Molecular Medicine

***** Reviewer's comments *****

Referee #1 (Comments on Novelty/Model System for Author):

This paper describes a novel activating mutation of the axon guidance gene PLXNB2, and presents experimental data showing that this mutation sustains proliferative autonomy and confers invasive properties to stem cells isolated from Cancers of Unknown Primary.

The materials and methods are appropriate. The results are clearly explained and compelling.

The findings reported by the authors are interesting and could have a significant therapeutic impact, as they suggest to target EGFR in PlexinB2-mutated CUPs.

The main limitation of this work resides in the low number of tumor analyzed, which does not allow to draw firm conclusions and imposes a confirmation of the results in a larger patient sample. With this caveat in mind, the clues from this thouroughly analysed CUP case series are worth of consideration for the readership of the journal.

A have only a minor remark. No analysis of the correlation between molecular phenotype (in terms of PlexinB2 mutation) and histological characteristics is presented, probably due to the small sample size. A descriptive correlation summary could be added, as even an eventual lack of correlation would be interesting with regard to the hypothesis of the existance of a common molecular background in different CUP histological types.

Referee #2 (Comments on Novelty/Model System for Author):

The Cancers of Unknown Primary are a mixed bag of different tumors, no strong conclusions about the role of an individual mutation can be drawn from such a mixed tumor entity.

Referee #2 (Remarks for Author):

In this manuscript, Brundu and colleagues examine the role of mutated Plexin-B2 activity in Cancer of Unknown Primary (CUPs). By using whole-exome genomic profiling of CUP samples, they find a consistent enrichment of changes in genes belonging to the axon guidance KEGG pathway, and focused on a G842C mutation of Plexin-B2, which was characterized by the authors as a gain of function (GOF) mutation. This mutation impacts the self-renewal and proliferation of CUP cells in culture, as well as their tumorigenic capacity in mice. Mechanistically, the authors found that G842C-Plexin-B2 expression was associated with increased EGFR phosphorylation in CUP, which elicited CUP invasiveness, which was blocked by EGFR inhibitor treatment.

The findings regarding G842C gain-of-function mutation are interesting, and there are nice molecular dynamics and bioinformatics analyses performed in this manuscript.

Unfortunately, there appears to be a logical problem with the choice of investigating this particular Plexin-B2 mutation:
A gain of function mutation is typically identified by being recurrent in multiple independent patients. This is here not the case (found only in one patient, AGN43). Thus, there is no scientific support from the sequencing study for the GOF character of this mutation.
It is very possible that Plexin-B2 G842C is a passenger mutation with no significance.

The authors could use the cBio portal to assess the Plexin-B2 mutation spectrum in other well-defined cancers to identify potential GOF mutants.
Furthermore, CUP cancers are not a defined entity, but a mixed bag of different tumors, so no general conclusions can be drawn

on Plexin-B2 functions on CUP tumors, just on individual tumors.

The functional data about the Plexin-B2 G842C mutant are potentially novel and interesting. The mechanism by which this mutation controls cell morphology and invasiveness behavior of CUP remains however not fully clarified. The EGFR phosphorylation explains indirectly the CUP self-renewal and proliferation (although more direct biological experiments should be performed to show the causal connection between G842C mutant, p-EGFR, and self-renewal and proliferation). The only experiment touching this question was the treatment with erlotinib, but rather accessing invasiveness.

The paper provides little insight into how G842C mutation mechanistically functions, outside its link to p-EGFR. This aspect is seen on phenotypes such as changes in morphology and invasiveness, which are not well explained. More mechanistic studies are necessary to derive conclusions about the importance of G842C gain-of-function mutation for the morphological changes and migration.

What type of experiments could help confirm the GOF mutation? More sophisticated studies are needed to convincingly investigate GOF effects in cancer cells. Possible strategies: KO of Plexin-B2 in cancer cells and rescue with different mutant forms; CRISPR mediated knock-in of the point mutation; shRNAs that specifically target the mutant mRNA (assuming that mutation is heterozygous).

It is an interesting thought that the mutation may interfere with the auto-inhibitory head-to-tail formation of plexins that has been described by Kong et al. 2016 (PMID: 27397516; Fig. 3 therein), which should be considered. The relative conservation of the IPT3 domain inside the plexin structure should also be considered, see Junqueira Alves et al. 2019 (PMID: 30760850; Fig. 2 therein)

On a technical level, the data of cell-based assays and western blots is not convincing. In general, images are of low quality. It cannot be excluded that the effects are due to different expression levels of the Plexin-B2 forms in the cells, or other noise in the system, and not due to genuine GOF effects.

In the collapse assay, the single cells shown in Fig. 4C do not convince about the prevalent collapse phenotype in cells expressing G842C-Plexin-B2. The authors should show more cells and specify the criterion which they used to consider a collapsed cell, not only cells with one diameter lower than 40 µm were scored as collapsed (page 23), which seems quite arbitrary.

Figure 5, 6 and 7 lack representative images. In Fig. 8B panel is not possible to distinguish the cells. In Fig 8D panel the cells are very small and the images are of low quality. Fig. 9C lacks quantification.

Other comments:

1) The author should present the sequences for Plexin-B2 shRNAs used in the study. It is scientific best practice to use two independent shRNAs for knockdown, which should be performed here.
2) Western blots for Plexin-B2 should show both mature and precursor bands (see Artigiani et al., 2003), not just the cut out upper band of the precursor form, but also the mature form, which should be more important than the precursor in regard to biological function.
3) Western blots should have size indicators in kDa
4) Micrographs need scale bars
5) Figs.5 and 7 legends need to be revised. The P stars do not correspond to those observed in the figure.
6) The authors state that the stem cell frequency was strongly reduced in Plexin-B2-depleted AS43, but the Fig. 4B shows the opposite of that (shScramble 13% and shPlxnB2 25%).
7) The authors use t-test to compare two groups in experimental settings that require comparisons of three groups or more. Ex: Figs. 5A, 6A, 7A-B should use two-way ANOVA. Figs. S2 and 9B require one-way ANOVA or non-parametric test.
8) Fig. 4C requires statistical treatment.
9) Fig. 4A requires quantification.
10) The migration assays for MCF7 cancer cells are not properly described on page 13. I suggest move the Suppl. Fig.2 to Fig 8 and show representative images for that.
11) Fig. 8D is not mentioned in the main text.
12) The Co-IP images on Fig. 9C are not good for publication. The authors should provide better images, particularly for the total lysate.
13) The authors should perform statistical tests for the ctrl groups of Fig 9D as well.
14) It is suggested that Table 1 should go to the Sup. Materials.
15) Possible grammar mistake: "Landscaping" means shaping the landscape. The authors probably want to say "survey of the landscape"

Referee #3 (Remarks for Author):

In this paper the authors have demonstrated that a novel activating mutation of the axon guidance gene PLXNB2 plays an important role in the development of human Cancers of Unknown Primary (CUP). Moreover, it is interested that the mutated PlxnB2 elicited EGFR-dependent invasiveness of CUP, which was blocked by EGFR inhibitor treatment.
Overall, this is a very nice manuscript that shows new findings using human specimens. I have several questions that should be addressed, however.

1. Does the G842C-mutated PlxnB2 enhance the WT Plexin B2 signaling pathway or act on a different signaling pathway? If the former, do CUP cells with mutant PlexinB2 undergo the changes brought about by the PlexinB2-Sema4C signal? For example, collapsed phenotype similar to COS7, activation of RhoA / Rac1, enhancement of phosphorylation of MET in co-stimulation, etc.
2. Is the association of G842C-mutated PlxnB2 with EGFR found in HEK293T also confirmed in CUP cells?
3. In AS43, the mutant PlexinB2 promotes cell proliferation, but in MCF7, PlexinB2 overexpression (either wt or mutated) did not affect the growth rate. Is this difference the nature of the cells?
4. Is only cell invasion dependent on EGFR activity? Does it affect cell proliferation, stemness, or migration?
5. Fig 8B, authors mentioned that wound healing by cells overexpressing wild type PlxnB2 was slightly but not significantly increased, compared to controls, but it seems that there is a significant difference in the figure.
6. "Fig8C" in the text is "8D".

***** Reviewer's comments *****

Referee #1 *(Comments on Novelty/Model System for Author):*

This paper describes a novel activating mutation of the axon guidance gene PLXNB2, and presents experimental data showing that this mutation sustains proliferative autonomy and confers invasive properties to stem cells isolated from Cancers of Unknown Primary. The materials and methods are appropriate. The results are clearly explained and compelling. The findings reported by the authors are interesting and could have a significant therapeutic impact, as they suggest to target EGFR in PlexinB2-mutated CUPs. The main limitation of this work resides in the low number of tumor analyzed, which does not allow to draw firm conclusions and imposes a confirmation of the results in a larger patient sample. With this caveat in mind, the clues from this thoroughly analysed CUP case series are worth of consideration for the readership of the journal.

A have only a minor remark. No analysis of the correlation between molecular phenotype (in terms of PlexinB2 mutation) and histological characteristics is presented, probably due to the small sample size. A descriptive correlation summary could be added, as even an eventual lack of correlation would be interesting with regard to the hypothesis of the existence of a common molecular background in different CUP histological types.

Re: We are grateful to the Reviewer for his/her positive appreciation of our work. Collecting high quality samples from reliably selected CUP cases is a demanding endeavor. While our work provides the first example of genome-wide exome sequencing of CUPs, we concur on the fact that further studies will be required to consolidate and expand the significance of our findings.

In particular, *PLXNB2* mutations were found in three CUP cases (AGN43, AGN323, AGN325), which actually show distinct histopathological features. In the revised manuscript, we have integrated additional histopathological informations and mutational data for each CUP case in a summary Table EV1, now shown in the Expanded View section (as recommended by another reviewer). Moreover, we show representative histological images of these CUP samples (see Appendix Figure S1). As anticipated by the Reviewer, it was not possible to assess correlations between the molecular phenotype and the histological characteristics of this set of tumors. However, a comprehensive analysis of the available information on our panel of CUP cases indicates that mutations of Axon Guidance pathway genes are widely distributed (in over 70% of the cases), independent from the analyzed clinical parameters and histopathological features. This point is addressed in the revised manuscript in page 5 and 12.

Referee #2 *(Comments on Novelty/Model System for Author): The Cancers of Unknown Primary are a mixed bag of different tumors, no strong conclusions about the role of an individual mutation can be drawn from such a mixed tumor entity.*

Re: We respect the Reviewer's position. However, our work was inspired by the idea that common pathogenic mechanisms may be implicated in driving the typical presentation and clinical history of Cancers of Unknown Primary, as indicated by our previous experimental studies (Verginelli et al., Nat Comm. 2021, 12:2498) and discussed by us and others in reviews (Kolling et al., Front Oncol. 2020, 9:1546; Olivier et al. Cancer Treat Rev., 2021, 97:102204). We weren't necessarily in search of individual driver mutations, but rather sought for functionally linked genetic alterations. Our study provides a first comprehensive genomic analysis of CUP cases selected according to stringent criteria, and spotlights a significant enrichment in mutations of axon guidance pathway genes (compared to other tumor types), recurring in over 70% of the cases, independent from analyzed clinical parameters and histopathological features. G842C-PLXNB2 mutation, found in fourteen AGN43 metastatic lesions, as well as in the derived self-renewing agnospheres propagated ex-vivo, was further analyzed as prototypic example of the functional impact of a mutated axon guidance cue in CUP cells. Although genetic changes of other axon guidance genes have been implicated in human cancers, this is the first report describing the tumor-promoting potential of mutated *PLXNB2*.

(Remarks for Author): In this manuscript, Brundu and colleagues examine the role of mutated Plexin-B2 activity in Cancer of Unknown Primary (CUPs). By using whole-exome genomic profiling of CUP samples, they find a consistent enrichment of changes in genes belonging to the axon guidance KEGG pathway, and focused on a G842C mutation of Plexin-B2, which was characterized by the authors as a gain of function (GOF) mutation. This mutation impacts the self-renewal and proliferation of CUP cells in culture, as well as their tumorigenic capacity in mice. Mechanistically, the authors found that G842C-Plexin-B2 expression was associated with increased EGFR phosphorylation in CUP, which elicited CUP invasiveness, which was blocked by EGFR inhibitor treatment.
The findings regarding G842C gain-of-function mutation are interesting, and there are nice molecular dynamics and bioinformatics analyses performed in this manuscript. Unfortunately, there appears to be a logical problem with the choice of investigating this particular Plexin-B2 mutation: A gain of function mutation is typically identified by being recurrent in multiple independent patients. This is here not the case (found only in one patient, AGN43). Thus, there is no scientific support from the sequencing study for the GOF character of this mutation.
It is very possible that Plexin-B2 G842C is a passenger mutation with no significance.
The authors could use the cBio portal to assess the Plexin-B2 mutation spectrum in other well-defined cancers to identify potential GOF mutants.
Furthermore, CUP cancers are not a defined entity, but a mixed bag of different tumors, so no general conclusions can be drawn on Plexin-B2 functions on CUP tumors, just on individual tumors.

Re: As discussed above, we focused our attention on G842C-PLXNB2 mutation due to the availability of an ex-vivo model carrying the mutation, in which loss-of-function experiments could be performed; moreover, although genetic changes in members of the semaphorin/plexin family have been identified previously in other tumor types, their functional relevance had not been investigated. Our conclusion about G842C-PLXNB2 being a gain of function mutation is based on its capacity to trigger the typical plexin-induced cell collapsing effect, in a ligand-independent manner, in experimental models. Moreover, the knock-down of mutated, but not wild type, PlxnB2 in CUP cells resulted in reduced cell viability, clonogenic potential, and tumorigenesis in mice; and it was associated with reduced EGFR phosphorylation. By a complementary approach, the same

activities were upregulated into CUP cells carrying the wild type gene upon the transfection of G842C-PLXNB2. Notably, the expression of this PlxnB2 genetic variant was sufficient to increase cell viability and EGFR activation in multiple CUP models, independently from the mutational background.

These findings seem to speak against the hypothesis that *PLXNB2*-G842C is a passenger mutation with no significance. However, we agree with the Reviewer that analyzing a broader panel of CUP cases will allow validating and expanding the significance of our findings. Unfortunately, collecting high quality samples from reliably selected CUP cases is a demanding endeavor, which will progress beyond this study.

As far as we can understand from the current literature, recurrence in multiple independent patients is not necessarily a hallmark of GOF mutations. Indeed, previously unassigned mutations which impact on protein conformation, rather than targeting specific catalytic functions, have been recognized to be gain-of-function (Li et al, 2019; Gerasimavicius et al, 2022). We posit that G842C variation of the IPT3 domain of PlxnB2 results in a conformational change leading to receptor signaling activation. We and others have previously shown that non-mutated PlxnB1 and PlxnB2 receptors are functionally coupled with tyrosine kinases Met and ErbB2 in cancer cells, in ligand-dependent manner (e.g. Giordano et al, 2002; Swiercz et al, 2008; Song et al, 2012; Sun et al, 2012; Worzfeld et al, 2012; Damola et al. 2013; Le et al, 2015; Peacock et al, 2018; Li et al, 2018.); thus, our novel finding that a mutated PlxnB2 impacts on EGFR activity is consistent with this scenario.

As suggested by the Reviewer, we have searched public databases for other *PLXNB2* mutations in human cancers. While G842C is confirmed to be novel, a few other mutations in the same IPT3 domain of PlxnB2 have been reported in other tumor types. We thus analyzed their impact on receptor structure in silico (Figure EV2), and generated the corresponding expression constructs in order to experimentally test their activity. Our results indicate that no other mutation compares to G842C, found in AGN43, in functional terms (including R843Q change in the adjacent residue, or the nearby Y852C that also implicates the insertion of an unpaired cysteine residue) (Fig. 4A-B). This seems to suggest that G842C mutation is specifically endowed with functional prerogatives which cannot be achieved by other changes in the amino acid sequence in this protein domain.

The functional data about the Plexin-B2 G842C mutant are potentially novel and interesting. The mechanism by which this mutation controls cell morphology and invasiveness behavior of CUP remains however not fully clarified. The EGFR phosphorylation explains indirectly the CUP self-renewal and proliferation (although more direct biological experiments should be performed to show the causal connection between G842C mutant, p-EGFR, and self-renewal and proliferation). The only experiment touching this question was the treatment with erlotinib, but rather accessing invasiveness. The paper provides little insight into how G842C mutation mechanistically functions, outside its link to p-EGFR. This aspect is seen on phenotypes such as changes in morphology and invasiveness, which are not well explained. More mechanistic studies are necessary to derive conclusions about the importance of G842C gain-of-function mutation for the morphological changes and migration. What type of experiments could help confirm the GOF mutation? More sophisticated studies are needed to convincingly investigate GOF effects in cancer cells. Possible strategies: KO of Plexin-B2 in cancer cells and rescue with different mutant forms; CRISPR mediated knock-in of the point mutation; shRNAs that specifically target the mutant mRNA (assuming that mutation is heterozygous).

Re: We concur with the Reviewer that certain aspects of the mechanism of action of the PlxnB2 G842C mutant could not be fully elucidated in the previous version of the work. However, in the

revised manuscript we have added new experimental evidence that supports our conclusions. In particular, we found that the viability (and clonogenic capacity) of CUP cells carrying G842C-mutated but not wild-type PlxnB2 is dependent on EGFR signaling, as demonstrated by its blockade with the monoclonal antibody cetuximab (new Fig. 9A-9B-9C). Consistently, the treatment with EGFR inhibitors abated ERK and rpS6 phosphorylation in cells carrying G842C-PLXNB2, but not in cells bearing the wild-type receptor (new Fig. 8C). Moreover, the increase in cell invasiveness driven by the transduction of mutated PlxnB2 was abrogated by the treatment with both cetuximab and the selective kinase inhibitor erlotinib, indicating that EGFR was mechanistically responsible for it (Fig. 9D and expanded view supplemental Figure EV5-B). Of importance, it should be noted that the addition of G842C-PlxnB2 was sufficient to induce EGFR activation in two diverse CUP cell models (Fig 8B), independently from the mutational background.

We have discussed in response to the previous Reviewer's comment the evidence supporting our conclusion that G842C mutation mediates a gain-of-function. In fact, fully elucidating the mechanism by which this mutation leads to PlxnB2 activation is a tantalizing goal that appears beyond the scope of our study. We have illustrated, by means of structural analyses in silico, that this mutation is disrupting the conformation of the IPT3 domain of the receptor. As discussed above, in the revised manuscript we present evidence that other mutations found in human cancers in the IPT3 domain of PlxnB2 do not have the same functional impact. The role of IPT3 domain in plexin activation is not currently known; however, as rightfully suggested by the Reviewer (see next point), its evolutionary conservation suggests functional importance, consistent with our experimental evidence upon the impact of G842C mutation.

Notably, the change in cell morphology known as "cellular collapsing" is a classical readout of plexin signaling. Its mechanisms are partly understood, and likely to depend on the regulation of integrin-based focal adhesions. This is not a cancer cell-specific effect, but it has been widely applied to reveal plexin activation in experimental models. On the other hand, the role of EGFR signaling (activated by mutant PlxnB2) to explain increased viability/proliferation and invasiveness of cancer cells is widely documented in literature, and implicates downstream effectors such as MEK/ERK and AKT/TOR/rpS6 pathways. Indeed, in the revised manuscript, we show that phospho-ERK and phospho-S6 levels are dependent on EGFR activity only in AS43 cells carrying G842C-PLXNB2, but not in CUP cells bearing a WT receptor (Fig. 8C).

It is an interesting thought that the mutation may interfere with the auto-inhibitory head-to-tail formation of plexins that has been described by Kong et al. 2016 (PMID: 27397516; Fig. 3 therein), which should be considered. The relative conservation of the IPT3 domain inside the plexin structure should also be considered, see Junqueira Alves et al. 2019 (PMID: 30760850; Fig. 2 therein)

Re: We are grateful to the Reviewer for these comments and suggestions. Indeed, according to Kong et al., the IPT2 domains of Plexins of A-subfamily interact with the Sema domain of a dimerized homologous plexin, resulting in intermolecular receptor auto-inhibition. This suggests a complex multimolecular structure that maintains the plexins in the inactive state, until the engagement by dimeric semaphorin ligands triggers an activatory conformational change. Prompted by the Reviewer's comment, we verified along the trajectories of the MD simulations if the G842C mutation found in the IPT3 domain of Plexin-B2 may be capable of causing structural rearrangements also in the close by IPT2 domain. Unfortunately, the time course available in these experiments is limited to only 500 ns of simulation, which is likely insufficient to reveal this kind of conformational changes that may require a hundred microsecond to millisecond time scale (beyond the frame of MD simulation of the present system). We attach below, for the Reviewer, the RMSD plot for the IPT2 domain calculated in the "pseudo wild-type" G842C conformation, and for

the same mutant with scrambled disulfide bridges. As it can be seen, no significant fluctuations in the RMSD are reported on the IPT2 domain along the MD trajectories analyzed.

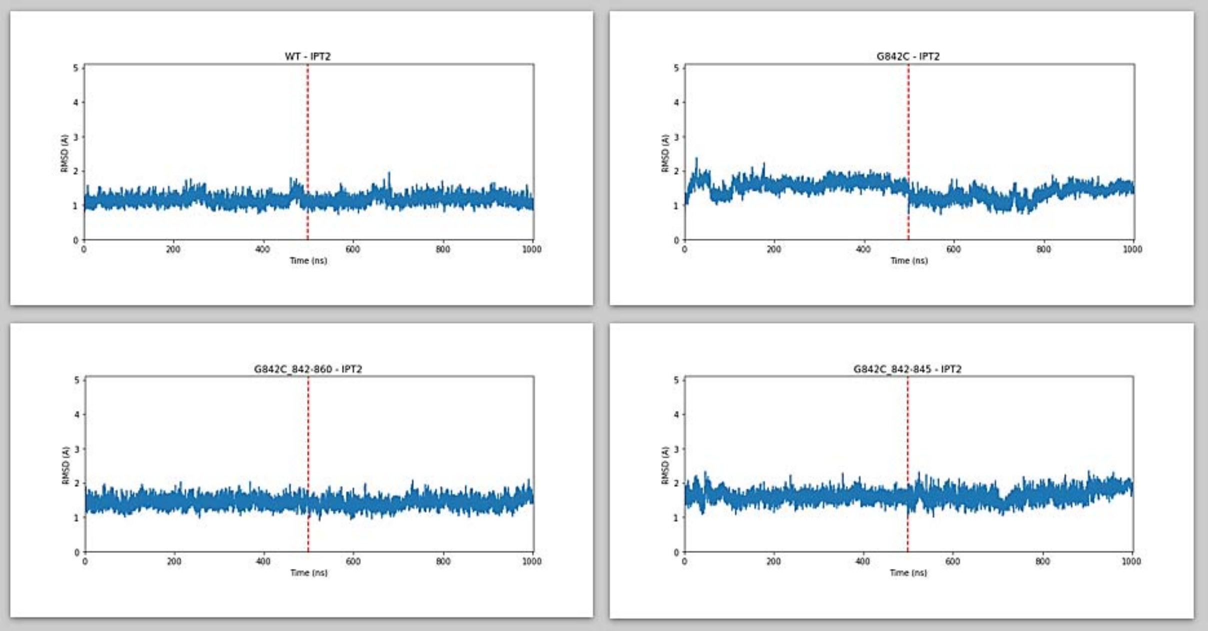

Nevertheless, taking in account also the other Reviewer's suggestion, about to the relative conservation of the IPT3 domain in the plexin family, it can be speculated that a mutation altering the stability of the tridimensional folds of this highly structured domain may indirectly impact also on the possibility for the neighboring domain IPT2 to interact with its binding partners with the same efficiency, and thereby possibly destabilize the auto-inhibitory intermolecular complex. We discussed these intriguing aspects in the revised manuscript in pages 13-14.

On a technical level, the data of cell-based assays and western blots is not convincing. In general, images are of low quality. It cannot be excluded that the effects are due to different expression levels of the Plexin-B2 forms in the cells, or other noise in the system, and not due to genuine GOF effects.

Re: We regret if some of the images included in the manuscript appeared with a low resolution. We have revised all source files and made sure images shown in the revised manuscript are of sufficient quality. Notably, we have shown in the paper western blot results representative of multiple experiments, as indicated in figure legend, and usually accompanied them with band quantification and statistical analysis. Phospho-EGFR levels have been normalized to total EGFR levels, while vinculin staining provided a reference of protein loading.

In the collapse assay, the single cells shown in Fig. 4C do not convince about the prevalent collapse phenotype in cells expressing G842C-Plexin-B2. The authors should show more cells and specify the criterion which they used to consider a collapsed cell, not only cells with one diameter lower than 40 µm were scored as collapsed (page 23), which seems quite arbitrary.

Re: We can understand Reviewer's concerns. However, though the collapsed cell phenotype is easily recognizable at microscopic examination, the morphometric analysis of collapse assays does not rely on a standard protocol in existing literature. This is largely due to systems variations depending on the cellular model analyzed in the experiments. We have previously applied cell diameter measurements as reliable proxy of the cell rounding typically associated with plexin-induced cellular collapse. However, this approach requires the manual identification of the cell

diameter to be measured; thus, in order to overcome this limitation, in the revised work we decided to employ an unbiased measurement of cell area by ImageJ software. In fact, collapsed cells retract adherent cellular processes and become rounded, thereby reducing the visible cellular area. However, it is inevitable to establish an arbitrary value below which the collapsed phenotype may be reliably assigned (here we took as discriminant an area below 2000 μm^2, which comprised the vast majority of the cells subjected to canonical ligand stimulation) (Fig.4B). Moreover, in order to provide a wider picture to the Reviewer (and the readers), we have included in supplemental data a large set of representative images of cells in the different groups, and a violin plot, which provides information on the prevalent distribution of individual values of cellular areas (new Figure S2 in Appendix).

Figure 5, 6 and 7 lack representative images.

Re: We believe that representative morphological images are crucial whenever the definition of a qualitative parameter may be debatable. However, data shown in Figures 5, 6 and 7 concern measurements of cell counts, cell viability, and tumor volume, which have been quantified by reliable consolidated methods described in the relative section of the manuscript and supported by a wide literature. We do not systematically take photographic images of these experiments, but rather process their quantification as described. We hope this approach may be accepted by the Reviewer.

In Fig. 8B panel is not possible to distinguish the cells.

Re: We have provided new and better images of the assays in the revised figure (now shown as Figure 7C); moreover, we have included in supplemental data these wide-field representative images in a large size (Appendix Figure S4B), as were used for results quantification by ImageJ software.

In Fig 8D panel the cells are very small and the images are of low quality.

Re: Images shown in Fig. 8D are low magnification images of semipermeable membranes of Transwell inserts, in which migrated cells have been revealed by crystal violet staining. Often, representative images are not shown for this kind of assays, and cell quantification is simply plotted in a graph. Here we thought to show representative images as well, but the experimental conditions do not allow achieving high resolution visualization of the individual cells, as the Reviewer can appreciate by representative photographs taken at high magnification from the same membranes analyzed in the paper (attached below). We feel that including these images in the paper may not be necessary, but we are ready to do so if requested.

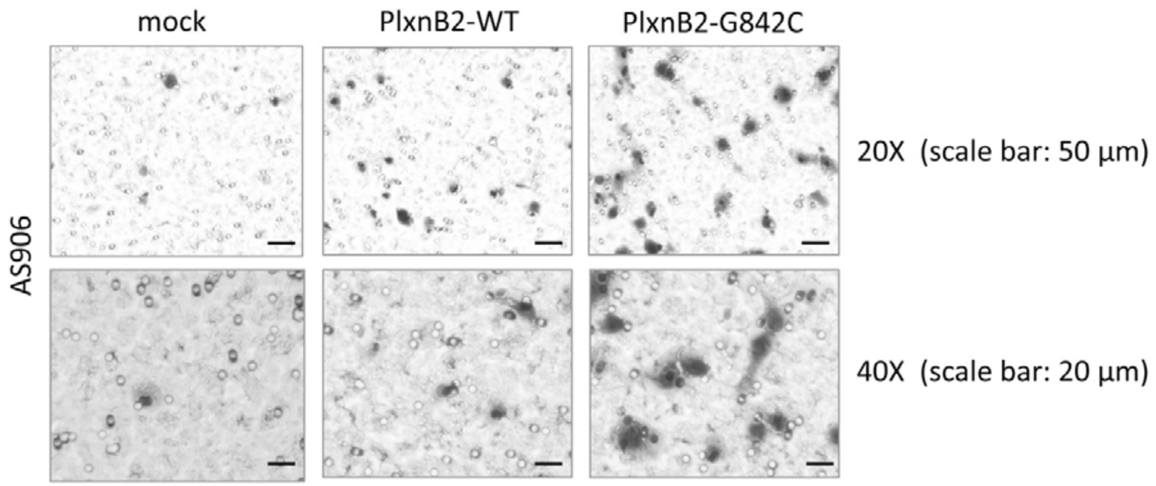

Fig. 9C lacks quantification.

The immunoprecipitation results shown in the figure had been repeated twice. However, also prompted by another reviewer, we made efforts to analyze more in depth this issue, by performing additional experiments in cells expressing endogenous or overexpressed receptors, and assaying reciprocal IP-WB protocols with anti-PlexinB2 and anti-EGFR antibodies. While the interaction between PlxnB2 and EGFR/ErbB1 was consistently confirmed in transfected cells, the quantitative difference between wild type and mutated PlxnB2 co-precipitation efficiency with EGFR was not consistently observed in all our further experiments. Based on these results, we decided to revise our claims in the paper, underscoring the hypothesis that PlxnB2 and EGFR interact transiently within a dynamic complex (see revised text in page 10). Previous studies have revealed the capacity of the homologous plexins PlxnB1 and PlxnB2 to interact with receptor tyrosine kinases ErbB2 and Met, and activate them upon on ligand stimulation. In our work, the novel association between PlxnB2-EGFR could be consistently revealed, however the presence of G842C mutation is not strictly required for the interaction, but is likely to act on PlxnB2 conformation, affecting its capacity to activate EGFR beyond ligand stimulation. For the considerations above, and in order to save space in the main figure for additional novel results emerging from Reviewers suggestions, we propose to move the results of these experiments in Expanded View section (Figure EV5-A).

Other comments:

1) The author should present the sequences for Plexin-B2 shRNAs used in the study. It is scientific best practice to use two independent shRNAs for knockdown, which should be performed here.

Re: In accordance with the Reviewer’s recommendation, we have further targeted PlxnB2 expression with an independent shRNA construct (TRCN0000048190), achieving less profound but still significant gene knock-down. This resulted in similar functional impact on AS43 cells carrying G842C-PLXNB2 (Figure EV3 panels D-E). Importantly, we have included shRNA sequences and product references for both constructs in the Methods section.

2) Western blots for Plexin-B2 should show both mature and precursor bands (see Artigiani et al.,

2003), not just the cut out upper band of the precursor form, but also the mature form, which should be more important than the precursor in regard to biological function.

Re: The Reviewer is right. Plexin-B2 protein may be found in cells either as monomeric precursor of approx. 250 kDa, or processed into a heterodimer, which includes two associated subunits of around 170 and 80 kDa respectively. Indeed, using an antibody directed against the C-terminal portion of the protein, both bands could be detected in our western blots, although only the predominant monomeric form was shown in the figures of the initial manuscript. Thus, in order to clarify this aspect, we have now included a full view of both PlxnB2 isoforms revealed in our western blots (see new Fig. 7, panel A for MCF7 and panel D for AS906). As it can be appreciated, it is confirmed that the wild type and mutant form of the plexin have been expressed at comparable levels also in this respect. In other figures, for practical reasons, we have displayed the most represented receptor isoform, with specific indication of the molecular weight.

3) Western blots should have size indicators in kDa.

Re: We have revised western blot images in the revised manuscript, including molecular weight indicators.

4) Micrographs need scale bars.

Re: We have completed the figures of the revised manuscript with scale bars, whenever missing.

5) Figs.5 and 7 legends need to be revised. The P stars do not correspond to those observed in the figure.

Re: Figures 5 and 7 in the initial submission (Fig. 5 and Fig. 6 in the revised manuscript) have been expanded and improved following Reviewer's comments; the legends have been revised, also taking in account the outcome of new statistical analyses with ANOVA. We apologize for any confusion in the text of the previous version.

6) The authors state that the stem cell frequency was strongly reduced in Plexin-B2-depleted AS43, but the Fig. 4B shows the opposite of that (shScramble 13% and shPlxnB2 25%).

Re: The Reviewer is right. We apologize for this confusion in the table attached below the graph, where the two values have actually been swapped. In fact, the descriptive text was correct, as shown in the graph, and the right values are: shScramble 25% and shPlxnB2 13% (as now shown in the revised Fig. 5B).

7) The authors use t-test to compare two groups in experimental settings that require comparisons of three groups or more. Ex: Figs. 5A, 6A, 7A-B should use two-way ANOVA. Figs. S2 and 9B require one-way ANOVA or non-parametric test.

Re: We thank the reviewer for this recommendation. Actually, upon checking our files, we realized that the statistical analysis of data shown in Fig. 5A and 6A (the latter is now shown as Figure 5C in the revised manuscript) was already done by 2-way ANOVA, although we erroneously reported t-test in the figure legend. On the other hand, data shown in Fig. 7A-B (now shown as Figure 6A-B) were re-analyzed by two-way ANOVA, and the figure and legend were appropriately corrected. Similarly, we reanalyzed by one-way ANOVA the data shown in Figs. S2 and 9B (now presented in Figure 7B and Figure 8B, respectively, in the revised manuscript).

8) Fig. 4C requires statistical treatment.

Re: As discussed above, in response to a previous Reviewer's comment, we have revised and expanded the analysis of cellular collapse induced in presence of wild type and mutated PlxnB2. In the revised Fig. 4B, is now included a statistical analysis (by Fisher's exact test) comparing each experimental condition with untreated cells expressing the wild-type receptor, which shows high significant difference in presence of G842C-PlxnB2. Moreover, in Appendix Fig. S2, we show a violin plot accounting for individual cell measurements, accompanied by one-way ANOVA statistical analysis.

9) Fig. 4A requires quantification.

Re: In the revised version, we indeed quantified the results of ligand binding assays, which proved to be not significantly different between wild-type and G842C-PlxnB2. This supported our conclusion that the higher cell-collapsing activity of the mutant receptor is rather due to ligand-independent conformational regulation. This complementary evidence is now shown in Appendix Figure S3.

10) The migration assays for MCF7 cancer cells are not properly described on page 13. I suggest move the Suppl. Fig.2 to Fig 8 and show representative images for that.

Re: We have reformatted Fig. 8 (now shown as Figure 7) and its legend in the revised version, as recommended by the Reviewer. As discussed above, representative images of cells fixed within Transwell membranes cannot be shown in a large format (or high magnification) in this main figure. However, we have included in supplemental data wide-field representative images of these migrated cells stained with crystal violet (Appendix Figure S4A), as were used for results quantification by ImageJ software.

11) Fig. 8D is not mentioned in the main text.

Re: We thank the Reviewer for noticing this mistake. We have corrected the reference to figure panels in the text.

12) The Co-IP images on Fig. 9C are not good for publication. The authors should provide better images, particularly for the total lysate.

Re: We apologize for the poor quality of western blots showing total lysates in the figure. We have reloaded the archived samples in a new gel, which yielded better resolved bands, now shown in Figure EV-5A. As discussed above, we have also performed additional IP experiments, by reciprocal immunoprecipitation of PlexinB2 with anti-EGFR antibodies. We show representative results of all these experiments in Figure EV-5A.

13) The authors should perform statistical tests for the ctrl groups of Fig 9D as well.

Re: We apologize for missing to include this information, which is now shown in expanded view Figure EV5. Consider that the results of a new series of similar experiments, done by treating cells with the EGFR inhibitor cetuximab (more specific for the non-mutated oncoprotein), are shown in the new main Figure 9D of the revised manuscript. Noteworthy, we have better specified in the figure legends that the statistical analysis has been done comparing inhibitor-treated vs. untreated cells per each condition; in fact, the specific aim of these experiments was to assess the dependence on EGFR of CUP cell invasiveness, in the presence of wild-type or mutated PlxnB2.

14) It is suggested that Table 1 should go to the Sup. Materials.

Re: We have moved a new enriched version of Table 1 in supplementary materials (Table EV1).

15) Possible grammar mistake: "Landscaping" means shaping the landscape. The authors probably want to say "survey of the landscape"

Re: We thank the Reviewer for this rightful correction that we have implemented in the revised manuscript.

Cited References

Damola A, Legendre A, Ball S, Masters JR, Williamson M. Function of mutant and wild-type plexinb1 in prostate cancer cells. Prostate. 2013 Sep;73(12):1326-35.

*Gerasimavicius L, Livesey BJ & Marsh JA. Loss-of-function, gain-of-function and dominant-negative mutations have profoundly different effects on protein structure. Nat Commun **13**, 3895 (2022).*

Giordano S, Corso S, Conrotto P, Artigiani S, Gilestro G, Barberis D, Tamagnone L, Comoglio PM. The semaphorin 4D receptor controls invasive growth by coupling with Met. Nat Cell Biol. 2002 Sep;4(9):720-4.

Le AP, Huang Y, Pingle SC, Kesari S, Wang H, Yong RL, Zou H, Friedel RH. Plexin-B2 promotes invasive growth of malignant glioma. Oncotarget. 2015 Mar 30;6(9):7293-304.

Li G, Ma L, Lu H, Cao G, Shao X, Liu Y, Li YX, Liu M, Yang H, Wang YL. Transactivation of Met signalling by semaphorin4D in human placenta: implications for the pathogenesis of preeclampsia. J Hypertens. 2018 Nov;36(11):2215-2225.

*Li Y, Zhang Y, Li X, Yi S, Xu J. Gain-of-Function Mutations: An Emerging Advantage for Cancer Biology. Trends Biochem Sci. **44**, 659-674 (2019).*

Peacock JW, Takeuchi A, Hayashi N, et al. SEMA3C drives cancer growth by transactivating multiple receptor tyrosine kinases via Plexin B1. EMBO Mol Med. 2018 Feb;10(2):219-238.

Soong J, Chen Y, Shustef EM, Scott GA. Sema4D, the ligand for Plexin B1, suppresses c-Met activation and migration and promotes melanocyte survival and growth. J Invest Dermatol. 2012 Apr;132(4):1230-8.

Sun T, Krishnan R, Swiercz JM. Grb2 mediates semaphorin-4D-dependent RhoA inactivation. J Cell Sci. 2012 Aug 1;125(Pt 15):3557-67.

Swiercz JM, Worzfeld T, Offermanns S. ErbB-2 and Met reciprocally regulate cellular signaling via plexin-B1. J Biol Chem. 2008 Jan 25;283(4):1893-901.

Worzfeld T, Swiercz JM, Looso M, Straub BK, Sivaraj KK, Offermanns S. ErbB-2 signals through Plexin-B1 to promote breast cancer metastasis. J Clin Invest. 2012 Apr;122(4):1296-305.

***Referee #3** (Remarks for Author):*

In this paper the authors have demonstrated that a novel activating mutation of the axon guidance gene PLXNB2 plays an important role in the development of human Cancers of Unknown Primary (CUP). Moreover, it is interested that the mutated PlxnB2 elicited EGFR-dependent invasiveness of CUP, which was blocked by EGFR inhibitor treatment. Overall, this is a very nice manuscript that shows new findings using human specimens. I have several questions that should be addressed, however.

1. Does the G842C-mutated PlxnB2 enhance the WT Plexin B2 signaling pathway or act on a different signaling pathway? If the former, do CUP cells with mutant PlexinB2 undergo the changes brought about by the PlexinB2-Sema4C signal? For example, collapsed phenotype similar to COS7, activation of RhoA / Rac1, enhancement of phosphorylation of MET in co-stimulation, etc.

Re: We are grateful to the Reviewer for his/her positive appreciation of our work. In fact, plexin signaling mechanisms are only partly understood, and PlexinB2 has been associated over the years to different effectors/signal transducers. Notably, all plexins seem to regulate integrin-based adhesion and cytoskeletal dynamics, which is the mechanism implicated in the typical "collapsing" response, which is rather specific for axons or plexin-overexpressing adherent cells. However, the same receptors have been found to interact with receptor tyrosine kinases, eliciting complementary biological activities, such as cancer cell invasive growth, angiogenesis or neurites extension (e.g. Chauvet et al, 2007; Franco et al, 2008; Swiercz et al, 2008; Cagnoni et al, 2014). Since in our study we found for the first time that G842C-mutated PlxnB2 elicits EGFR activation, prompted by the Reviewer's comment we aimed at assessing if this same signaling cascade may also be triggered by the main PlxnB2 ligand Sema4C, in cells expressing wild type or mutant receptors. However, our preliminary results did not support this hypothesis, rather confirming our conclusion that this mutation is promoting a novel Sema4C-independent PlexinB2 signaling mechanism. Interestingly, other studies have recently reported signaling mechanisms mediated by plexins (including PlexinB2) which are not necessarily regulated by semaphorins (Mehta et al, 2020; Jiang et al, 2021; Yang et al, 2002). This intriguing issue is now discussed in the revised manuscript on page 13.

2. Is the association of G842C-mutated PlxnB2 with EGFR found in HEK293T also confirmed in CUP cells?

Re: The observation of PlxnB2-dependent control of EGFR phosphorylation suggested us that the two receptors might be stably associated in a complex. Indeed, this hypothesis was supported by the co-immunoprecipitation of the two proteins overexpressed in HEK293T cells. However, when we tried the new experiment suggested by the Reviewer, we could not detect plexin association with endogenous EGFR in CUP cells. These findings may reflect a transient or indirect association of the two receptors in CUP cells, resulting in inefficient pull-down of the complexes at the steady state. Notably, we discussed this issue in the revised manuscript (in page 10), tuned down our conclusions on this point, and moved revised representative data to Expanded View Figure EV5-A.

3. In AS43, the mutant PlexinB2 promotes cell proliferation, but in MCF7, PlexinB2 overexpression (either wt or mutated) did not affect the growth rate. Is this difference the nature of the cells?

Re: We fully understand the Reviewer's concern. Indeed, it should be considered that the cellular models, as well as the experimental conditions are substantially different. In fact, AS43 cells are

maintained in total absence of serum and growth factors and their proliferative self-sufficiency is supposedly sustained by intrinsic regulatory circuits. As further discussed below, we believe that, in this setting, the expression of G842C-mutated PlxnB2 provides a non-conventional mechanism for the activation of EGFR signaling cascade, sustaining cell growth. Similarly, the experimental expression of the mutated plexin seems to provide an additional mechanism of EGFR activation and growth stimulation in other CUP cells (AS901, AS906, AS67), which are also propagated in absence of serum or exogenous growth factors. In contrast, MCF7 cell proliferation is not sustained in culture in the absence of fetal serum, and the expression of the mutated PlxnB2 was not sufficient to significantly affect basal growth rate, although cell migration was increased. The latter results supported the evidence of a functional gain associated with G842C-PlxnB2 mutation also in MCF7 cells; however, at the present stage, we cannot illustrate whether the implicated mechanism is identical to that demonstrated in CUP cells.

4. Is only cell invasion dependent on EGFR activity? Does it affect cell proliferation, stemness, or migration?

Re: This is a crucial question that we aimed at addressing in our revised manuscript. In particular, we found that the viability and clonogenic capacity (representing stemness) of CUP cells carrying G842C-mutated, but not wild-type, PlxnB2 is dependent on EGFR signaling, as demonstrated by its blockade with the monoclonal antibody cetuximab (new Fig. 9A-9B-9C). Consistently, the treatment with EGFR inhibitors abated ERK and rpS6 phosphorylation in cells carrying G842C-PLXNB2, but not in cells bearing the wild-type receptor (new Fig. 8C). Moreover, the increase in cell invasiveness driven by the transduction of mutated PlxnB2 was abrogated by the treatment with EGFR inhibitors (either cetuximab or erlotinib), indicating EGFR was mechanistically responsible for it (new Fig. 9D and Figure EV5-B).

5. Fig 8B, authors mentioned that wound healing by cells overexpressing wild type PlxnB2 was slightly but not significantly increased, compared to controls, but it seems that there is a significant difference in the figure.

Re: The Reviewer is right. We have now amended the description of these data in the main text. Indeed, as shown in the figure, the difference between controls and wild type PlxnB2-expressing cells is significant, although less prominent than for mutated G842-PlxnB2. These results are consistent with those obtained in CUP cells (see Fig. 8D).

6. "Fig8C" in the text is "8D".

Re: We thank the Reviewer for noticing this mistake. We have corrected the reference to figure panels in the text.

Cited References

Cagnoni G, Tamagnone L. Semaphorin receptors meet receptor tyrosine kinases on the way of tumor progression. Oncogene. 2014 Oct 2;33(40):4795-802.

Chauvet S, Cohen S, Yoshida Y, Fekrane L, Livet J, Gayet O, Segu L, Buhot MC, Jessell TM, Henderson CE, Mann F. Gating of Sema3E/PlexinD1 signaling by neuropilin-1 switches axonal repulsion to attraction during brain development. Neuron. 2007 Dec 6;56(5):807-22.

Franco M, Tamagnone L. Tyrosine phosphorylation in semaphorin signalling: shifting into overdrive. EMBO Rep. 2008 Sep;9(9):865-71.

Jiang, C., Javed, A., Kaiser, L. et al. Mechanochemical control of epidermal stem cell divisions by B-plexins. Nat Commun 12, 1308 (2021).

Mehta V, Pang KL, Rozbesky D, Nather K, Keen A, Lachowski D, Kong Y, Karia D, Ameismeier M, Huang J, Fang Y, Del Rio Hernandez A, Reader JS, Jones EY, Tzima E. The guidance receptor plexin D1 is a mechanosensor in endothelial cells. Nature. 2020 Feb;578(7794):290-295.

Swiercz JM, Worzfeld T, Offermanns S. ErbB-2 and Met reciprocally regulate cellular signaling via plexin-B1. J Biol Chem. 2008 Jan 25;283(4):1893-901.

Yang, H., Yuan, L., Ibaragi, S. et al. Angiogenin and plexin-B2 axis promotes glioblastoma progression by enhancing invasion, vascular association, proliferation and survival. Br J Cancer 127, 422–435 (2022).

14th Dec 2022

Dear Prof. Tamagnone,

Thank you for the submission of your manuscript to EMBO Molecular Medicine. We have now received the enclosed reports from the two referees who re-reviewed your manuscript. As you will see below, they are now supportive of publication, and I am therefore pleased to inform you that we will be able to accept your manuscript once the following editorial points will be addressed:

1/ Main manuscript text:
- Please address the queries from our data editors in track changes mode in the main manuscript file labelled 'Data edited MS file'. Kindly use this file for any further modification, remove the blue text and only keep in track changes mode any new modification.
- Would you consider shortening your title? While this is not mandatory, we usually recommend 100 characters maximum, including space.
- Please provide up to 5 keywords.
- Please remove "not shown" (p.9 and 10): As per our guidelines on "Unpublished Data", all data referred to in the paper should be displayed in the main or Expanded View figures (see also: https://www.embopress.org/page/journal/17574684/authorguide#unpublisheddata)
- Material and methods:
o Please provide the antibody dilutions.
o Please provide the age and gender of the mice, as well as their housing and husbandry conditions. Please also indicate the origin of the mice.
o Statistics: please provide information as indicated in the checklist (exclusion criteria, blinding, etc)
o Please indicate whether the cells were tested for mycoplasma contamination.
- Author contribution: CRediT has replaced the traditional author contributions section because it offers a systematic machine-readable author contributions format that allows for more effective research assessment. Please remove the Authors Contributions from the manuscript and use the free text boxes beneath each contributing author's name in our system to add specific details on the author's contribution. More information is available in our guide to authors.
- Acknowledgements: Please make sure that funding information provided in the manuscript matches the information provided in the submission system.
- Please include the "Disclosure statement and competing interests" after the acknowledgements with a heading. We updated our journal's competing interests policy in January 2022 and request authors to consider both actual and perceived competing interests. Please review the policy https://www.embopress.org/competing-interests and update your competing interests if necessary.
- Please reformat the references: they should be in alphabetical order and list 10 authors before et al.

2/ Figures and Appendix:
- Please indicate for all figures or in their legends the exact p= values, not a range. Some people found that to keep the figures clear, providing a supplemental table in an Appendix with all exact p-values was preferable. You are welcome to do this if you want to.
- Please make sure that all figures are referenced in the manuscript text. A callout is currently missing for Fig. 5D.
- Dataset EV legends: The legend for Table EV1 should be removed from the manuscript and added to the table. Table EV2 should be made Dataset EV1, the legend should be removed from the manuscript file and added to the excel file in a separate tab.
- The nomenclature of Appendix figures should be changed to Appendix Figure S1, etc.

3/ At EMBO Press we encourage authors to include the source data for figure panels that show essential data. Numerical data should be provided as individual .xls or .csv files (including a tab describing the data). For blots or microscopy, uncropped images should be submitted (using a zip archive if multiple images need to be supplied for one panel). Additional information on source data and instruction on how to label the files are available at
.

4/ Checklist:
- Newly created materials: please fill in the left column and remove "new materials ... reasonable commonly applied conditions".
- Cell materials: please indicate whether the cells were tested for mycoplasma contamination.
- Experimental animals: please fill in the "housing and husbandry conditions" line.
- Please fill in the Human research participants part.
- Statistics: please select an answer in the left columns.
- Data Availability: please select an answer in the left column when you fill in the right column.

5/ Synopsis: Please correct the format of your synopsis to have a short stand first (maximum of 300 characters, including space) as well as 2-5 one-sentences bullet points that summarizes the paper (maximum of 30 words/bullet point).
Please resize your visual abstract as a PNG file 550 px wide x 300-600 px high.

6/ As part of the EMBO Publications transparent editorial process initiative (see our Editorial at http://embomolmed.embopress.org/content/2/9/329), EMBO Molecular Medicine will publish online a Review Process File (RPF) to accompany accepted manuscripts.
This file will be published in conjunction with your paper and will include the anonymous referee reports, your point-by-point response and all pertinent correspondence relating to the manuscript. Let us know whether you agree with the publication of the RPF and as here, if you want to remove or not any figures from it prior to publication.
Please note that the Authors checklist will be published at the end of the RPF.

I look forward to receiving your revised manuscript.

Yours sincerely,

Lise Roth

Lise Roth, PhD
Senior Editor
EMBO Molecular Medicine

***** Reviewer's comments *****

Referee #2 (Remarks for Author):

I thank the authors for their careful consideration of comments for their revision.

Referee #3 (Remarks for Author):

The authors have added sufficient data and appropriately addressed concerns. The revised manuscript is clinically meaningful and worthy of publication.

The authors addressed the minor editorial issues.

11th Jan 2023

Dear Prof. Tamagnone,

Thank you for sending your revised file.
I am pleased to inform you that your manuscript is now accepted for publication and will be sent to our publisher to be included in the next available issue of EMBO Molecular Medicine!

Please read below for additional IMPORTANT information regarding your article, its publication and the production process.

Congratulations on your interesting work,

With kind regards,

Lise Roth

Lise Roth, Ph.D
Senior Editor
EMBO Molecular Medicine

Follow us on Twitter @EmboMolMed
Sign up for eTOCs at embopress.org/alertsfeeds

*** *** *** IMPORTANT INFORMATION *** *** ***

SPEED OF PUBLICATION
The journal aims for rapid publication of papers, using using the advance online publication "Early View" to expedite the process: A properly copy-edited and formatted version will be published as "Early View" after the proofs have been corrected. Please help the Editors and publisher avoid delays by providing e-mail address(es), telephone and fax numbers at which author(s) can be contacted.

Should you be planning a Press Release on your article, please get in contact with embomolmed@wiley.com as early as possible, in order to coordinate publication and release dates.

LICENSE AND PAYMENT:

All articles published in EMBO Molecular Medicine are fully open access: immediately and freely available to read, download and share.

EMBO Molecular Medicine charges an article processing charge (APC) to cover the publication costs. You, as the corresponding author for this manuscript, should have already received a quote with the article processing fee separately. Please let us know in case this quote has not been received.

Once your article is at Wiley for editorial production you will receive an email from Wiley's Author Services system, which will ask you to log in and will present you with the publication license form for completion. Within the same system the publication fee can be paid by credit card, an invoice, pro forma invoice or purchase order can be requested.

Payment of the publication charge and the signed Open Access Agreement form must be received before the article can be published online.

PROOFS

You will receive the proofs by e-mail approximately 2 weeks after all relevant files have been sent o our Production Office.

Please return them within 48 hours and if there should be any problems, please contact the production office at embopressproduction@wiley.com.

Please inform us if there is likely to be any difficulty in reaching you at the above address at that time. Failure to meet our deadlines may result in a delay of publication.

All further communications concerning your paper proofs should quote reference number EMM-2022-16104-V3 and be directed to the production office at embopressproduction@wiley.com.

EMBO Press Author Checklist

Corresponding Author Name: Luca Tamagnone - Enrico Giraudo
Journal Submitted to: EMBO Molecular Medicine
Manuscript Number: EMM-2022-16104

USEFUL LINKS FOR COMPLETING THIS FORM
The EMBO Journal - Author Guidelines
EMBO Reports - Author Guidelines
Molecular Systems Biology - Author Guidelines
EMBO Molecular Medicine - Author Guidelines

Reporting Checklist for Life Science Articles (updated January 2022)
This checklist is adapted from Materials Design Analysis Reporting (MDAR) Checklist for Authors. MDAR establishes a minimum set of requirements in transparent reporting in the life sciences (see Statement of Task: 10.31222/osf.io/9sm4x). Please follow the journal's guidelines in preparing your manuscript.
Please note that a copy of this checklist will be published alongside your article.

Abridged guidelines for figures
1. Data
The data shown in figures should satisfy the following conditions:
- the data were obtained and processed according to the field's best practice and are presented to reflect the results of the experiments in an accurate and unbiased manner.
- ideally, figure panels should include only measurements that are directly comparable to each other and obtained with the same assay.
- plots include clearly labeled error bars for independent experiments and sample sizes. Unless justified, error bars should not be shown for technical replicates.
- if n<5, the individual data points from each experiment should be plotted. Any statistical test employed should be justified.
- Source Data should be included to report the data underlying figures according to the guidelines set out in the authorship guidelines on Data Presentation.

2. Captions
Each figure caption should contain the following information, for each panel where they are relevant:
- a specification of the experimental system investigated (eg cell line, species name).
- the assay(s) and method(s) used to carry out the reported observations and measurements.
- an explicit mention of the biological and chemical entity(ies) that are being measured.
- an explicit mention of the biological and chemical entity(ies) that are altered/varied/perturbed in a controlled manner.
- the exact sample size (n) for each experimental group/condition, given as a number, not a range;
- a description of the sample collection allowing the reader to understand whether the samples represent technical or biological replicates (including how many animals, litters, cultures, etc.).
- a statement of how many times the experiment shown was independently replicated in the laboratory.
- definitions of statistical methods and measures:
  - common tests, such as t-test (please specify whether paired vs. unpaired), simple χ2 tests, Wilcoxon and Mann-Whitney tests, can be unambiguously identified by name only, but more complex techniques should be described in the methods section;
  - are tests one-sided or two-sided?
  - are there adjustments for multiple comparisons?
  - exact statistical test results, e.g., P values = x but not P values < x;
  - definition of 'center values' as median or average;
  - definition of error bars as s.d. or s.e.m.

Please complete ALL of the questions below.
Select "Not Applicable" only when the requested information is not relevant for your study.

Materials

Newly Created Materials	Information included in the manuscript?	In which section is the information available? (Reagents and Tools Table, Materials and Methods, Figures, Data Availability Section)
New materials and reagents need to be available; do any restrictions apply?	Not Applicable	

Antibodies	Information included in the manuscript?	In which section is the information available? (Reagents and Tools Table, Materials and Methods, Figures, Data Availability Section)
For **antibodies** provide the following information:<br>- Commercial antibodies: RRID (if possible) or supplier name, catalogue number and or/clone number<br>- Non-commercial: RRID or citation	Yes	Materials and Methods

DNA and RNA sequences	Information included in the manuscript?	In which section is the information available? (Reagents and Tools Table, Materials and Methods, Figures, Data Availability Section)
**Short novel DNA or RNA including primers, probes**: provide the sequences.	Yes	Materials and Methods

Cell materials	Information included in the manuscript?	In which section is the information available? (Reagents and Tools Table, Materials and Methods, Figures, Data Availability Section)
**Cell lines:** Provide species information, strain. Provide accession number in repository **OR** supplier name, catalog number, clone number, and/**OR** RRID.	Yes	Materials and Methods
**Primary cultures:** Provide species, strain, sex of origin, genetic modification status.	Yes	Materials and Methods (details in referenced prior publication)
Report if the cell lines were recently **authenticated** (e.g., by STR profiling) and tested for mycoplasma contamination.	Yes	Materials and Methods

Experimental animals	Information included in the manuscript?	In which section is the information available? (Reagents and Tools Table, Materials and Methods, Figures, Data Availability Section)
**Laboratory animals or Model organisms:** Provide species, strain, sex, age, genetic modification status. Provide accession number in repository **OR** supplier name, catalog number, clone number, **OR** RRID.	Yes	Materials and Methods
**Animal observed in or captured from the field:** Provide species, sex, and age where possible.	Not Applicable	
Please detail **housing and husbandry conditions**.	Yes	Materials and Methods

Plants and microbes	Information included in the manuscript?	In which section is the information available? (Reagents and Tools Table, Materials and Methods, Figures, Data Availability Section)
**Plants:** provide species and strain, ecotype and cultivar where relevant, unique accession number if available, and source (including location for collected wild specimens).	Not Applicable	
**Microbes:** provide species and strain, unique accession number if available, and source.	Not Applicable	

Human research participants	Information included in the manuscript?	In which section is the information available? (Reagents and Tools Table, Materials and Methods, Figures, Data Availability Section)
If collected and within the bounds of privacy constraints report on age, sex and gender or ethnicity for all study participants.	Yes	Table EV1

Core facilities	Information included in the manuscript?	In which section is the information available? (Reagents and Tools Table, Materials and Methods, Figures, Data Availability Section)
If your work benefited from core facilities, was their service mentioned in the acknowledgments section?	Not Applicable	

Design

Study protocol	Information included in the manuscript?	In which section is the information available? (Reagents and Tools Table, Materials and Methods, Figures, Data Availability Section)
If study protocol has been **pre-registered, provide DOI in the manuscript**. For clinical trials, provide the trial registration number **OR** cite DOI.	Not Applicable	
Report the **clinical trial registration number** (at ClinicalTrials.gov or equivalent), where applicable.	Not Applicable	

Laboratory protocol	Information included in the manuscript?	In which section is the information available? (Reagents and Tools Table, Materials and Methods, Figures, Data Availability Section)
Provide DOI OR other citation details if **external detailed step-by-step protocols** are available.	Not Applicable	

Experimental study design and statistics	Information included in the manuscript?	In which section is the information available? (Reagents and Tools Table, Materials and Methods, Figures, Data Availability Section)
Include a statement about **sample size** estimate even if no statistical methods were used.	Yes	Sample size was estimated according to experimental experience and prior scientific literature, taking in account sample/parameter intrinsic variablity.
Were any steps taken to minimize the effects of subjective bias when allocating animals/samples to treatment (e.g. **randomization procedure**)? If yes, have they been described?	Yes	No randomization is required (or applicable) for the described experiments
Include a statement about **blinding** even if no blinding was done.	Yes	In general, blinding was not applied, but data analysis was validated by at least two scientists.
Describe **inclusion/exclusion criteria** if samples or animals were excluded from the analysis. Were the criteria pre-established?<br><br>If sample or data points were omitted from analysis, report if this was due to attrition or intentional exclusion and provide justification.	Yes	Results of experiments in which appropriate negative/positive controls were not validated were excluded from the analysis.
For every figure, are **statistical tests** justified as appropriate? Do the data meet the assumptions of the tests (e.g., normal distribution)? Describe any methods used to assess it. Is there an estimate of variation within each group of data? Is the variance similar between the groups that are being statistically compared?	Yes	Figure legends

Sample definition and in-laboratory replication	Information included in the manuscript?	In which section is the information available? (Reagents and Tools Table, Materials and Methods, Figures, Data Availability Section)
In the figure legends: state number of times the experiment was **replicated** in laboratory.	Yes	Figure Legends
In the figure legends: define whether data describe **technical or biological replicates**.	Yes	Figure Legends

Ethics

Ethics	Information included in the manuscript?	In which section is the information available? (Reagents and Tools Table, Materials and Methods, Figures, Data Availability Section)
Studies involving **human participants**: State details of **authority granting ethics approval** (IRB or equivalent committee(s), provide reference number for approval.	Yes	Materials and Methods
Studies involving **human participants**: Include a statement confirming that **informed consent** was obtained from all subjects and that the experiments conformed to the principles set out in the WMA Declaration of Helsinki and the Department of Health and Human Services Belmont Report.	Yes	Materials and Methods
Studies involving **human participants:** For publication of **patient photos**, include a statement confirming that consent to publish was obtained.	Not Applicable	
Studies involving experimental **animals**: State details of **authority granting ethics approval** (IRB or equivalent committee(s), provide reference number for approval. Include a statement of compliance with ethical regulations.	Yes	Materials and Methods
Studies involving **specimen and field samples:** State if relevant **permits** obtained, provide details of authority approving study; if none were required, explain why.	Not Applicable	

Dual Use Research of Concern (DURC)	Information included in the manuscript?	In which section is the information available? (Reagents and Tools Table, Materials and Methods, Figures, Data Availability Section)
Could your study fall under dual use research restrictions? Please check biosecurity documents and list of **select agents and toxins** (CDC): https://www.selectagents.gov/sat/list.htm	Not Applicable	
If you used a select agent, is the security level of the lab appropriate and reported in the manuscript?	Not Applicable	
If a study is subject to dual use research of concern regulations, is the name of the **authority granting approval and reference number** for the regulatory approval provided in the manuscript?	Not Applicable	

Reporting

The MDAR framework recommends adoption of discipline-specific guidelines, established and endorsed through community initiatives. Journals have their own policy about requiring specific guidelines and recommendations to complement MDAR.

Adherence to community standards	Information included in the manuscript?	In which section is the information available? (Reagents and Tools Table, Materials and Methods, Figures, Data Availability Section)
State if relevant guidelines or checklists (e.g., **ICMJE, MIBBI, ARRIVE, PRISMA**) have been followed or provided.	Not Applicable	
For **tumor marker prognostic studies,** we recommend that you follow the **REMARK** reporting guidelines (see link list at top right). See author guidelines, under 'Reporting Guidelines'. Please confirm you have followed these guidelines.	Not Applicable	
For **phase II and III randomized controlled trials**, please refer to the **CONSORT** flow diagram (see link list at top right) and submit the CONSORT checklist (see link list at top right) with your submission. See author guidelines, under 'Reporting Guidelines'. Please confirm you have submitted this list.	Not Applicable	

Data Availability

Data availability	Information included in the manuscript?	In which section is the information available? (Reagents and Tools Table, Materials and Methods, Figures, Data Availability Section)
Have **primary datasets** been deposited according to the journal's guidelines (see 'Data Deposition' section) and the respective accession numbers provided in the Data Availability Section?	Yes	Data Availability section
Were **human clinical and genomic datasets** deposited in a public access-controlled repository in accordance to ethical obligations to the patients and to the applicable consent agreement?	Yes	Data Availability section
Are **computational models** that are central and integral to a study available without restrictions in a machine-readable form? Were the relevant accession numbers or links provided?	Yes	Weblinks to publicly available datasets have been indicated in Materials and Methods section
If publicly available data were reused, provide the respective **data citations in the reference list**.	Yes	Weblinks to publicly available datasets have been indicated in Materials and Methods section
